# *Brucella microti*: the genome sequence of an emerging pathogen

**DOI:** 10.1186/1471-2164-10-352

**Published:** 2009-08-04

**Authors:** Stéphane Audic, Magali Lescot, Jean-Michel Claverie, Holger C Scholz

**Affiliations:** 1 Laboratoire Information Génomique et Structurale, CNRS – UPR2589, Aix-Marseille University, Institut de Microbiologie de la Méditerranée (IM2, IFR-88), Parc Scientifique de Luminy – 163 Avenue de Luminy – Case 934 – FR- 13288, Marseille cedex 09, France; 2 Bundeswehr Institute of Microbiology, Neuherbergstrasse 11, D-80937 Munich, Germany

## Abstract

**Background:**

Using a combination of pyrosequencing and conventional Sanger sequencing, the complete genome sequence of the recently described novel *Brucella *species, *Brucella microti*, was determined. *B. microti *is a member of the genus *Brucella *within the *Alphaproteobacteria*, which consists of medically important highly pathogenic facultative intracellular bacteria. In contrast to all other *Brucella *species, *B. microti *is a fast growing and biochemically very active microorganism with a phenotype more similar to that of *Ochrobactrum*, a facultative human pathogen. The atypical phenotype of *B. microti *prompted us to look for genomic differences compared to other *Brucella *species and to look for similarities with *Ochrobactrum*.

**Results:**

The genome is composed of two circular chromosomes of 2,117,050 and 1,220,319 base pairs. Unexpectedly, we found that the genome sequence of *B. microti *is almost identical to that of *Brucella suis *1330 with an overall sequence identity of 99.84% in aligned regions. The most significant structural difference between the two genomes is a bacteriophage-related 11,742 base pairs insert only present in *B. microti*. However, this insert is unlikely to have any phenotypical consequence. Only four protein coding genes are shared between *B. microti *and *Ochrobactrum anthropi *but impaired in other sequenced *Brucella*. The most noticeable difference between *B. microti *and other *Brucella *species was found in the sequence of the 23S ribosomal RNA gene. This unusual variation could have pleiotropic effects and explain the fast growth of *B. microti*.

**Conclusion:**

Contrary to expectations from the phenotypic analysis, the genome sequence of *B. microti *is highly similar to that of known *Brucella *species, and is remotely related to the one of *O. anthropi*. How the few differences in gene content between *B. microti *and *B. suis *1330 could result in vastly different phenotypes remains to be elucidated. This unexpected finding will complicate the task of identifying virulence determinants in the *Brucella *genus. The genome sequence of *B. microti *will serve as a model for differential expression analysis and complementation studies. Our results also raise some concerns about the importance given to phenotypical traits in the definition of bacterial species.

## Background

The genus *Brucella *comprises important mammal and human pathogens. Low infectious doses (10 to 100 bacteria, [1]), transmission through aerosols, and a difficult treatment of the disease by antibiotics, have led *Brucella *to be classified as potential bioterrorism agents. The genus *Brucella *[2] belongs to the family *Brucellaceae *within the order *Rhizobiales *of the *Alphaproteobacteria*. *Ochrobactrum*, a soil living facultative human pathogen, is the most closely related genus [3] with a 16S rRNA gene sequence [Genbank: U70978] more than 98% identical to that of *Brucella *spp.

Characterized *Brucella *comprise six classical *Brucella *species, *B. melitensis*, *B. abortus*, *B. ovis*, *B. canis*, *B. suis*, and *B. neotomae*, two species of marine mammal origin, namely *B. pinnipedialis *and *B. ceti*, and the recently described species *B. microti *and *B. inopinata*. *B. microti *was initially isolated from systemically diseased common voles (*Microtus arvalis*) in the Czech Republic [4]. More recently, it has also been isolated from red foxes in Lower Austria [5] and even directly from soil in the same geographical area [6]. Importantly, *B. microti *is thus the only *Brucella *species with a known reservoir outside of its mammalian host. Recent and frequent isolations of *B. microti *from different animals from different geographical regions indicate that *B. microti *could constitute an emerging pathogen. All *Brucella *species are genetically highly related, exhibiting identical 16S rRNA and *rec*A gene sequences [7]. In fact, DNA-DNA hybridization studies, today's gold standard for bacterial species delineation, suggest that all *Brucella *spp. should be unified into a single species (*B. melitensis*) and not be regarded as different species [8]. The high genetic relatedness of all *Brucella *species was further confirmed by whole genome sequencing [9-11].

Although *Brucella *spp. are facultative intracellular bacteria adapted to specific mammalian hosts, the whole genome sequence of *B. suis *revealed a clear similarity to that of soil bacteria associated with plants, such as *Agrobacterium *and *Rhizobium *[11]. It was therefore speculated that the mammal host-adapted *Brucella *species evolved from a plant-associated soil living ancestor. The recent isolation of *B. microti *directly from soil supported this hypothesis. In addition to its persistence in soils, *B. microti *is unique in sharing other phenotypic traits with its closest phylogenetic relative *Ochrobactrum*. In particular, *B. microti *is biochemically highly active, at odds with other *Brucella *species, but a common feature among *Ochrobactrum *species. We therefore speculated that *B. microti *might represent an intermediary stage in *Brucella *evolution, closer to the *Brucella*/*Ochrobactrum *common ancestor. To substantiate this hypothesis, the whole genome sequence of *B. microti *was determined and compared to that of other *Brucella *and to the genome sequence of *O. anthropi*. Differences in gene content that could explain *B. microti *phenotypic peculiarities were carefully studied.

## Results

The genome sequence of *Brucella microti *CCM 4915^T ^was determined (25× coverage) by shotgun analysis using the GS-FLX pyrosequencing technology and direct Sanger sequencing of remaining gaps. The genome is composed of two circular chromosomes of lengths 2,117,050 bp (base-pairs) and 1,220,319 bp. We predicted the presence of 3,291 protein coding genes, 55 tRNAs and 9 ribosomal RNAs. A comparison with the other *Brucella *genomes revealed the presence of 60 pseudogenes.

### Comparison of genome structures

**Figure 1:**
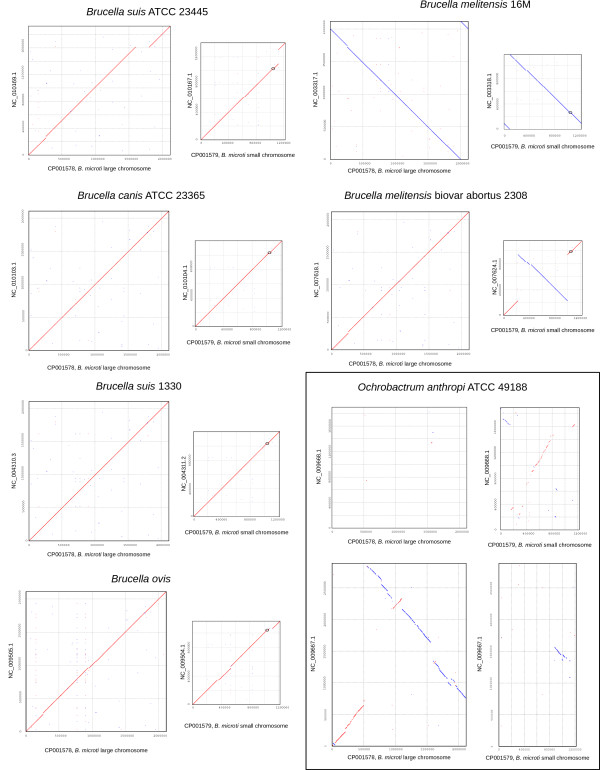
**Dotplots of 7 *Brucella *and *Ochrobactrum anthropi *genomes against the two chromosomes of *B. microti***. *B. microti *chromosomes are in abscissa of each plot and the corresponding chromosomes of target genomes are in ordinate. In chromosome 2 plots, the 12 kbp region specific to *B. microti *is circled. Plots for *B. abortus *S19 and *B. abortus *9–941 are not shown because of their similarity to the plot for *B. melitensis *biovar abortus 2308. In the case of *O. anthropi*, the dotplots of the two chromosomes of *B. microti *against the 2 large chromosomes of *O. anthropi *are shown. *O. anthropi *plasmids are not shown as they have no similarity with *B. microti *chromosomes.

**Figure 2:**
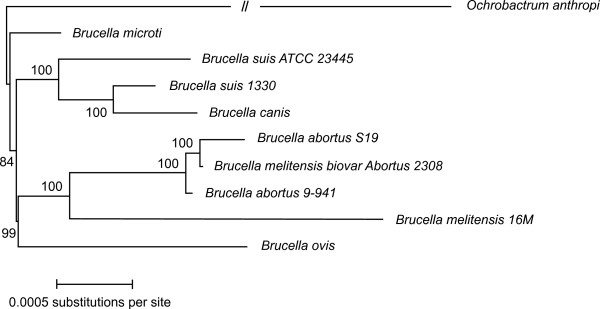
**Phylogenetic representation of the alignment of 1,486 groups of orthologous genes from 8 available *Brucella *genome sequences and that of *O. anthropi***. The long branch leading to *O. anthropi *has been shortened. Even though *B. suis *and *B. microti *are not found within the same clade, they both exhibit a slower evolution rate than most other *Brucella *species (as shown by their short branch length) resulting in a high overall similarity at the genome sequence level.

Dotplots (Figure 1) of the chromosomes of *B. microti *against the 8 *Brucella *genome sequences available at the time of writing and the genome sequence of *O. anthropi *show that: i) the overall genome structures of *Brucella *are remarkably conserved, ii) *Brucella *genomes are markedly different from that of *O. anthropi *and iii) based on this genome structure only, *B. microti *is more similar to *B. suis *1330, *B. canis *and *B. melitensis *16M than to the other genomes with which it has at least one major difference. Consistently, phylogenetic reconstruction based on a set of 1,486 orthologous genes clearly placed *B. microti *closer to *B. suis *1330 than to any other *Brucella *(Figure 2). We thus based our subsequent analyses on the detailed comparison of *B. microti *and *B. suis *1330.

### Global alignment of the chromosomes

The global alignment of *B. suis *1330 and *B. microti *chromosomes revealed their almost perfect co-linearity. On chromosome 1, we identified 270 indels (insertions or deletions) of one or more base-pairs in the alignment (163 insertions in *B. microti *and 107 in *B. suis *1330). On chromosome 2, we identified 135 indels (75 insertions in *B. microti *and 60 insertions in *B. suis *1330). Insertions in *B. microti *range in size from 1 bp (61 occurrences) to 845 bp on chromosome 1, and from 1 bp (32 occurrences) to 11,742 bp on chromosome 2 (the second largest insertion being 1071 bp long). Insertions in *B. suis *1330 range in size from 1 bp (57 occurrences) to 844 bp on chromosome 1 and from 1 bp (29 occurrences) to 510 bp on chromosome 2. The global alignment of the chromosomes is given as Additional Files 1 and 2 and the list of indels between *B. microti *and *B. suis *1330 is reported in Additional file 3.

Out of a total of 3,312,414 aligned positions, we observed 5213 SNPs, corresponding to 0.16% nucleotide difference between the two species. In comparison, alignment of *B. suis *1330 with *B. melitensis *16M revealed a total of 7307 SNP for a total of 3,237,820 aligned nucleotides (0.23%).

### The 12 kbp insertion

The largest difference between the genome of *B. suis *1330 and that of *B. microti *is a 12 kbp insertion (11,742 bp), that is unique to *B. microti *(Figure 1). This insertion is located in chromosome 2 (position 1,038,869 to 1,050,617), between a gene for tRNA_Leu _and gene BMI_II1054, ortholog of BRA1053 in *B. suis *1330 genome. A significant, but partial match to a phage integrase (BMI_II1048) was detected at an extremity of this island (best hit in the genome of *Hyphomonas neptunium*, YP_760435.1, 59% identity). This putative integrase is flanked by an ORF (BMI_II1049) showing similarity with phage excisionases. Altogether, these findings suggest that the 12 kbp genome insertion originates from a yet unidentified phage. Moreover, the target for this insertion was a GGCACCA motif, found both at the end of the tRNA, and identically conserved at the end of the insert. Accordingly, most of the other ORFs within this island exhibit remote similarities with phage ORFs in protein sequence databases. However, these phage-derived genes now appear defective, as the sequence homology only partially cover the ORFs. Interestingly, a predicted gene in this region (BMI_II1051) has a best match against *O. anthropi *(Oan_0220). Finally, this *B. microti *specific genomic island bears no similarity with the 26.5 kbp island recently reported in the genome of *B. ovis *[12]. The origin of this insertion remains unclear since its tentative detection by a recently developed specific PCR [6] with *Brucella *phage DNA (Tb, Wb, F1 and F25) as template was negative. Therefore, it seems unlikely that the insertion has derived from the most commonly known *Brucella *phages.

### Insertion sequences

Insertion sequences of the IS711/IS6501 family are commonly found in *Brucella*, but other IS elements are also present scattered through the genomes. In *B. microti*, we identified 13 copies of IS711 elements, 2 IS2020 elements, 2 IS1953 elements, 2 ISBm1 elements, one of them disrupted by the insertion of an IS711 element, one ISBm2 element and one ISBm3 element. The main difference with respect to *B. suis *1330 is the number of IS711 insertion sequences. IS711 Insertion sequences which are not present in both *B. microti *and *B. suis *1330 are listed in Table 1. Seven are present in *B. microti *only and one in *B. suis *1330 only. Interestingly, those changes are all found on the large chromosome. The insertion site between BMI_I1295 and *cob*L has already been reported *in B. microti *[7], and the insertion site between tRNA_Met _and *omp*28 (BMI_I1490/BR1475) was previously thought to be specific of *Brucella *isolated from marine mammals [13]. One of those insertions impairs a hypothetical protein in *B. suis *1330 (BR0722) and in two other cases, the insertion is located in the 5' upstream-region of a gene (*thr*S/BMI_I1076 and BMI_I1903) but does not seem to disrupt a putative promoter [14]. None of the insertions thus appear to disrupt existing operon structures or inactivate putative promoters.

**Table 1:** Location of IS711 type insertion sequences specific either of *B. microti *or *B. suis *1330.

Genomic context in *B. microti*	Coordinates in *B. microti*	Genomic context in *B. suis *1330	Coordinates in *B. suis *1330
Between *rps*A (BMI_I28/BR0027) and BMI_I31/BR0028	Chr1:33557–34400	Not Present.	Chr1: 33555
Inside gene BR0722 (hypothetical protein)	Chr1: 708832–709676	Not Present.	Chr1: 706394
Between gene BMI_I784/BR0786 and BMI_I787/BR0787	Chr1: 771573–772416	Not Present.	Chr1: 768256
Between genes *thr*S (BMI_I1076/BR1071) and BMI_I1079/BR1072	Chr1: 1047382–1048225	Not Present.	Chr1: 1043121
Between gene BMI_I1295/BR1284 and *cob*L (BMI_I1298/BR1285)	Chr1: 1251779–1252621	No Present.	Chr1: 1245497
Between tRNA-Met and omp28 (BMI_I1490/BR1475)	Chr1: 1436887–1437731	Not Present.	Chr1: 1429443
Between genes BMI_I1899/BR1879 and BMI_I1902/BR1880	Chr1: 1823468–1824311	Not Present.	Chr1: 1815480
Not Present.	Chr1: 1626452	Between genes BR1671/BMI_I1694 and BR1674/BMI_I1695	Chr1: 1618084–1618927

For each insertion sequence, the genomic position of the insertion sequence and of the inserted position in the cognate genome are given.

### Tandem Repeat Analysis

The MLVA-15 typing systems [15], based on multilocus VNTR (Variable Number of Tandem Repeats) and extended later into MLVA-16 [16] used a set of primer pairs (Listed in Table 2, [15]), selected for their variability in a set of three *Brucella *genomes. We searched for these primers in the genome of *B. microti *and reported their positions and the theoretical lengths of the corresponding PCR products in Additional file 4. Of the 80 primer pairs reported in [15], 78 had a perfect match to the *B. microti *genome sequence, and two exhibited one mismatch in one of the primers (Bruce07 and Bruce24). The genomic positions leading to VNTR amplicons of different sizes between *B. microti *and *B. suis *1330, as well as other indels, are listed in Additional file 3. On the 16 MLVA-16 primer pairs, 14 yield PCR fragments of different sizes. This explains the differences in the MLVA profile of *B. microti *with respect to other *Brucella*

**Table 2:** Differentiating genes in the genomes of *B. suis *1330 and *B. microti*.

*B. microti *ID	status in *B. microti*	*B. suis *1330 ID	status in *B. suis *1330	status in *B. ovis *ATCC 25840	status in *B. suis *ATCC 23445	status in *B. abortus *S19	status in *B. melitensis *biovar Abortus 2308	status in *B. abortus *biovar 1 str. 9–941	status in *B. melitensis *16M	status in *B. canis *ATCC 23365	status in *O. anthropi *ATCC 49188	Annotation/comment
Genes impaired in all other *Brucella*												

BMI_I149	+	BR0146	fs	fs	fs	fs	fs	fs	fs	fs	+	BMI_I149 ortholog is pseudogene BR0146, malate dehydrogenase (oxaloacetate-decarboxylating) (NADP(+)), phosphate acetyltransferase
BMI_I1566	+	BR1552	fs	fs	fs	fs	fs	fs	fs	fs	+	BMI_I1566 ortholog is pseudogene BR1552, aspartyl/asparaginyl beta-hydroxylase
BMI_I1599	+	BR1586	* (2)	*, fs	* (1)	* (2)	*(2)	*(2)	* (1)	*(2)	+	BMI_I1599 ortholog is pseudogene BR1586, extracellular solute-binding protein family 5, DppA
BMI_II978	+	BRA0985	*	*	*	*	*	*	*	*	+	BMI_II978 ortholog is pseudogene BRA0985, transcriptional regulator, MarR family

Gene shorter in other *Brucella*												

BMI_I2199	+	BR2178	smaller	smaller	smaller	smaller	smaller	smaller	smaller	smaller	+	BMI_I2199 orthologs in other brucella are smaller, hydrolase

Genes impaired in almost all other *Brucella*												

BMI_I135	+	BR0132	fs	+	fs	fs	fs	fs	fs	fs	+	BMI_I135 ortholog is pseudogene BR0132, ATP- dependent helicase HrpB
BMI_I947	+	BR0949	*	*	*	+	*	*	*	*	+ (diff)	BMI_I947 ortholog is pseudogene BR0949, membrane protein involved in aromatic hydrocarbon degradation
BMI_I1332	+	BR1320	*	fs	+	*	*	*	*	*	+	BMI_I1332 ortholog is pseudogene BR1320, sarcosine dehydrogenase
BMI_II122	+	BRA0122	* (small insert)	+ (small insert)	* (small insert)	Mult. fs (small insert)	Mult. fs (small insert)	fs (small insert)	fs (small insert)	* (small insert)	+ (small insert)	BMI_II122 ortholog is pseudogene BRA0122, flagellar motor switch protein FliG

Glutamate metabolism												

BMI_II334	+	BRA0338	*	+ (diff at the beg.)	+	*(2)	*(2)	*(2)	*	*	NF	BMI_II334 ortholog is pseudogene BRA0338, glutamate decarboxylase beta
BMI_II335	+	BRA0339	fs	fs	fs	+	+	+	+	fs	NF	BMI_II335 ortholog is pseudogene BRA0339, glutamate/gamma-aminobutyrate antiporter
BMI_II1124	fs	BRA1118	+	+	+	+	+	+	+	+	+	pseudogene BMI_II1124 ortholog is BRA1118, N- acetylglucosamine kinase
BMI_II715	+	BRA0722	+	fs	+	+	+	+	+	+	+	BMI_II715 ortholog is pseudogene BRA0722, proline dehydrogenase/delta-1-pyrroline-5-carboxylate dehydrogenase

Membrane proteins												

BMI_I75	+	BR0072	Mult. diffs	Mult. diffs	Mult. diffs	Mult. diffs	Mult. diffs	Mult. diffs	Mult. diffs	Mult. diffs	NF	*B. microti *gene is larger, outer membrane protein, quite variable among brucella
BMI_I1045	+	BR1042	fs	fs	fs	+	+	+	+	fs	+	BMI_I1045 ortholog is pseudogene BR1042, mechanosensitive ion channel family protein
BMI_I1334	+	BR1322	fs	fs	+	+	+	+	+	fs	diff	BMI_I1334 ortholog is pseudogene BR1322, MscS mechanosensitive ion channel
BMI_II170	+	BRA0172/BRA0173	Mult. diffs	Mult. diffs	Mult. diffs	Mult. diffs	Mult. diffs	Mult. diffs	Mult. diffs	Mult. diffs	Mult. diffs	BMI_II170 outer membrane autotransporter, similar to mes:Meso_3532
BMI_II422	+	BRA0425	*	+	*	NF	NF	NF	+	*	+	BMI_II422 ortholog is pseudogene BRA0425, hypothetical protein
BMI_II547	+	BRA0553	premature *	fs	+	NF	NF	NF	+	premature *	NF	BMI_II547 hemagglutinin ortholog is BRA0553, which is shorter than its *B. microti *counterpart.
BMI_I1862	+	BR1846	fs	Diff.	fs	fs	fs	fs	fs	Diff. in the middle	NF	*B. microti *gene BMI_I1862 is longer than BR1846, hypothetical protein

For each gene, we indicate its status in the other *Brucella *and *O. anthropi*. This table only includes the genes that are discussed in the text. A more extensive version of this table is given as Additional file 7.

Abbreviations include: * for internal stop, a number indicates multiple stops, fs for frameshift, + for an intact sequence, Mult. Diffs for multiple difference, Mult. Fs for multiple frameshifts, NF for not found.

### Ribosomal RNA

**Figure 3:**
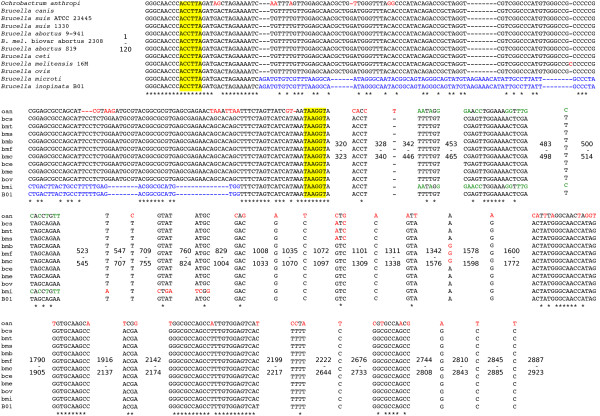
**Alignment of the region around intervening sequence (IVS I) in selected *Brucella *and *O. anthropi***. Fragments of the alignment where all sequences are identical are not shown. The whole alignment with numbering is given in Additional file 5. Sequence fragments shared by *B. microti *and *Brucella *sp. B01 are in blue. Sequence fragments shared by *B. microti *and *O. anthropi *are in green. Other regions are in red. Fragments of the alignment highlighted in yellow correspond to the terminal nucleotides of the secondary structures represented in Figure 4.

**Figure 4:**
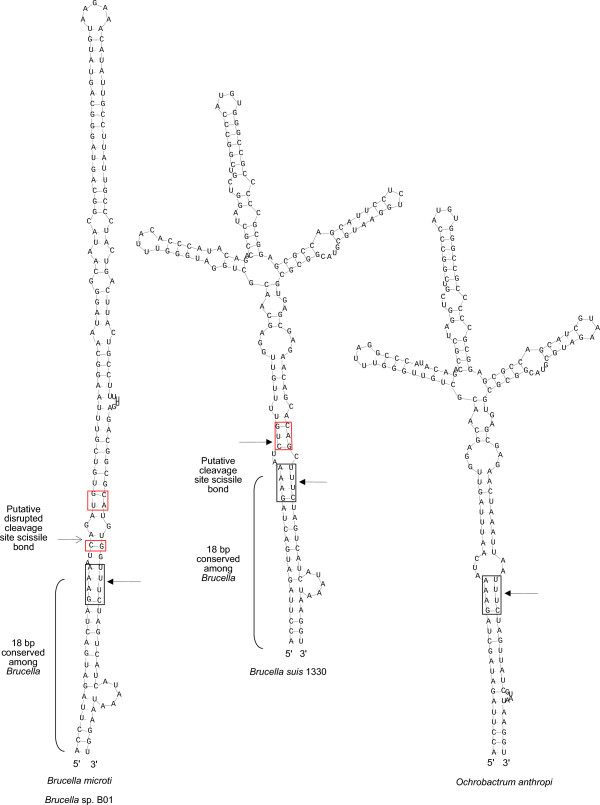
**Predicted secondary structure of the intervening sequence (IVS I)**. The predicted secondary structure of the intervening sequences (IVS I) of the 23S ribosomal RNA in *B. microti *and *Brucella *sp. B01 (left), *B. suis *(middle) and *O. anthropi *(right). Arrows with a dark head represent conserved cleavage sites. Arrows with a thin head represent unconserved cleavage sites. In *O. anthropi*, only the lower part of the cleavage motif is present.

*Brucella *contains three copies of the ribosomal RNA operon. The sequences of 5S and 16S ribosomal RNA genes are nearly identical among all species. In contrast, we noticed that the 23S ribosomal RNA gene sequence of *B. microti *differs markedly from that of the other *Brucella *(Additional file 5), and that the differences map into the intervening sequence (IVS I) localized in the helix 9, cleaved by the RNase III during the maturation of the 23S rRNA. Interestingly, the *B. microti *IVS region differs from that of *O. anthropi*, but is very similar to that of another fast growing *Brucella, B. inopinata *sp. nov. strain B01 [17], whose genome sequence is in the form of 55 contigs on the Patric Web site [18]. Curiously, the similarity between *B. microti *and *B. inopinata *B01 is limited to this particular 23S rRNA region, whereas the rest of their genome sequences are notably different, for instance showing different theoretical PCR fragment size for 2 of the 3 MLVA-16 primer pairs that we were able to locate on the preliminary data (we looked only for exact matches to the primers). Additionally, a tree built from a fragment of *Brucella *genomes (corresponding to the 10,000 first nucleotides of *B. microti *chromosome 1 sequence) showed that *B. inopinata *B01 likely diverged before *B. microti *from the other *Brucella *(Additional file 6). The alignment of the *Brucella *IVS I regions is presented in Figure 3 and an analysis of the secondary structure of the IVS regions in Figure 4. In this figure, the black boxes correspond to previously identified conserved motifs [19]. The nucleotides around the putative cleavage site (red box) are well conserved in all *Brucella*, except for *B. microti *where the CUG consensus cleavage motif is split. In contrast to this IVS I region which is shared between *B. microti *and *B. inopinata *B01, a second region, between positions 446 and 523 in the alignment (Figure 3 and Additional file 5), is perfectly identical between *O. anthropi *and *B. microti*, and distinct from *B. inopinata *B01 which is identical to other *Brucella *in this region.

### Gene content analysis and comparison with other Brucella and Ochrobactrum anthropi

Genes differentially annotated or presenting notable differences between *B. microti *and *B. suis *1330 are listed in Table 2 (for the genes discussed in the text) and Additional file 7 (for a complete version). We reported the status of each of these genes in the other *Brucella *and *O. anthropi, a*s well as the nature of the change at the sequence level that turns the intact gene in *B. microti *or *B. suis *1330 into a pseudogene in the other organisms. In agreement with the observation (Figure 2) that the branch length leading to *B. microti *is shorter than those leading to other *Brucella*, we note (Additional file 7) that the number of pseudogenes in *B. microti *is half that of *B. suis *1330.

### Genes specific to *B. microti *and *O. anthropi *and impaired in all other Brucella

In term of metabolic capabilities, *B. microti *(Table 1 in [7]) was experimentally found much more similar to *O. anthropi *than to other *Brucella*. We thus examined the gene content of *B. microti *to identify genes shared with *O. anthropi *but impaired in all other sequenced *Brucella*. Only 4 such genes were found. Phylogenetic trees for those genes are shown in Additional file 8(A–D).

BMI_I149 has an ortholog in *O. anthropi *(Oant_0158) and is impaired in other *Brucella*. In *B. suis *1330, the corresponding pseudogene is BR0146. Homologues for this gene are found in many *Rhizobiales *such as *Bartonella *or *Mezorhizobium *(Additional file 8A). The enzyme encoded by this gene, malate dehydrogenase (oxaloacetate-decarboxylating) (NADP+) [EC:1.1.1.40], is involved in pyruvate metabolism. The reaction is: (S)- malate + NADP+ = pyruvate + CO2 + NADPH. This gene has a paralog BMI_I1020, the orthologs of which are intact in all *Brucella *and *Ochrobactrum *(Additional file 8A).

BMI_I1566, corresponding to pseudogene BR1552 in *B. suis *1330, is an aspartyl/asparaginyl beta-hydroxylase which is intact in *B. microti *and *O. anthropi *(Oant_1613) and impaired in other *Brucella *(Additional file 8B). The product of this gene [EC:1.14.11.16] catalyzes the reaction: peptide-L-aspartate + 2-oxoglutarate + O2 = peptide-3-hydroxy-L-aspartate + succinate + CO2. The succinate (or succinic acid), product of this reaction, is one of the compounds used in the physiological reactions that differentiate *B. microti *from other *Brucella *[7]. Succinate is involved in many metabolic pathways such as the citrate cycle (TCA cycle).

BMI_I1599, corresponding to pseudogene BR1586 in *B. suis *1330 is intact in *B. microti*, impaired in other *Brucella*, and intact in *O. anthropi *(Oant_1582) (Additional file 8C). This gene is predicted to encode an extracellular solute-binding protein belonging to an ABC-type transport system probably involved in dipeptide transport. However, in the case of *B. microti*, the nearby *dpp*C gene encoding the permease component of this transport system is impaired (pseudogene BMI_I1597), with probable consequences on the whole transport system.

BMI_II978, encoding a transcriptional regulator of the MarR family is present in *B. microti *and *O. anthropi *(Oant_1375) and contains an internal STOP codon in other *Brucella *(Additional file 8D). It corresponds to pseudogene BRA0985 in *B. suis *1330. This family of transcriptional regulators mediates the response to multiple environmental stresses and the resistance to multiple antibiotics.

Finally, the *B. microti *hydrolase BMI_I2199/BR2178, is similar to the one in *O. anthropi*, while its homologues in other *Brucella *species are much shorter, lacking the last 40 amino-acids, which is likely to have functional consequences. Its homologues in *B. canis *and *B. suis *ATCC 23365 are annotated as acetyltransferases, part of the acetoin cleaving system.

### Genes intact in *B. microti *and *O. anthropi *and impaired in almost all other Brucella

Some genes are intact in *B. microti *and *O. anthropi *but are impaired in almost all *Brucella*, with some exceptions. For instance, the ATP-dependent helicase HtrB (BMI_I135), pseudogene BR0132 in *B. suis *1330, presents a frameshift in all *Brucella *except *B. ovis *(BOV_0127) and is intact in *O. anthropi*. This is also the case for BMI_I947 corresponding to pseudogene BR0949 in *B. suis *1330. This gene exhibits an internal STOP codon in all *Brucella *except *B. abortus *S19 (BabS19_I09030) and is present in *O. anthropi *(Oant_2240). This gene encodes a transport protein sometimes annotated as outer membrane protein E. The gene encoding sarcosine dehydrogenase (BMI_I1332, EC:1.5.99.1), corresponding to pseudogene BR1320 in *B. suis *1330, is intact in *B. microti *and *O. anthropi *but impaired in other *Brucella *except *Brucella suis *ATCC 23445. This enzyme catalyzes the following reaction: sarcosine + acceptor + H2O = glycine + formaldehyde + reduced acceptor. The flagellar motor switch gene, *fli*G (BMI_II122, pseudogene BRA0122 in *B. suis 1330*), which is apparently intact in *B. ovis*, *B. microti *and *O. anthropi*, is impaired in other *Brucella*.

### Other potentially significant differences in gene content between *B. microti *and other Brucella

#### Glutamate metabolism

Quite a number of genes involved in glutamate metabolism are missing from certain *Brucella *species.

The gene of glutamate decarboxylase beta (*gad*B, BMI_II334) contains an in-frame STOP codon in *B. suis *1330 and appears to be damaged in many *Brucella *(except for *B. ovis *and *B. suis *ATCC 23445). The product of this gene catalyzes the decarboxylation of glutamate into gamma-aminobutyric acid (GABA) and CO2. The nearby glutamate/gamma-aminobutyrate antiporter (*gad*C, BMI_II335) is also impaired in *B. suis *1330, presenting an internal frameshift as in *B. ovis*, *B. canis *and *B. suis *ATCC 23445. Taken together, these two genes are only intact in *B. microti *(*O. anthropi *does not have any of those genes). These two genes are involved in the generation of a proton motive force in *Lactobacillus *strains [20], but also in acid resistance mechanisms [21-23].

Also involved in glutamate and other amino-acids metabolisms, the *B. suis *1330 gene (BRA1118) encoding a N-acetylglucosamine kinase presents a frameshift and is thus impaired in *B. microti *(BMI_II1124). This enzyme [EC:2.7.1.59] catalyzes the following reaction: ATP + N-acetyl-D-glucosamine = ADP + N-acetyl-D-glucosamine 6-phosphate.

The proline/dehydrogenase/delta-1-pyrroline-5-carboxylate dehydrogenase (BMI_II715) is a pseudogene in *B. suis *1330 (BRA0722). This enzyme [EC:1.5.1.12] catalyzes the reaction: (S)-1-pyrroline-5-carboxylate + NAD(P)+ + 2 H2O = L-glutamate +NAD(P)H + H^+ ^and is involved in glutamate, arginine and proline metabolism.

#### Membrane proteins presenting significant differences between *B. microti *and other Brucella

We also observed a number of changes in genes encoding membrane proteins. The outer membrane protein encoding gene BMI_I75 exhibits large differences in size, being larger in *B. microti *than in its counterparts in other *Brucella*. A small conductance mechanosensitive ion channel protein encoding gene (BMI_I1045) has frameshifts in *B. suis *1330 (pseudogene BR1042), *B. ovis *and *B. canis *and is intact in *B. microti *and *O*. *anthropi*. A second small conductance mechanosensitive ion channel protein encoding gene (BMI_I1334) is impaired in *B. suis *1330 (pseudogene BR1322), *B. canis *and *B. ovis*

An outer membrane autotransporter, encoded by BMI_II170, is paradoxically most similar to the protein Meso_3532 in *Mesorhizobium *sp. BNC1 than to its homologues in all *Brucella *and *Ochrobactrum*.

The *B. microti *membrane protein Bme3, encoded by BMI_II422, corresponds to pseudogene BRA0425 in *B. suis *1330. Genes at this locus have been reported to be involved in polysaccharide synthesis [24].

The ortholog of gene BMI_II547, encoding a cell wall surface hemagglutinin in *B. microti*, presents a premature STOP codon in *B. suis *1330 (BRA0553). Remarkably, a fragment of this gene, position 534037 to 534117 in *B. microti *chromosome 2 sequence, is found multiple times in *Brucella *genomes. Its abundance (blastn search [25], no filter, E-value < 10^-30^) ranges between 38 times in *B. ovis *to 13 times in *B. microti *and *B. suis *ATCC 23445 and 6 to 7 times in the other *Brucella *studied in this work. This repeated element could reveal useful for typing purpose.

*B. microti *gene BMI_I1862 is quite different in length with respect to its counterparts in other *Brucella*. The *B. microti *version has a 126 nucleotides insert. This gene encodes a protein with a Yada-like C-terminal domain characteristic of a family of surface exposed bacterial proteins. *O. anthropi *has no homolog for this gene.

#### Gene clusters encoding the components of the flagellum

All genes involved in flagella assembly and present in *O. anthropi *are apparently intact in *B. microti*, in contrast to the situation in other *Brucella *where at least one gene is impaired (Table 3). However, there is a significant difference in flagella gene organization between *Ochrobactrum *spp. and *Brucella *spp. In *O. anthropi*, the flagella genes are essentially found in two clusters. One contains *flh*B, *fli*G, *fli*N, *fli*M, *mot*A, *flg*F, *fli*I, *flg*B, *flg*C, *fli*E, *flg*G, *flg*A, *flg*I, *flg*H, *fli*L, *fli*P and the other contains *fli*R, *flh*A, *fli*Q, *flg*D, *flb*T, *fla*F, *flg*L, *flg*K, *flg*E, *fli*K, *mot*C, *mot*B, *fli*F, *fli*C. In *Brucella*, a 15 kb insert containing genes not related to flagella interrupts the first group between genes *fli*I and *flg*B (Additional file 9).

**Table 3:** Status of the genes involved in flagella assembly.

**Gene name/*B. microti *ID**	***B. microti***	***B. suis *1330**	***B. suis *ATCC 23445**	***B. ovis *ATCC 2584 0**	***B. canis *ATCC 23365**	***B. melitensis *16M**	***B. abortus *S19**	***B. abortus *biovar 1 str. 9–941**	***B. melitensis *biovar Abortus 2308**	***O. anthropi *ATCC 49188**	
*flh*B/BMI_II121	+	+	+	+	+	+	+	+	+	+	Locus 1 in *O. anthropi*
*fli*G/BMI_II122	+	0	0	+	0	0 a)	0	0	0	+	
*fli*N/BMI_II123	+	+	+	+	+	+	+	+	+	+	
*fli*M/BMI_II125	+	+	+	0	+	+	0	0	0	+	
*mot*A/BMI_II126	+	+	+	+	+	+	+	+	+	+	
*flg*F/BMI_II128	+	+	+	+	+	+	+	+	+	+	
*fli*I/BMI_II129	+	+	+	+	+	+	+	+	+	+	
*flg*B/BMI_II149	+	+	+	+	+	+	+	+	+	+	
*flg*C/BMI_II150	+	+	+	+	+	+	+	+	+	+	
*fli*E/BMI_II151	+	+	+	+	+	+	+	+	+	+	
*flg*G/BMI_II152	+	+	+	+	+	+	+	+	+	+	
*flg*A/BMI_II153	+	+	+	+	+	+	+	+	+	+	
*flg*I/BMI_II154	+	+	+	0	+	+	0	0	0	+	
*flg*H/BMI_II156	+	+	+	+	+	+	+	+	+	+	
*fli*L/BMI_II157	+	+	0	+	+	+	+	+	+	+	
*fli*P/BMI_II158	+	+	+	+	+	+	+	+	+	+	

											

*fli*R/BMI_II1137	+	+	0	+	+	+	+	+	+	+	Locus 2 in *O. anthropi*
*flh*A/BMI_II1138	+	+	+	+	+	+	0	0	0	+	
*fli*Q/BMI_II1139	+	+	+	+	+	+	+	+	+	+	
*flg*D/BMI_II1140	+	+	+	+	+	+	+	+	+	+	
*flb*T/BMI_II1141	+	+	+	+	+	+	+	+	+	+	
*fla*F/BMI_II1142	+	+	+	+	+	+	+	+	+	+	
*flg*L/BMI_II1143	+	+	+	+	+	+	+	+	+	+	
*flg*K/BMI_II1144	+	+	+	+	+	+	+	+	+	+	
*flg*E/BMI_II1145	+	+	+	+	+	+	+	+	+	+	
*fli*K/BMI_II1148	+	+	+	+	+	+	+	+	+	+	
*mot*C/BMI_II1149	+	0	+	+	+	+	+	+	+	+	
*mot*B/BMI_II1150	+	+	+	+	+	+	+	+	+	+	
*fli*F/BMI_II1152	+	+	0	+	+	+	+	+	+	+	
*fli*C/BMI_II1153	+	+	+	+	+	+	+	+	+	+	

Status of the genes involved in flagella assembly in *O. anthropi, B. microti *and 8 *Brucella *(+ gene present, 0 gene absent). The genes are grouped in two loci in *O. anthropi*. The first locus is interrupted by a 15 kb insertion in *Brucella*. a) A gene annotated as *fli*G is present in the genome sequence of *B. melitensis *16M. However, the predicted protein is shorter than it should be and is probably a pseudogene (see Table 1). In *Brucella*, Locus 1 is interrupted by a 15 kbp insert, between *fli*I and *flg*B.

#### Survival in the soil

Paulsen et al. [11] noticed that the *B. suis *operon (BRA0636-BRA0647), encoding an homoprotocatechuate pathway, is widely distributed among diverse soil microorganisms and may contribute to the survival of the bacteria outside of its host. Interestingly, 3 of these genes present a frameshift in *B. microti*, suggesting that they do not significantly contribute to its survival in the soil. In contrast, an other operon cited in the same context (BRA1155-BRA1162) [11], encoding a beta-ketoadipate pathway is intact in both *B. suis *1330 and *B. microti*.

## Discussion

### Voges-Proskauer reaction

In terms of metabolic capabilities, *B. microti *(Table 1 in [7]) was found much more similar to *O. anthropi *than to other *Brucella*. One of the tests for which *B. microti *was positive is the Voges-Proskauer reaction. The Voges-Proskauer reaction demonstrates the possibility for an organism to produce acetoin and 2,3-butanediol and is an established biochemical test for distinguishing wide classes of bacteria. The pathway leading to the production of acetoin is described for instance in [26]. It involves the transformation of pyruvate into alpha-acetolactate by the acetolactate synthase, then the conversion of alpha-acetolactate to acetoin by the alpha-acetolactate decarboxylase followed by the conversion of acetoin to 2,3-butanediol. *Brucella *species possess genes for the acetolactate synthase 3 (BMI_I1399, large subunit and BMI_I1400, small subunit) and the acetolactate synthase 2 (large subunit only BMI_II939). The conversion of acetoin to 2,3-butanediol is performed by the homolog of *als*O (VC1591) in *Vibrio cholera *which is BMI_I1134. A gene apparently missing from this pathway is the homolog of *als*D (VC1589) in *V. cholera*, the alpha-acetolactate decarboxylase. However, as reported for *Bacillus subtilis *[27], this reaction can occur spontaneously at low pH, in absence of *als*D. This suggests that known *Brucella *species have all the enzymes necessary to produce acetoin. Their negative testing for the Voges-Proskauer reaction might thus be due to an indirect cause, such as the lack of a sufficient supply of pyruvate. Like *O. anthropi*, but in contrast with other *Brucella, B. microti *possesses two paralogs of malate dehydrogenase (BMI_I149 and BMI_I1020) which catalyzes a reaction producing pyruvate. It is thus tempting to speculate that this enzyme duplication might be linked to the positivity of the Voges-Proskauer reaction in *B. microti*.

### Proton motive force and acid-resistance mechanism

Of notable interest is the presence of the gene tandem BMI_II334 and BMI_II335 encoding a glutamate decarboxylase beta GadB and a glutamate/gamma-aminobutyrate antiporter GadC, respectively. These proteins might give *B. microti *the potential to generate a proton motive force from the decarboxylation of glutamate. This capacity might have been lost in other *Brucella *where either of these genes are found impaired. In *Lactococcus lactis *[21], *Shigella flexneri *[22] and *Escherichia coli *[23] GadB and GadC were shown to participate in a glutamate-dependent acid resistance mechanism. Acid resistance mechanisms allow enteric pathogens to overcome acid stress in the gastrointestinal tract of their host [28]. In the case of *B. microti*, this system may help the bacteria to survive in acid soils. More importantly, it might also play a role in intracellular survival, as within hosts macrophages, *Brucella *species reside in a low pH environment, where the importance of the *gad*B, *gad*C, *hde*A gene cluster as an acid resistance locus as already been suggested [29,30].

### Motility

Due to their lifestyle, many intracellular bacteria have lost their capacity to produce functional flagella [31]. Despite the presence of numerous flagella genes [32], *Burkholderia mallei *is non-motile whereas *Burkholderia pseudomallei *and *Burkholderia thailendensis *are motile. The lack of motility of *B. mallei was *traced back to a 65 kb insertion within the *fli*P gene as well as a frameshift in the flagellar motor gene *mot*B [32]. Similarly, *Yersinia pseudotuberculosis *and *Yersinia enterocolitica *are motile [33] whereas *Yersinia *pestis KIM is non-motile in spite of the presence of a nearly complete set of flagella genes, but with a truncated gene for transcription factor FlhD. In each of the above cases, the host adapted species are non-motile (*B. mallei*/*Y. pestis*) whereas the others are motile. Although all available *Brucella *genomes possess genes for the flagellum complex, *Brucella *are non-motile and display no flagellum under standard conditions. However, under specific conditions during early exponential growth phase, *B. melitensis *16M has been reported to express some of the key genes of the flagellar apparatus and assemble a sheathed flagellum which is required for virulence in a mouse infection model [34,35].

The motor switch gene *fli*G is the gene most often found impaired in *Brucella *(Table 3), with exceptions in *B. ovis *and *B. microti*. This rotor protein is essential for the assembly and the function of the flagellar motor [36]. In *B. ovis*, *fli*M, encoding the flagellar C ring protein is impaired (as in *B. abortus *S19, *B. abortus *9–941 and *B. melitensis *biovar Abortus 2308), explaining the lack of flagella in *B. ovis*. In contrast with these *Brucella *species, *B. microti *does not present a readily apparent defect among the proteins constituting the flagellar assembly complex. Its lack of a visible flagellum could thus be due to more subtle causes. In this context, we noticed that one of the two flagella gene clusters of *O. anthropi *is split in *Brucella *genomes (Additional file 9). Such a modification might have disrupted the coordinated expression of those flagella genes. However, given the integrity of its individual flagellar genes, *B. microti *might express a sheathed flagellum under specific conditions, as observed for *B. melitensis *16M [34,35].

### The 12 kbp insertion

The specific 12 kbp genomic island of probable phage origin is of obvious interest for identification purpose, and has already been used for the recovery of *B. microti *from soil samples [6]. Blast (tblastn) searches of nucleotide sequences within this island against the Genbank database found best matches in *O. anthropi, Nitrobacter hamburgensis *X14 and other *Rhizobiales*. This suggests that this element originated from a phage commonly infecting *Rhizobiales*. In a recent study [37], evidence of horizontal gene transfers in *Brucella *genomes were reported. Those SARs (Shared Anomalous Regions) consist of regions 2 to 19 kb long, sometimes flanked on one side by a tRNA and on the other side by a fragment of that tRNA, as found in the *B. microti *genomic island. Although some important genes (*e.g*. Type IV secretion or LPS [lipopolysaccharide] synthesis genes) apparently entered the *Brucella *genomes through this mechanism, the *B. microti *island appears devoid of functional genes. Paradoxically, the most striking genome structure difference between *B. microti *and other *Brucella *is probably of no phenotypic consequence.

### 23S ribosomal rna

The cleavage of the 23S rRNA IVSs by RNase III results in a specific fragmentation pattern. In *B. microti*, the 5' fragment is predicted to be 127 bp long, the IVS I (helix 9) 153 bp long and the 3' fragment 2.6 kbp long. The IVS is composed of palindrome sequences and repeated motifs forming stable secondary structures (stem-loop) (Figure 4) [38]. The complementary ends of the IVS are highly conserved between *Brucella *species and correspond to inverted DNA repeats characteristic of mobile genetic elements [38]. In *B. microti*, the 23S maturation leading to the IVS removal may not occur because of sequence variations at the cleavage site. In *Salmonella typhimurium*, RNase III^- ^mutants are viable, suggesting that the removal of the intervening sequence is not required for 23S function [39,40]. The fragmentation of 23S rRNA during post-transcriptional processing of precursor rRNA has been reported for *Brucella *[41], however, no information is available concerning *B. microti*. Concerning the role of the 23S rRNA fragmentation, results in *Salmonella *[42] indicate that the degree of fragmentation correlates with the amount of 23S rRNA degradation in stationary phase, allowing for a post-transcriptional control of ribosome production. Knowing that two fast-growing *Brucella *isolates, *B. microti *and *B. inopinata *B01, share a similar change in their 23S rRNA structure, it is tempting to speculate that this change, impeding IVS removal, could have an impact on their growth rates. It is surprising that two phylogenetically distant species of *Brucella*, as revealed by sequence analysis of VNTR regions and phylogenetic analysis (Additional file 6), have exactly the same IVS sequence. This finding pleads either for an ancestral nature of this IVS or for a recent exchange of 23S rRNA sequences between *B. inopinata *B01 and *B. microti*. However, part of the 23S rRNA gene sequence of *B. microti *was found identical to that of *O. anthropi*, this region not being shared with *B. inopinata *B01. This mosaic structure of the 23S rRNA gene sequence of *B. microti*, partly identical to that of *B. inopinata *B01 and partly identical to that of *O. anthropi *confirms the existence of horizontal gene transfers in *Brucella*.

### Brucella virulence genes

Major virulence factors of *Brucella *that have been characterized include the Type IV secretion system [43,44], LPS [45], Omp25 [46], and the BvrS-BvrR two component system [47]. All of these virulence gene sequences were found to be identical in *B. microti *and *B. suis *1330. Interestingly, two ORFs involved in LPS biosynthesis as well as Omp25 are found in one of the nine genomic islands (GI-2) which were revealed by whole-genome micro-array analysis in *B. melitensis *16M [48,49].

### Phylogeny of the genus Brucella

The newly determined genome sequence of *B. microti *allows us to revisit the phylogeny of the *Brucella *genus. Our results (Figure 2) are in agreement with previous works [37,50] which regroup on one hand *B. suis *1330, *B. suis *ATCC 23445 and *B. canis*, and on the other hand *B. abortus *S19, *B. abortus *9–941 and *B. melitensis *biovar Abortus 2308 together with *B. melitensis *16M. *B. microti *and *B. ovis *separated earlier from those groups. It was claimed [50] that the *B. ovis *lineage was "basal" to the rest of the *Brucella *lineage, dating the divergence of most *Brucella *species from their common ancestor 86,000 to 296,000 years ago. Our analyses now indicate that the *B. microti *lineage is at least as "basal" as *B. ovis*, and anticipating on the completion of its genome sequence, the divergence of *B. inopinata *B01 will probably appear even more ancestral.

## Conclusion

Unexpectedly in the light of its numerous phenotypic peculiarities, *B. microti *was found to have a genome sequence very close to that of previously characterized *Brucella *species. With respect to its closest relative, *B. suis *1330, the genome sequence of *B. microti *was found 99.84% identical in perfectly aligned regions, and no less than 99% identical taking into account insertion-deletions. Although we identified at least 4 genes impaired in all studied *Brucella *but intact in *B. microti *and *O. anthropi*, it is unlikely that these differences alone could explain the numerous *Ochrobactrum*-like phenotypic traits exhibited by *B. microti*, as well as its increased virulence. Additionally, we have identified an unexpected alteration of the 23S rRNA gene sequence of *B. microti*, also shared by an other fast growing novel *Brucella *species *B. inopinata *sp. nov. strain B01. This sequence variation could have a pleiotropic effect by increasing the number of ribosomes per bacterial cell and thus enhance the overall translation activity. Finally, the phenotypic characteristics of *B. microti *might also be due to genome variations in non-coding (regulatory) regions influencing the expression level of numerous genes. Our study appears to be the first encountering a new limitation of the comparative genomic approach in the elucidation of phenotypic traits: usually even close (e.g. virulent *vs*. non virulent) strains display too many differences in their genomes to allow the straightforward identification of the relevant genes. Here, we experienced the opposite problem, being left with too few gene differences to explain a large number of phenotypic variations. A differential analysis of the transcriptome of *B. microti vs*. that of its closest genomic relative *B. suis *1330 as well as complementation studies should help reveal how their quasi-identical gene content could result in two microorganisms exhibiting so many differences in their metabolic behaviors, life-styles, and virulence.

## Methods

### Strain information and Accession Numbers

*Brucella microti *CCM 4915^T ^genome sequence is deposited in the Genbank database under accession numbers CP001578 for the large chromosome and CP001579 for the small chromosome.

### Shotgun sequencing and finishing

An initial shotgun sequencing using GS-FLX produced 414,552 reads of average size 213 bp. Assembly with the Newbler program resulted in 90 contigs above 500 bp. Newbler assembler contigs were converted into artificial Sanger reads. Based on the extensive similarity between the genomes of *Brucella*, we were able to determine tentative primer pairs that were tested by PCR and then used for sequencing by Sanger technology, allowing us to bridge the gaps between contigs. The resulting sequences were subsequently added to the assembly using the Phred/Phrap/Consed software packages [51-53].

### Genomic comparisons

The genomic sequences reported in this article were compared to the available genomic sequences of *B. melitensis *16M [NC_003317 (large chromosome) and NC_003318 (small)] [9], *B. suis *1330 [NC_004310 (large) and NC_004311 (small)] [11], *B. abortus *9–941 [NC_006932 (large) and NC_006933 (small)] [10], *B. melitensis *biovar Abortus 2308 [NC_007618 (large) and NC_007624 (small)]*, B. ovis *[NC_009505 (large) and NC_009504(small)] [12], *B. suis *ATCC 23445 [NC_010169 (large) and NC_010167 (small)]*, B. canis *ATCC 23365 [NC_010103 (large) and NC_010104 (small)] and *Brucella abortus *S19 [NC_010742 (large) and NC_010740 (small)]. In addition we used the genomic sequence of *Ochrobactrum anthropi A*TCC 49188 [NC_009671 (93,589 bp), NC_009672 (57,138 bp), NC_009668 (1,895,911 bp), NC_009667 (2,887,297 bp), NC_009670 (101,491 bp), NC_009669 (170,351 bp)]. Dotplots of the genome sequences were performed using programs from the MUMMER package [54].

### Alignment of genomic sequences

Alignment of chromosomes of *B. suis *1330 and *B. microti *were performed using software from the LAGAN Toolkit [55]. An in-house program to superimpose annotation on the alignment was used to have a finer view of the position of the differences with respect to the annotated genomes, and to aid in the annotation of *B. microti*. The alignment files are available as Additional Files 1 and 2. A list of indels computed from those alignments is presented as Additional file 3.

### Genome annotation

Due to the close similarity between the genomes of *B. suis *1330 and *B. microti*, a large use was made of the full alignment of sequences that is presented in Additional Files 1 and 2 as well as similarity searches against the complete genomes of other *Brucella*. Insertion sequences that were not present in the genome of *B. suis *1330 were identified with the help of the Biotoul IS-Finder [56,57].

### Identification of candidate pseudogenes

Candidate pseudogenes either in *B. microti *or *B. suis *1330 were identified as follows. First, the most similar and intact version of the homologous ORF was selected among one of the available *Brucella *genomes. This sequence was then used to query (using tblastn [25], no filter, E-value < 10^-50^) the remaining *Brucella *and *Ochrobactrum *genomes to detect the eventual absence or disruption of the homologous gene (premature STOP codon, frameshift). 170 cases of premature terminations or frameshifts were found and are listed in Additional file 7. Fifty of them correspond to ORFs altered in *B. microti *and intact in at least one other *Brucella*, while 120 correspond to genes presumably functional in *B. microti *and altered in its closest relative *B. suis *1330.

### Brucella phylogeny

The proteins of the *B. microti *chromosomes were grouped with those of 8 other *Brucella *and *O. anthropi *in a file containing 33,053 proteins. A blastp [25] search (Evalue < 10^-5^) of this set against itself yielded a table of blast results which was used to cluster proteins using a Markov chain clustering algorithm [58]. We selected clusters containing a single protein from each of the initial organism. This procedure resulted in 1,486 clusters of genes present in each *Brucella *and in *O. anthropi*. The 1,486 *Brucella *core proteins were first aligned individually using MUSCLE (v3.7)[59]. Poorly aligned regions were discarded by GBLOCKS (v0.91b) [60] using the Phylogeny.fr platform [61]. The resulting alignments were used as a guide to align the corresponding DNA sequences on a codon basis. After cleaning up the nucleotide alignments for poorly aligned regions, the 1,486 multiple alignments were concatenated in a single alignment of 431,655 codons. The phylogenetic tree was reconstructed using the maximum likelihood method implemented in the PhyML program (v3.0 aLRT) [62]. The default nucleotide substitution model (HKY85) was selected assuming an estimated proportion of invariant sites and 4 gamma-distributed rate categories to account for rate heterogeneity across sites. The gamma shape parameter was estimated directly from the data as well as the transition/transversion ratio. Reliability for internal branches was assessed using the aLRT test (SH-Like). Graphical representation and edition of the phylogenetic tree were performed with TreeDyn (v198.3) [63] and MEGA3 [64].

### Phylogeny of 4 genes unique to *B. microti *and *O. anthropi*

Only 4 genes common to *B. microti *and *O. anthropi *but impaired in all other *Brucella *were identified. These genes are a priori the most likely to contribute to the *Ochrobactrum*-like phenotypic traits of *B. microti*. The corresponding protein sequences were searched against the nr database with the blastp program (E-value < 10^-5^) using the Phylogeny.fr platform [61]. The homologous sequences were aligned with MUSCLE (v3.7) configured for highest accuracy (MUSCLE with default settings). After alignment, ambiguous regions (i.e. containing gaps and/or poorly aligned) were removed with GBLOCKS (v0.91b) using the following parameters: minimum length of a block after gap cleaning of 10, no gap positions were allowed in the final alignment, all segments with contiguous non-conserved positions bigger than 8 were rejected and minimum number of sequences for a flank position: 85%. The phylogenetic tree was reconstructed using the maximum likelihood method implemented in the PhyML program (v3.0 aLRT). The default substitution model (WAG) was selected assuming an estimated proportion of invariant sites and 4 gamma-distributed rate categories to account for rate heterogeneity across sites. The gamma shape parameter was estimated directly from the data. Reliability for internal branch was assessed using the aLRT test (SH-Like). Graphical representation and edition of the phylogenetic tree were performed with TreeDyn (v198.3).

## Competing interests

The authors declare that they have no competing interests.

## Authors' contributions

HCS designed the project, performed the experiments and wrote the manuscript. SA, ML and JMC analyzed the data and wrote the manuscript.

## Supplementary Material

Additional file 1 **Supplementary File 1: Whole genome alignment with superimposed annotation of the large chromosomes of *B. microti *and *B. suis *1330.** Letter x in the consensus line denotes an indel or a point mutation. In protein coding regions, the reading frame is indicated. Click here for file

Additional file 2 **Supplementary File 2: Whole genome alignment with superimposed annotation of the small chromosomes of *B. microti *and *B. suis *1330.** Letter x in the consensus line denotes an indel or a point mutation. In protein coding regions, the reading frame is indicated. Click here for file

Additional file 3 **Supplementary Table 1: List of indels between the genomes of *B. microti *and *B. suis *1330.** The coordinates in *B. microti *and *B. suis *are given as well as the localization and the putative effect of the indels on gene products. Click here for file

Additional file 4 **Supplementary Table 2: Distinctive VNTR typing of *B. microti *as confirmed by the genome sequence.** For each tandem repeat we listed the primer pair, the theoretical length of the amplimer in *B. suis *1330, *B. melitensis *16M, *B. abortus *9–941 and *B. microti *CCM 4915, and the location of the theoretical PCR product in the genome of *B. microti*. Click here for file

Additional file 5 **Supplementary Figure 2: Alignment of the 23S ribosomal RNA gene sequences in *O. anthropi *and other *Brucella *studied in this work.** In addition, we included the sequences of *B. ceti *and *Brucella inopinata *B01. Abbreviations: oan, *O. anthropi; *bcs, *B. canis; *bmt, *B. suis *ATCC 23445; bms, *B. suis *1330; bmb, *B. abortus *9–941; bmf, *B. melitensis *biovar abortus 2308; bmc, *B. abortus *S19; bce, *B. ceti; *bme, *B. melitensis *16M; bov, *B. ovis; *bmi, *B. microti *and B01, *Brucella inopinata *B01. Click here for file

Additional file 6 **Supplementary Figure 1: Phylogenetic representation of the alignment of the regions corresponding to the first 10,000 nucleotides of *B. microti *genome sequence, showing that *Brucella inopinata *sp. nov. strain B01 diverged earlier than the other *Brucella *studied in this work.** The sequence of *B. ceti *is also included. *B. ceti *and *Brucella inopinata *B01 sequences were obtained from the PATRIC web site [18]. Click here for file

Additional file 7 **Supplementary Table 3: Extended list of orthologous genes exhibiting annotation differences between *B. microti *and *B. suis *1330 (one, or both being annotated as pseudogene, or presenting notable difference).** For each gene, we give its identification in *B. microti *and *B. suis *1330 as well as its status in other *Brucella*. Abbreviations include: * for internal stop, a number indicates multiple stops, fs for frameshift, + for an intact sequence, Mult. Diffs for multiple difference, Mult. Fs for multiple frameshifts, NF for not found. Click here for file

Additional file 8 **Supplementary Figure 4: Phylogenetic tree for the 4 genes conserved in *B. microti *and *O. anthropi *and impaired in the other *Brucella*.** The trees were built using the Phylogeny.fr Web Server [65] using defaults settings. A) BMI_I149, malate dehydrogenase (oxaloacetate-decarboxylating) (NADP+) and its paralog BMI_I1020 intact in other *Brucella*; B) BMI_I1566, aspartyl/asparaginyl beta-hydroxylase; C) BMI_I1599, extracellular solute-binding protein belonging to an ABC-type transport system involved probably in dipeptide transport and D) BMI_II978, MarR family transcriptional regulator. Click here for file

Additional file 9 **Supplementary Figure 3: Genomic representation of the region around the cluster of flagella assembly genes that is contiguous in *O. anthropi *and interrupted in *Brucella*.** Intact genes are represented as black arrows, pseudogenes as red arrows. Click here for file
