# Supplementary material for: Brucella microti: the genome sequence of an emerging pathogen
Source: BMC Genomics. 2009 Aug 4;10:352. doi: 10.1186/1471-2164-10-352 (PMC2743711; doi:10.1186/1471-2164-10-352)
Supplement: Additional file 3 — Supplementary Table 1: List of indels between the genomes of B. microti and B. suis 1330. The coordinates in B. microti and B. suis are given as well as the localization and the putative effect of the indels on gene products. [file 1471-2164-10-352-S3.pdf]

Insertion-deletions in Chromosome 1

Coordinates				Insertion *B. microti*	Insertion *B. suis*	Description
B. microti		*B. suis 1330*				
18568	18568	18567		T		mvaB, BMI_I17, pseudogene BR0017, frameshift in B. suis
33557	34400	33555		GGCGTGTCTGCATTTAACGTAACCAGATCATAGCGCATGCGAGATGGACGAAACCCATGAATGCGGTCAATGTTTTCTCGCATCGCAGCGCAATACGACGATAGCGTTTCAACTTGTTAAAAAAGCATTCAATCTGATGGCGTTCCTTGTACAGCCTCCAGTCGATTGTTGGGACACTGGAACGTGTTGGATTGACCTTGATCTGAGCCGTTGCCTTGAGATTGCTGGCAATGAAGGCCCTTAAGTGATCGGCATCATAGGCTGCATCAGCAATGACATGCCCCACACCCTTCAAGCCGGATAGAAGGCTTGAAGCTTGCGGACAGTCACCATAATGGCCGGGTGTTGGCTTTATTCGCAGCGGTAGGCCGATAGCATCGACAACAGCATGCAGCTTGGTCGTCAATCCACCGCGCGAGCGACCGATGCAGGCAGCTTCAGCCCCCCTTTTGCGCCCGCCGCATCTGCGTGGACTTTCGATATGGTGCTATCAATGAGGACATATTCAAAGTCCGGCGTATCAGCCAGGGCATGGAAAAGCCTTTCCCATACACCGGCGTGCGACCAGCGCCGAAAGCGGGCATGAACCGCTGTCCATTTGCCGAAGGTCGCAGGCAGATCGCGCCAGTGCGCTGCATTGGCAGCCATCCACAAGATGGCGTCGACAAATAATCGGTTATCGACGCCACTGCGGCCGGGCGTACCAACTCGCCCCGGAAGATATGCTTCGATCCGGTTCCATTGCTCATCTGTAAGGCTTCGTCTGCTCACGGCTGTTCTCCTTCAACAACCTTGAATCAGAATTTCATGCAAAAGGGAATCCTTGAATGCAGACAAGCCCTAG		IS711 insertion sequence
39461		38617	38617		A	frameshift in gene BMI_I36 corresponding to BR0033, hypothetical protein
41656	41656	40811		A		before gene BMI_I38/BR0035, hypothetical protein
63712	63783	62866		CTGCCCTACTGCCCTACTGCCCTACTGCCCTACTGCCCTACTGCCCTACTGCCCTACTGCCCTACTGCCCTA		TACTGCCC repeat, just 3'end of gene BMI_I59/BR0056 (glycolase family protein). Bruce24*
65660		64744	64775		TGCCCTACTGCCCTACTGCCCTACTGCCCTAC	TACTGCCC repeat, in intergenic region between BMI_I60/BR0057 and BMI_I61/BR0058, Bruce01*
72903		72019	72022		GTTT	56 nt before hemolysinIII family protein, BMI_I71/BR0068
79609	79609	78727		T		intergenic region
79794	79794	78911		T		small difference at the beginning of outer membrane protein BMI_I75/BR0072 (changes slightly the beg. of the protein)
80911	81588	80027		GAGACAGCTTTTCGCTGTCTCGGAACAGGCCGCGTCAGGCTGGAGCCTGACGGTGAACGGAATGGATAAAAGCAGGGTCGCACCGGGCGACATGGTTGATCTGAGCAATAGCGATGGCAATCTCGTTCTTAGCAAGCATGGCACTGGCGTTACGTTTAACCTTGCACCAGATTTGAAAGTAACGAGCCTCGTAGCCGGTAACACCTTTCTGGATACGAACGGACTTGTGATTACCGGCGGTCCGAGCATGACAGTGTCTGGAATTGACGCGGGTCATCTGAATATCCGCCATGTTGCGGATGGAGCTGTGACAGCGACCTCTACGGATGCTGTCAATGGGAGACAGCTTTTCGCTGTCTCGGAACAGGCCGCGTCAGGCTGGAGCCTGACGGTGAACGGAATGGATAAAAGCAGGGTCGGACCGGGCGACACGGTTGATCTGAGCAATAGCGATGGCAATCTCGTTCTTAGCAAGAAGGGCAAGGACGTTACGTTTAATCTTGCATCAGATTTGAAAGTAACCAGCCTTGTAGCCGGTAACACCTTTCTGGATACGAACGGACTTGTGATTACCGGCGGTCCGAGCATGACAGTGTCTGGCATCGACGCGGGTCAACTGAAAATCAGCCATGTTGCGGATGGAGCAGTAACAGTAACCTCGACGGATGCTGTCAACGG		large insert inside outer membrane protein BMI_I75/BR0072. Bruce02*
88647	88662	87085		CTACTGCCCTACTGCC		TACTGCCC repeat, end of gene BMI_I84/BR0081. Bruce25*
110240		108664	108664		T	intergenic region between BMI_I108/BR0105 and BMI_I109/BR0106
110281	110281	108704		T		
137522		135946	135948		TGC	one codon deletion in DNA polymerase I (polA,BMI_I126/BR0123)
144047	144047	142472		A		one nt insertion before gene DNA gyrase subunit B (gyrB,BMI_I128/BR0125)
149660	149661	148084		CG		Gene hrpB, ATP-dependant helicase BMI_I135 is pseudogene BR0132 in B. suis
154475	154475	152897		A		1 nt insertion just before gene BMI_I142/BR0139, lipoprotein
162875	162895	161296		CGGCTCGACAGGCGCGCATTG		In BMI_I149 which corresponds to pseudogene BR0146 in B. suis, malate deshydrogenase
163290	163290	161690		A		
205157		203558	203558		T	Gene BR0192 is pseudogene BMI_I195 , hypothetical protein
210265	210265	208665		T		before gene BMI_I200/BR0196
210712	210712	209111		T		in BMI_I201/BR0197, pseudogene in B. suis and B. microti, transcriptional regulator, LuxR family
215023		213423	213423		A	BMI_I205/BR0201, transcriptionnal regulator, Fis family, pseudogene in B. suis
216781	216838	215180		GGCAAAAAGCATGTCGCGGAAAAGTGGGAACCGGTTTTCCGATCACGACATGCGTATA		in intergenic region between BMI_I206/BR0202 and BMI_I207/BR0203
219176	219176	217517		C		BMI_I209/BR0205, transcriptionnal regulator, LacI family, pseudogene in B. suis
221345	221538	219685		TCGGTCGAAAAGCGTTGCAGCTTGCGAAAGGCGCTTGCCGCCATGTCGCTCATGATTTTCAAGGTGAAGTCGGTCAGGACATAAAACGTGACCTGCCGCGAAACGAGCGCCAGCGCACCCAGCGAGAGCAGGGCGATGAGGGCGTGGATCGCCGCATCCCATGCATTGCTGCCCAGCGACAGCGAATCCACCAG		in BMI_I210/BR0206, ABC transporter, HlyB/MsbA family, pseudogene in B. suis
260361	260361	258507		G		between BMI_I249/BR0246 and tRNA Phe
266983	266983	265128		G		between BMI_I259/BR0255 and tRNA Thr
269733		267879	267889		AGTTCGCGCTC	deletion in B. suis gene BR0259, increases the size of ORF BMI_I263 in B. microti
269903	269903	268058		A		BMI_I264, which has no annotated counterpart in B. suis, hypothetical protein
269908	269908	268062		C		
270044		268199	268201		GGC	
270055	270056	268211		AT		
270095		268251	268260		CATGCCTAAT	
270112	270121	268276		TCAGCGTATC		
270142	270143	268296		CA		
270156		268310	268318		GAGCTAACC	
270162		268325	268328		AGAA	
270168		268335	268346		CGGTGGTCTTAT	
270179		268358	268404		GCTCTGGCCTTGGTCTGCCTTGAAGTCCATTTAGGGCTTGGGGGTGT	
270190		268416	268416		C	
270204		268431	268438		AGTCCAAT	
270216	270219	268449		AGGG		In region corresponding to recombinase BMI_I265/BR0260
270230	270234	268459		TATAC		
270246		268472	268474		CGA	
270280	270283	268507		ACCC		
270292		268517	268522		CAATGC	
270299		268530	268559		GCCAACGTCGCCGCTACCTACCGGGGTCAG	
270302		268564	268568		TGGGG	
270335		268602	268603		GG	
270343	270345	268610		AGC		
270357		268623	268629		GACCTTC	
270370	270386	268641		TCGTCATTGACGAAGTC		
270394	270400	268648		ATCAAGG		
270430		268679	268684		TAGTAA	
270521		268771	268775		TTGCT	
270513	270517	268766		AACTA		
270588	270601	268841		ATGACCTTTGACGG		
270616	270626	268855		CCATGCAGCAA		
270654	270656	268882		ATG		
270691		268918	268921		TGCT	
270729		268960	268968		GCGGCAAGT	
270741		268981	269042		CACCCACAGCACAGCGTCAGGCAGACGAGATTATCCGCTTGAAGGACGAAGGGAAGACCGTC	
270754		269056	269111		CGGCAACCAAGGCGGGGCGGTCTAGTGTATTCAGCATTCTTAAGGAGGCAGGAAGG	

273035		271393	271396		GAAC	intergenic region between BMI_I266/BR0261 and BMI_I267/BR0262
273564		271926	271926		G	intergenic region between BMI_I267/BR0262 and BMI_I268/BR0263
273598	273598	271959		A		
275152	275152	273512		A		intergenic region between BMI_I270/BR0265 and BMI_I271/BR0266
279638	279664	277997		CGGGCACCATGGACACGACCATGACCA		at beginning of BMI_I277/BR0272, urease accessory protein UreF. Bruce26*
314221	314221	312553		T		gene BMI_I306/BR0300 contains a frameshift, hypothetical protein
323493		321826	321826		G	BMI_I316/BR0310, DNA damage inducible protein F, pseudogene in B. microti
350689		349023	349023		A	BMI_I345/BR0340, invasion protein B, pseudogene in B. suis
351589		349924	349924		C	BMI_I346/BR0341, pseudogene both in B. suis and B. microti
352174		350511	350511		C	
359334		357672	357672		C	intergenic region, between BMI_I352/BR0347 and BMI_I353/BR0348
362363	362374	360700		TTTCCAACACGG		in frame insertion in xanthine dehydrogenase. BMI_I355/ BR0350
366091	366099	364416		ATCGCTTCG		in frame insertion in BMI_I358/BR0353
380619		378937	378975		TGCGGCTATCCATGAGGTCGGCAGCCTTGTTCAACAGTG	In frame change in BMI_I372/BR0367, aldehyde dehydrogenase family protein
385019	385123	383374		ATTCTGGAAGCGGCGGGCGTGGCGGCAAGTGTCGTGATGGCGC CGCCACTCGGGGCTCTTGTCGGCGGTGCGCTTCTGGGTGTGAC CTTCATGGCGCTCACCGCT		BMI_I376/BR0371 is pseudogene in B. suis, transporter MFS superfamily
391746	391806	389996		GTTTTGCGTTGGATAATGCAACCGGCAAAAGTGTGCATTTTCG AGCCAAAAGTGCGTAGCG		intergenic region between BMI_I376/BR0376 and BMI_I377/BR0377 (bacA)
410289	410514	408478		AGAAATCCCCGCTCTGCCGTCCGCCCGCAGCGAAGCGAGGACG GACAAATTGAATAATGTGCATGCGGAAGGCGTACGCTGATGCC TGATTTGTTGCTCGAACTCTTTTCCGAAGAAATCCCCGCCCTG CCGTCCGCCCGCAGCGAAGCGAGGACGGACAAACTGAATAATG TGCATGCGGAAGGCGTACGCTGATGCCTGATTTGTTGCTCGAA CTCTTTTCCGA		Difference overlapping beginning of glycyl-tRNA synthetase, beta subunit glyS (BMI_408/BR0404)
416710		414675	414689		CGGTGCGCACCGGCC	BMI_I417/BR0413 is pseudogene in B. suis, GGDEF domain protein
427360	427369	425338		GGTGCCGCCG		BMI_I425/BR0421, phosphoglycerate mutase family protein, is pseudogene in B. suis
430014		427983	427983		A	between gene BMI_I430/BR0427 and aroC (BMI_I431/ BR0428)
435832	435832	433800		T		between genes BMI_I437/BR0434 and BMI_I438/BR0435
440960	440975	438927		GCCCTACTGCCCTACT		TACTGCCC repeat, in intergenic region between BMI_I441/BR0437 and BMI!_442/BR0438. Bruce04*
456820	456820	454771		A		BMI_I455/BR0453 is pseudogene in B. microti, hypothetical protein
463556		461508	461508		A	between genes BMI_I464/BR0462 and BMI_I465/BR0463
479789	479804	477740		CTGCCCTACTGCCCTA		TACTGCCC repeat, between BMI_I482/BR0480 and BMI_I483/BR0481. Bruce30*
486419	486478	484354		GTTTTGCGTTGGATAATGCGACCAGGAAAGGTTTGCATTTCGA GCCAAAAGTGCGAAGCG		insert in BMI_I487/BR0485, hypothetical protein
506470	506470	504345		G		difference in gene BMI_I504/BR0503, hypothetical protein
508028	508029	505902		GC		BMI_I506/BR0505 is pseudogene in B. microti, metallo-beta-lactames family protein
518594		516468	516469		GG	small difference at the end of Insertion sequence Isbm2
526964		524840	524840		T	small difference in IS711 family insertion sequence
526988	526988	524863		T		
530141		528017	528017		A	in transposase region
531793		529670	529670		G	
553612		551490	551490		C	BMI_I554/BR0555 is pseudogene in B. microti, L-asparaginase II protein
557458	557458	555335		C		BMI_I559/BR0560, is longer in B. suis due to this frameshift
569931	570023	567807		CGGTGGCTCATCGTCGGGTGCCGCCGTTTCGGTTGCCGAAGGC ACGAGCGAGATCGCCATCAGTTCGGATACTGGCGGTTCGGTGC GCATTCC		BMI_I573/BR0573, gene is longer in B. microti
571782	571796	569565		TTGGGCCTTGTTTTC		insert in BMI_I575/BR0575. Bruce33*
585699		583469	583469		C	change in the beginning of gene BMI_I589/BR0590
587992	587992	585761		C		BMI_I595/BR0596 is pseudogene in B. suis
591875	591882	589643		CTGGCGGG		BMI_I600/BR0601 is pseudogene in B. suis, Phage Host Specificity Protein
613912	613913	611672		CT		between BMI_I615/BR0616 and BMI_I616/BR0617
623608	623615	621366		GGAATAGG		between BMI_I621/BR0622 and BMI_I622/BR0623, Bruce05*
628743		626495	626495		G	between BMI_I631/BR0632 and BMI_I632/BR0633
637001		634754	634754		G	between BMI_I641/BR0641 and BMI_I642/BR0642
641796		639550	639703		TTTCGGATAAGATGCGCGCAAGGAAAGATCTGCATCCCGAA AAGTGCGAAGCGCCTACGCAGGGAAATCAGTCCACTGGACT GATTTCTGATCCTGCTTCGATCAGATAAGATGCGCGTAGGG CAGATCTGTATCCCGAAAAGTGCGAAGCGGT	between BMI_I646/BR0647 and BMI_I647/BR0648
651677	651733	649583		AGCCGCGATGGACTGGTCATCGAACTGATTCTGCGTGATCTCC ACACCCTGCCGGAA		in frame change in BMI_I656/BR0657
665921	665921	663770		T		between BMI_I674/BR0677 and BMI_I675/BR0679
665928	666194	663776		ATGGGCAGCAAACGTTACCCGCCCGCCCGCAGCCGCGAAGCGA GCGAGGACGGGCAAAGGCAAAAGCGGGCGCTTGCCTGGGAGGG CAATGATGGCACGCGAGCGCATTTATATCTACGACACAACCTC GGTGGATGGGCAGCAGACGTTACCCGCCCGCCCGCAGCCGCGA AGCGAGCGAGGACGGGCAAAGGCAAAAGCGGGCGTTTGCCTGG GAGGGCAATAATGGCACGCGAGCGCATTTATATTTACGATACG ACCCTGCGG		at the beginning of BMI_I675/BR0679, 2-isopropylmalate synthase. Bruce06*
674051	674067	671632		TTTCTCTTAGCCAGCCC		between BMI_I680/BR0684 and BMI_I681/BR0685
685860	685862	683424		CCC		between BMI_I690/BR0695 and BMI_I691/BR0696
704865		702428	702428		C	gene BMI_I712/BR0717 is shorter in B. microti, oxidoreductase, short-chain dehydrogenase/reductase family protein
708832	709676	706394		GGCGTGTCTGCATTTAACGTAACCAGATCATAGCGCATGCGAG ATGGACGAAACCCATGAATGCGGTCAATGTTTTCTCGCATCGC AGCGCAATACGACGATAGCGTTTCAACTTGTTAAAAAAGCATT CAATCTGATGGCGTTCCTTGTACAGCCTCCAGTCGATTGTTGG GACACTGGAACGTGTTGGATTGGCCTTGGATCTGAGCCGTTGC CTTGAGATCGCTGGCAATGAAGGCCCTTAAGTGATCGGCATCA TGGGCCGCGTCAGCAATGACATGTCCCACACCCTTTAAGCCGG ATAGAAGGCTTGAAGCTTGCGGACAGTCACCATAATGGCCGGG TGTTGGCTTTATTCGCAGCGGTAGGCCGATAGCATCGACAACA GCATGCAGCTTGGTCGTCAATCCACCGCGCGAGCGACCGATGC AGGCAGCTTCAGCCCCCCTTTTGCGCCCGCCGCATCTGCGTGG ACTTTCGATATGGTGCTATCAATGAGGACATATTCAAAGTCCG GCGTATCAGCCAGGGCATGGAAAAGCCTTTCCCATACACCGGC GTGCGACCAGCGCCGAAAGCGGGCATGAACCGCTGTCCATTTG CCGAAGGTCGCAGGCAGATCGCGCCAGTGCGCTGCATTGGCAG CCATCCACAAGATGGCGTCGACAAATAATCGGTTATCGACGCC ACTGCGGCCGGGCGTACCAACTCGCCCCGGAAGATATGCTTCG ATCCGGTTCCATTGCTCATCTGTAAGGCTTCGTCTGCTCACGG CTGTTCTCCTTCAACAACCTTGAATCAGAATTTCATGCAAAAG GGAATCCTTGAATGCAGACAAGCCCTAG		IS711 insertion sequence
721916	721916	718633		A		BMI_I731/BR0733 is pseudogene in B. microti, hypothetical protein
739604		736322	736329		AATAGGGT	between BMI_I747/BR0749 and BMI_I748/BR0750
739697	739704	736421		CTCCCTTA		BMI_I748/BR0750, slight change at the end of gene, ribonuclease D
742952	742969	739668		CCGGGCTTTTTGACTTTA		between BMI_I749/BR0751 and BMI_I750/BR0752
755515	755529	752213		CCAATATTGAAATTG		just before BMI_I763/BR0766, hypothetical protein. Bruce36*

771573	772416	768256		GGCGTGTCTGCATTTAACGTAACCAGATCATAGCGCATGCGAGATGGACGAAACCCATGAATGCGGTCAATGTTTTCTCGCATCGCAGCGCAATACGACGATAGCGTTTCAACTTGTTAAAAAAGCATTCAATCTGATGGCGTTCCTTGTACAGCCTCCAGTCGATTGTTGGGACACTGGAACGTGTTGGATTGACCTTGATCTGAGCCGTTGCCTTGAGATTGCTGGCAATGAAGGCCCTTAAGTGATCGGCATCATAGGCTGCATCAGCAATGACATGCCCCACACCCTTCAAGCCGGATAGAAGGCTTGAAGCTTGCGGACAGTCACCATAATGGCCGGGTGTTGGCTTTATTCGCAGCGGTAGGCCGATAGCATCGACAACAGCATGCAGCTTGGTCGTCAATCCACCGCGCGAGCGACCGATGCAGGCAGCTTCAGCCCCCCTTTTGCGCCCGCCGCATCTGCGTGGACTTTCGATATGGTGCTATCAATGAGGACATATTCAAAGTCCGGCGTATCAGCCAGGGCATGGAAAAGCCTTTCCCATACACCGGCGTGCGACCAGCGCCGAAAGCGGGCATGAACCGCTGTCCATTTGCCGAAGGTCGCAGGCAGATCGCGCCAGTGCGCTGCATTGGCAGCCATCCACAAGATGGCGTCGACAAATAATCGGTTATCAACGCCACTGCGGCCGGGCGTACCAACTCGCCCCGGAAGATATGCTTCGATCCGGTTCCATTGCTCATCTGTAAGGCTTCGTCTGCTCAAGGCTGTTCTCCTTCAACAACCTTGAATCAGAATTTCATGCAAAAGGGAATCCTTGAATGCAGACAAGCCCTAG		IS711 insertion sequence
779225		775066	775066		G	BMI_I794/BR0794 is pseudogene in B. microti, hypothetical protein
779961	779962	775801		GG		slight change at the beginning of BMI_I795/BR0795
810878	810878	806716		A		between BMI_I827/BR0827 and BMI_I828/BR0828
840701		836540	836540		A	BMI_I859/BR0862 is pseudogene in B. suis, bacterial extracellular solute-binding protein, family 3
854801	854836	850639		AACAGAATGATTGGTCAGAACAGAATGACTGGCCAG		in frame change in BMI_I875/BR0878, antifreeze protein, type I, Bruce08*
857368		853172	853172		T	BMI_I876/BR0879 is pseudogene in B. microti, beta-hexosaminidase
858243	858248	854046		ACGAGG		
881222		877021	877021		G	between BMI_I904/BR0905 and BMI_I905/BR0906
919341	919341	915139		G		BMI_I941/BR0943 is shorter in B. microti, hypothetical protein
938680	938755	934476		CAGACGCAGACGCAACCGGCACAACCCCAGACCGCTGCTCCTGCATCCAATGCCCCCAAAGCTGTATCCGCGCCGC		between BMI_I959/BR0961 and BMI_I960/BR0962
949697		945419	945419		A	BMI_I980/BR0979 is pseudogene in B. microti, hypothetical protein
980809	980809	976530		A		BMI_I1015/BR1013 is slightly different at the end, frameshift in B. suis BR1013, B. microti ortholog is normal. phospho-2-dehydro-3-deoxyheptonate aldolase
997212		992934	992945		GACCGCAGCGGC	in frame change in BMI_I1027/BR1024
997958	997958	993690		G		BMI_I1028/BR1025 is shorter in B. suis, hypothetical protein
1006879	1006879	1002610		T		BMI_I1041/BR1038 is different in B. microti and B. suis, basic proline-rich protein precursor.
1014197		1009929	1009939		CTTGACCGTAT	BMI_I1045/BR1042 is pseudogene in B. suis, mechanosensitive ion channel family protein
1025059	1025060	1020800		CT		BMI_I1056/BR1051 has a premature end in B. suis
1047382	1048225	1043121		GGCGTGTCTGCATTTAACGTAACCAGATCATAGCGCATGCGAGATGGTTGAAACCCATGAATGCGGTCAATGTTTTCTCGCATCGCAGCGCAATACGACGATAGCGTTTCAACTTGTTAAAAAAGCATTCAATCTGATGGCGTTCCTTGTACAGCCTCCAGTCGATTGTTGGGACACTGGAACGTGTTGGATTGACCTTGATCTGAGCCGTTGCCTTGAGATTGCTGGCAATGAAGGCCCTTAAGTGATCGGCATCATAGGCCGCGTCAGCAATGACATGTCCCACACCCTTTAAGCCGGATAGAAGGCTTGAAGCTTGCGGACAGTCACCATAATGGCCGGGTGTTGGCTTTATTCGCAGCGGTAGGCCGATAGCATCGACAACAGCATGCAGCTTGGTCGTCAATCCACCGCGCGAGCGACCGATGCAGGCAGCTTCAGCCCCCCTTTTGCGCCCGCCGCATCTGCGTGGACTTTCGATATGGTGCTATCAATGAGGACATATTCAAAGTCCGGCGTATCAGCCAGGGCATGGAAAAGCCTTTCCCATACACCGGCGTGCGACCAGCGCCGAAAGCGGGCATGAACCGCTGTCCATTTGCCGAAGGTCGCAGGCAGATCGCGCCAGTGCGCTGCATTGGCAGCCATCCACAAGATGGCGTCGACAAATAATCGGTTATCGACGCCACTGCGGCCGGGCGTACCAACTCGCCCCGGAAGATATGCTTCGATCCGGTTCCATTGCTCATCTGTAAGGCTTCGTCTGCTCACGGCTGTTCTCCTTCAACAACCTTGAATCAGAATTTCATGCAAAAGGGAATCCTTGAATGCAGACAAGCCCTAG		IS711 insertion sequence
1056022	1056022	1050917		T		between BMI_I1088 and BMI_I1089
1056055		1050951	1050951		C	
1056584		1051481	1051484		GTTC	between BMI_I1089 and BMI_I1090
1059998	1060127	1054897		AATCGGCTTGTTGAGCGTCCAGCGGCTGGATCGCATGAACGCCGGTGGCTGATAAGCTTATTGATGCCCGAGCGGGTGATGAAAGTTGCGTTGGGGTTGCCGCGAATACCCTCATTAATTCTGAGGGTAT		A few differences in region of insertion
1060139	1060155	1054908		CCATCCAATCTGGAGAG		
1060171	1060181	1054923		GCCGCTGGTGT		
1060260		1055003	1055003		G	
1060274	1060276	1055016		AGG		
1060289	1060296	1055028		GATGCTGT		
1060308	1060311	1055039		AGGC		
1060335		1055064	1055064		T	
1060362		1055092	1055100		AAGGATGTC	
1060384	1060384	1055121		C		
1060434	1060709	1055170		GTCTAAGAAGCTGCTCGTCTGGCGTATTCAGTTCCCGCGTCTGCTTCTTCACCTCAATCCCCCGCTCACGGCACAGCACAGCCGCCTTCCGGCTAAGCTTGGTCTTCATCCCATGGGGAAGGTAGAGGTGGCTTAAGGCTCGCCCCGTGCTCCGCACATCATTCGTCCATCGTGACGTATTCTAGGTGTTCCGTGATGATGCCTTGCAAGAACTTAGCCAGAGCTTCGGCAGCCTCTTTCTCTTCCTTCAGGCGCTTCATTTTCTCCTGAAGGCGG		BMI_I1094/BR1083, B. suis gene is shorter, BRO family, N-terminal domain protein
1070587		1065049	1065049		G	BMI_I1102/BR1090 is pseudogene in B. microti
1081290	1081303	1075751		CGCTGAAAGCTTCA		BMI_I1113/BR1101, gene is much shorter in B. suis, hypothetical protein
1086070	1086070	1080516		C		between BMI_I1116/BR1104 and BMI_I1117/BR1105
1110783	1110783	1105228		T		BMI_I1139/BR1127 is pseudogene in B. microti. aceF, pyruvate dehydrogenase complex, E2 component, dihydrolipoamide acetyltransferase
1148588		1143034	1143034		A	between BMI_I1174/BR1163 and BMI_I1175/BR1164
1152688		1147135	1147135		A	between BMI_I1179/BR1168 and BMI_I1169/BR1169
1163216		1157664	1157664		G	BMI_I1191/BR1180, gene is shorter in B. suis, hypothetical protein
1167415		1161864	1161864		T	between BMI_I1198/BR1187 and BMI_I1199/BR1188
1167517	1168280	1161965		GTATCGATGCCCAGCACGGCAAAGGCAAGCTTACCGCGCGCTAAAGAATCGAAGTGCTTCTCGACGAAGGATCGTTCGAGGAGTTCGACATGTATGTCACGCATCGCTGCACCGATTTCGGCATGGCGCAGCAGAAAGTTGCGGGCGACGGCGTGGTAACGGGCTGGGGCACCATCAATGGCCGTCAGGTCTATGTGTTCAGCCAGGACTTCACGGTTCTGGGCGGCTCGCTTTCGGAAACCCATGCGCAAAAAATCTGCAAGATCATGGACATGGCCGTAAAAAACGGCGCGCCGGTCATCGGCCTCAACGATTCCGGCGGCGCGCGCATCCAGGAAGGCGTGGCATCGTTGGCAGGCTATGCCGATGTTTTCAAACGCAATGTGGATGCGTCGGGCGTCGTGCCGCAGATTTCCGTCATCATGGGGCCATGCGCCGGCGGTGCCGTCTATTCCCCGGCCATGACCGACTTCATTTTCATGGTACGCGATTCGTCCTACATGTTTGTGACAGGCCCAGATGTGGTCAAAACCGTCACCAACGAAATCGTGACCGCTGAGGAATTAGGCGGTGCATCCACCCACACGAAAAAATCCTCTGTGGCCGATGGCGCCTATGAGAACGACATTGAGGCTCTGGAACAGGTACGCATCCTGTTCGACTTCCTGCCGCTCAACAATCGCGAGAAGCCACCTGTTCGCCCGATCTTCGATGATCCGGCGCGCCTTGAAATGCGCCTCGACACGCTCGTACCGGACAGCGCC		in BMI_I1199/BR1188, pseudogene both in B. microti and B. suis
1212166	1212183	1205850		CGACGAAGAGGAAGAGGA		BMI_I1251/BR1241, B. suis gene is slightly shorter, hypothetical protein
1224676	1224676	1218342		C		between BMI_I1259/BR1250(secE) and tRNA
1227941	1227941	1221606		T		pseudogene BR1255 is intact in B. microti, BMI_I1267, CBS domain containing protein
1240859		1234525	1234539		TCGAAGCGGGATCAG	between BMI_I1283/BR1272 and BMI_I1284/BR1273
1240864		1234545	1234574		AGTCCAGTGGACTGATTTCCCCGCGTAGGC	
1246069	1246080	1239778		CACTGGCACCGG		in frame change in BMI_I1288/BR1277, putative peptidase M15A

1248306		1242005	1242005		A	between BMI_I1291/BR1278 and BMI_I1292/BR1281, two genes are annotated in B. suis, but they are very small
1248354		1242054	1242073		GGCAAATCTAGAGCATTTCC	
1251779	1252621	1245497		TCTGGAAGATATGCTTTAGGGCCTGTCTGCATTTAACGTAACCAGATCATAGCGCATGCGAGATGGACGAAACCCATGAATGCGGTCAATGTTTTCTCGCATCGCAGCGCAATACGACGATAGCGTTTCAACTTGTTAAAAAAGCATTCAATCATCGATGGCGTTCCTTGGACAGCCTCCAGTCGATTGTTGGGACACTGGAACGTGTTGGATTGGCCTTGGATCTGAGCCGTTGCCATGAGATCGCTGGCAATGAAGGCCCTTAAGTGATCGGCATCATGGGCCGCGTCAGCAATGACATGTCCCACACCCTTTAAGCCGGATAGAAGGCTTGAAGCTTGCGGACAGTCACCATAATGGCCGGGTGTTGGCTTTATTCGCAGCGGTAGGCCGATAGCATCGACAACAGCATGCAGCTTGGTCGTCAATCCACCGCGCGAGCGACCGATGCAGGCAGCTTCAGCCCCCCTTTTGCGCCCGCCGCATCTGCGTGGACTTTCGATATGGTGCTATCAATGAGGACATATTCAAAGTCCGGCGTATCAGCCAGGGCATGGAAAAGCCTTTCCCATACACCGGCGTGCGACCAGCGCCGAAAGCGGGCATGAACCGCTGTCCATTTGCCGAAGGTCGCAGGCAGATCGCGCCAGTGCGCTGCATTGGCAGCCATCCACAAGATGGCGTCGACAAATAATCGGTTATCGACGCCACTGCGGCCGGGCGTACCAACTCGCCCCGGAAGATATGCTTCGATCCGGTTCCATTGCTCATCTGTAAGGCTTCGTCTGCTCACGGCTGTTCTCCTTCAACAACCTTGAATCAGAATTTCATGCAAAAGGGAAT		IS711 insertion sequence
1252629	1252632	1245504		TGCA		
1275517	1275528	1268388		GGCTTCATGATG		in frame change in BMI_I1320/BR1307, cobalamin biosynthesis protein CobW
1282581		1275442	1275442		C	BMI_I1329, Entericidin EcnAB, is annotated as two genes BR1316 and BR1317 in B. suis
1290020	1290020	1282880		T		BMI_I1334/BR1322 is pseudogene in B. suis, MscS mechanosensitive ion channel
1290655	1290666	1283514		TGATACGGGGCT		
1318218	1318226	1311065		AGCATGGCC		in-frame change in BMI_I1361/BR1350, ABC transporter, ATP binding/permease protein
1321957	1321957	1314795		G		BMI_I1363/BR1353, B. microti ortholog is shorter, hypothetical protein
1330321		1323160	1323162		GGT	in-frame change in BMI_I1373/BR1363, putative urea transporter
1334399		1327241	1327241		G	BMI_I1379/BR1369 is pseudogene in B. microti, crcB family protein
1336819	1336819	1329660		C		between BMI_I1381/BR1371 and BMI_I1382/BR1372
1338815	1338883	1331655		GGCAAGGTCGATACCCGTTTCCTCCCGCACTTCCCTCCGGCAATTGGCATCATAATCGACGCGCGCATC		in-frame change in BMI_I1385/BR1375, NUDIX hydrolase
1339622	1339626	1332393		CCTCT		BMI_I1386/BR1376 is pseudogene in B. suis, endonuclease/exonuclease/phosphatase family protein
1341035	1341035	1333801		T		between BMI_I1387/BR1377 and BMI_I1388/BR1378
1342301	1342351	1335066		TTAGACTATCCGCCTCTTGCAGCGGAACTTGTAATAGCGAAGTCTCCTCAA		between BMI_I1388/BR1378 and BMI_I1389/BR1379 ortholog
1342678	1342764	1335391		TCCGGCGGCGCCGGGCAAGCAAAAGAAAAGTGCATCGAACTAATCAGTTGCGGGCTTTATCGACCAGTTTGTTCTTTTCGATCACCC		between BMI_I1389/BR1379 and BMI_I1390/BR1380
1344723	1344723	1337349		T		BMI_I1392/BR1382, hypothetical protein. B. microti ortholog is longer, and widely overlap next gene BMI_I1393/BR1383, potassium uptake protein
1359683	1359685	1352308		GCC		in-frame change in BMI_I1408/BR1397, hflk protein
1365856	1365856	1358478		C		BMI_I1419/BR1407, hypothetical protein. B. microti ortholog is longer and overlaps next gene BMI_I1420/ BR1408
1378513	1378529	1371134		GCGGGTCATCTGAAATA		small difference at the end of BMI_I1429/BR1417, overlapping stop codon, ribosomal protein L11 methyltransferase
1406973	1406988	1399577		AGGGCAGTAGGGCAGT		between BMI_I1452/BR1440 and BMI_I1452/BR1441. Bruce09*
1407059	1407065	1399646		TATTCCC		
1407107	1407113	1399686		TGCCTTA		
1414176	1414176	1406748		A		BMI_I1460/BR1448 is pseudogene in B. suis, lytic murein transglycosilase
1415613	1415613	1408184		C		BMI_I1461/BR1449 is pseudogene in B. suis, auxin efflux carrier
1428649	1428649	1421219		C		BMI_I1475/BR1463, B.suis gene is shorter at the end, other Brucella are like B. microti
1434374	1434385	1426943		CGCCACGCGCAA		BMI_I1483/BR1471, B. microti ortholog is much longer, ortholog much longer in B. microti, hypothetical protein
1436887	1437731	1429443		GGGCCTGTCTGCATTCAACGCAACCAGATCATAGCGCATGCGAGATGGACGAAACCCATGAATGCGGTCAATGTTTTCTCGCATCGCAGCGCAATGCGACGATAGCGTTTCAACTTGTTAAAAAAGCATTCAATCTGATGGCGTTCCTTGTACAGCCTCCAGTCGATTGTTGGGGCACTGGAACGTGTTGGATTGGCCTTGGATCTGAGCCGTTGCCTTGAGATCGCTGGCAATGAAGGCCCTTAAGTGATCGGCATCATGGGCCGCGTCAGCAATGACATGTCCCACACCCTTTAAGCCGGATAGAAGGCTTGAAGCTTGCGGACAGTCACCATAATGGCCGGGTGTTGGCTTTATTCGCAGCGGTAGGCCGATAGCATCGACAACAGCATGCAGCTTGGTCGTCAATCCACCGCGCGAGCGACCGATGCAGGCAGCTTCAGCCCCCCTTTTGCGCCCGCCGCATCTGCGTGGACTTTCGATATGGTGCTATCAATGAGGACATATTCAAAGTCCGGCGTATCAGCCAGGGCATGGAAAAGCCTTTCCCATACACCGGCGTGCGACCAGCGCCGAAAGCGGGCATGAACCGCTGTCCATTTGCCGAAGGTCGCAGGCAGATCGCGCCAGTGCGCTGCATTGGCAGCCATCCACAAGATGGCGTCGACAAATAATCGGTTATCGACGCCACTGCGGCCGGGCGTACCAACTCGCCCCGGAAGATATGCTTCGATCCGGTTCCATTGCTCATCTGTAAGGCTTCGTCTGCTCACGGCTGTTCTCCTTCAACAACCTTGAATCAGAATTTCATGCAAAAGGGAATCCTTGTATGCAGACAGGCCCTA		IS711 insertion sequence
1438765		1430478	1430478		T	between BMI_I1490/BR1475 and BMI_I1491/BR1476
1478244	1478246	1469956		GCC		in-frame change in BMI_I1530/BR1516, hypothetical protein
1483321		1475032	1475093		TCCTGAAAAGTGTGAAACGGTTTTCGGAAAAGATGCGCGTCAAAGCAAAGAATTAGAGCCCA	insert corresponding to BR1523, hypothetical protein, part of this sequence is present multiple times in all brucella
1501458		1493231	1493231		C	BMI_I1558/BR1544, is pseudogene in B. suis, ABC transporter, permease/ATP-binding protein
1505288		1497062	1497062		T	between BMI_I1559/BR1545 and BMI_I1560/BR1546
1509485	1509485	1501258		T		BMI_I1566/BR1552 is pseudogene in B. suis, aspartyl/ asparaginyl beta-hydroxylase
1512681		1504455	1504455		G	BMI_I1569/BR1555 is smaller in B. microti, hypothetical protein
1541052	1541078	1532825		GCCGAGGCTCATGGAGATGATGACGAT		BMI_I1597/BR1584 is pseudogene in B. microti, dipeptide ABC transporter, permease protein
1541241		1532989	1532989		G	
1545072	1545073	1536819		TG		BMI_I1601/BR1588 is pseudogene in B. microti, hypothetical protein
1557452		1549199	1549199		C	between BMI_I1619/BR1605 and tRNA
1571619	1571743	1563365		GGGGTGATCTCTTGATCCCGGTCATCGTCACCCACGAAAAATGATGGCGCTCTCAACAATTGCCGTCGCCGCCATGTTTCTTCTCTTTGTGTGCCTTCGGCAACACGGGAACGGCGACGGCAACT		between BMI_I1632/BR1617 and tRNA. Bruce42*
1571824		1563447	1563447		T	
1571949	1571949	1563571		T		
1590995	1590995	1582616		G		BMI_I1654/BR1636 is pseudogene in B. suis, choline dehydrogenase
1595933		1587554	1587567		GTGCTGTTCTCGCT	23S ribosomal rna, difference in IVS region
1595943		1587578	1587585		ACGCATTC	
1595970	1595983	1587611		CAATAAGGCAATAT		
1595996	1595997	1587623		CT		
1596017		1587644	1587644		G	
1596030		1587658	1587674		CCATCCAGCGTTGCTCC	
1617267	1617278	1608910		GGGTAAACGGGC		between BMI_I1682/BR1659 and BMI_I1683/BR1660. Bruce43*
1625415	1625415	1617046		T		BMI_I1694/BR1671 is pseudogene in B. microti, HlyD family secretion protein

1626452		1618084	1618927		GGGCTTGTCTGCATTCAAGGATTCCCTTTTGCATGAAATTC<br>TGATTCAAGGTTGTTGAAGGAGAACAGCCGTGAGCAGACGA<br>AGCCTTACAGATGAGCAATGGAACCGGATCGAAGCATATCT<br>TCCGGGGCGAGTTGGTACGCCCGGCCGCAGTGGCGTTGATA<br>ACCGATTATTTGTCGACGCCATCTTGTGGATGGCTGCCAAT<br>GCAGCGCACTGGCGCGATCTGCCTGCGACCTTCGGCAAATG<br>GACAGCGGTTCATGCCCGCTTTCGGCGATGGTCGCACGCCG<br>GTGTATGGGGAAGGCTTTTCCATGCCCTGGCTGATACGCCG<br>GACTTTGAATATGTCCTCATTGATAGCACCATATCGAAAGT<br>CCACGCAGATGCGGCGGGCGCAAAAGGGGGGCTGAAGCTGC<br>CTGCATCGGTCGCTCGCGCGGTGGATTGACGACCAAGCTGC<br>ATGCTGTTGTCGATGCTATCGGCCTACCGCTGCGAATAAAG<br>CCAACACCCGGCCATTATGGTGACTGTCCGCAAGCTTCAAG<br>CCTTCTATCCGGCTTGAAGGGTGTGGGGCATGTCATTGCTG<br>ATGCAGCCTATGATGCCGATCACTTAAGGGCCTTCATTGCC<br>AGCGATCTCAAGGCAACGGCTCAGATCAAGGTCAATCCAAC<br>ACGTTCCAGTGTCCCAACAATCGACTGGAGGCTGTACAAGG<br>AACGCCATCAGATTGAATGCTTTTTTAACAAGTTGAAACGC<br>TATCGTCGTATTGCGCTGCGATGCGAGAAAACATTGACCGC<br>ATTCATGGGTTTCGTCCATCTCGCATGCGCTATGATCTGGT<br>TACGTTAAATGCAGACACGCCCTA	IS711 insertion sequence specific to B. suis
1637448	1637448	1629923		G		between BMI_I1706/BR1685 and BMI_I1707/BR1687
1653124	1653140	1645598		ATAAAGCAGATGCGCGC		between BMI_I1721/BR1701 and BMI_I1722/BR1702
1656327	1656327	1648784		A		between BMI_I1725/BR1705 and BMI_I1726/BR1706
1656749		1649207	1649219		ACGAAGACCGGCA	BMI_I1726/BR1706 is pseudogene in B. microti , putative OpgC protein
1667700		1660171	1660171		C	BMI_I1737/BR1718 is pseudogene in B. suis, transcriptionnal regulator, gntR family
1682384		1674856	1674856		C	between BMI_I1755/BR1736 and BMI_I1756/BR1737
1708214		1700687	1700687		T	BMI_I1788/BR1770 is pseudogene in B. microti, ATP-dependent helicase
1716495		1708969	1708969		G	BMI_I1798/BR1780 is pseudogene in B. suis, membrane protein MosC
1725186	1725187	1717659		CG		BMI_I1805/BR1788 is pseudogene in B. suis, putative ATP-binding component of ABC transporter
1731263	1731263	1723734		C		BMI_I1810/BR1793 is pseudogene in B. suis, D-amino acid dehydrogenase small subunit
1774089	1774193	1766559		ATATCCCGAAGGGACGCGGTGTATTTTGCTTCTGAATGTGTCG<br>AAACGTCCTTGTGGAGATCACTCCACGGCGGTCGTTTCTCCTG<br>TTCAGCAACAAAATACCCT		between BMI_I1849/BR1833 and BMI_I1850/BR1834
1785632	1786009	1777997		CCAAGAGCGGAAATCGCAGCACTATTCTGTCTGATAGCAGCCC<br>TATTGCTAGCAACACCTGCTGCGTTAGCATTGATGCGACCGCG<br>GTTTTGGCTGATGGCAGCCCTATTGTCGGCAACACCTTTGGCA<br>TTGGTATCAATCCGGCCACTGTTTCGACCAATGGCAGCCCTAT<br>TGTCAGCAACGCCCTTGGCATTGGTGTCAATCCGGCCGCTGTT<br>CCGGCCAATGGCAGCCCGGTTGTCGGCAACACCTTTGGCATTG<br>GTGTCGATCCGACCGCTGTTCCGGCCAATGGCAGTCTTGTTGT<br>CGGCAACGCCCTTGGCATTGGCGTCGATCCGGACACTATTCTG<br>GCCAATGGCAGTCTTGTTGTCGGCAACGCCCTTG		in frame change in BMI_I1862/BR1846, insert in gene BR1846 ortholog, hypothetical protein, different in other brucella. Bruce11*
1786832		1778821	1778821		A	between BMI_I1862/BR1846 and BMI_I1863/BR1847
1795275		1787265	1787265		T	BMI_I1873/BR1856 is pseudogene in B. microti, hypothetical protein
1800625		1792616	1792629		GTGCTGTTCTCGCT	23S ribosomal rna, difference in IVS region
1800635		1792640	1792647		ACGCATTC	
1800662	1800675	1792673		CAATAAGGCAATAT		
1800688	1800689	1792685		CT		
1800709		1792706	1792706		G	
1800722		1792720	1792736		CCATCCAGCGTTGCTCC	
1807684	1807684	1799697		T		between BMI_I1883/BR1862 and BMI_I1884/BR1864
1823468	1824311	1815480		GGCTTGTCTGCATTCAAGGATTCCCTTTTGCATGAAATTCTGA<br>TTCAAGGTTGTTGAAGGAGAACAGCCGTGAGCAGACGAAGCCT<br>TACAGATGAGCAATGGAACCGGATCGAAGCATATCTTCCGGGG<br>CGAGTTGGTACGCCCGGCCGCAGTGGCGTCGATAACCGATTAT<br>TTGTCGACGCCATCTTGTGGATGGCTGCCAATGCAGCGCACTG<br>GCGCGATCTGCCTGCGACCTTCGGCAAATGGACAGCGGTTCAT<br>GCCCGCTTTCGGCGCTGGTCGCACGCCGGTGTATGGGAAAGGC<br>TTTTCCATGCCCTGGCTGATACGCCGGACTTTGAATATGTCCT<br>CATTGATAGCACCATATCGAAAGTCCACGCAGATGCGGCGGGC<br>GCAAAAGGGGGGCTGAAGCTGCCTGCATCGGTCGCTCGCGCGG<br>TGGATTGACGACCAAGCTGCATGCTGTTGTCGATGCTATCGGC<br>CTACCGCTGCGAATAAAGCCAACACCCGGCCATTATGGTGACT<br>GTCCGCAAGCTTCAAGCCTTCTATCCGGCTTAAAGGGTGTGGG<br>ACATGTCATTGCTGACGCGGCCTATGATGCCGATCACTTAAGG<br>GCCTTCATTGCCAGCAATCTCAAGGCAACGGCTCAGATCAAGG<br>TCAATCCAACACGTTCCAGTGTCCCAACAATCGACTGGAGGCT<br>GTACAAGGAACGCCATCAGATTGAATGCTTTTTTAACAAGTTG<br>AAACGCTATCGTCGTATTGCGCTGCGATGCGAGAAAACATTGA<br>CCGCATTCATGGGTTTCGTCCATCTCGCATGCGCTATGATCTG<br>GTTACGTTAAATGCAGACACGCCCTAG		IS711 insertion sequence
1825449		1816619	1816619		G	BMI_I1903/BR1881 is pseudogene in B. suis, major facilitator superfamily transporter
1836378	1836378	1827547		G		BMI_I1915/BR1894 is pseudogene in B. suis, GntR family transcriptionnal regulator
1841776	1841776	1832944		T		BMI_I1919/BR1898 is a bit shorter in B. suis, acyl-CoA thioesterase II. B. microti gene is like in most other brucella
1844787	1844907	1835954		GCGCCTCTGGCGCGACAGGGACTAAAAAGACCAACGATTGCCG<br>TGGTTACACGCGGCGTTCGACCATCATCTTCTTGATCTCGGCA<br>ACCGTGCCTTCGGGCACGGATGACAAGAGGCGTGG		between BMI_I1921/BR1900 and BMI_I1923/BR1901.
1844918		1835966	1835966		G	
1844928	1844929	1835975		AA		
1870834	1870862	1861879		CTTTCTCTCAGGCTGCGCCTACATTTTGA		between BMI_I1950/BR1928 and BMI_I1951/BR1929. Bruce48*
1890905	1890905	1881921		G		BMI_I1972/BR1950 is slightly shorter in B. microti, hypothetical protein
1893576		1884593	1884593		C	BMI_I1976/BR1954 is pseudogene in B. suis, ABC transporter, permease protein
1922366	1922380	1913382		AAGGTCAGAGTCAGG		in-frame change in BMI_I2007/BR1984, hypothetical protein. Bruce51*
1939535	1939537	1930536		TGG		in-frame change in BMI_I2031/BR2009, intimin/invasin family protein. Bruce55*
1947302		1938302	1938307		GGGGGG	in-frame change in BMI_I2035/BR2013, outer membrane autotransporter
1972250	1972250	1963254		C		BMI_I2058/BR2037 is pseudogene in B. microti, peptidase, M20/M25/M40 family
1988486	1988486	1979489		A		between BMI_I2076/BR2054 and BMI_I2077/BR2055
2015219		2006223	2006223		G	between BMI_I2103/BR2081 and BMI_I2104/BR2082
2020009		2011014	2011014		C	BMI_I2110/BR2088 is smaller in B. microti, class I peptide chain release factor. B. suis predicted gene is longer than in other brucella
2021775		2012781	2012789		GAAATTTTT	between BMI_I2111/BR2089 and BMI_I2112/BR2090
2052733		2043748	2043760		TGGCGATAAATTT	between BMI_I2142/BR2120 and BMI_I2143/BR2121
2053634	2053793	2044660		CAAGAGGCGTGACGTGCCGTAGGCACGGCAGGGATATAATCAA<br>GAGGCGTGGAGTGCCGTAGGCGCGACAGGGATTTAACCAAGAG<br>GCGTGGAGTGCCGCAGGCGCGACAGGGATTTAACCAAGAGGCG<br>TGGAGTGCCGCAGGCGCGACAGGGATTTAAC		between BMI_I2143/BR2121 and BMI_I2144/BR2122
2062290	2062292	2053156		TTG		in-frame change in BMI_I2149/BR2127, phospholipid N-methyltransferase
2066992	2066992	2057855		G		BMI_I2154/BR2132 is pseudogene in B. microti, Mg chelatase protein
2068556	2068556	2059418		G		between BMI_I2155/BR2133 and BMI_I2156/BR2135
2094542	2094604	2085403		ATTTCCAGCAAAAGTGCGAAGCGGTTTTGCGTCGGATAATGCG<br>ACAAAACAAAGAGGTAGAGC		between BMI_I2184/BR2163 and BMI_I2185/BR2164
2096233		2087032	2087082		CTGGCCGCCCGCAAGGTGGTCGCCCGCAGCCGGCCGGAAGG<br>CCGCAGCAGG	in-frame change in BMI_I2186/BR2165, translation initiation factor IF-2
2110433	2110537	2101281		CATATTGGGCGGGATTTTCCACCGGAGCGCCGTGGTTTGAATT<br>GGGCAGGATAATCAATTCCGCGCCCGGAATCTGCTGCGCGATG<br>CATTCGGTATGCGCACGGG		BMI_I2199/BR2178 is slightly longer in B. microti, putative hydrolase. B. microti gene is similar to O. anthropi

Insertion-deletions in Chromosome 2

Coordinates				Insertion *B. microti*	Insertion *B. suis*	Description
B. microti		*B. suis 1330*				
9091		9092	9092		A	between BMI_II10/BRA0010 and BMI_II11/BRA0011
15077		15079	15079		C	BMI_II17/BRA0017 is shorter in B. microti, enterobactin synthetase, component F
18203	18232	18204		GTCACCTTTCATGGCGTCGCCTTTCATCGC		in-frame change in BMI_II20/BRA0020, and gene is also predicted longer in B. microti. Bruce12*
18290	18304	18261		GCCCTTCATGGCATC		
19730		19688	19688		G	BMI_II22/BRA0022 is longer in B. microti, gene ortholog is longer at the 3' end in B. microti, extracytoplasmic function alternative sigma factor
54710		54669	54672		ATAT	between BMI_II58/BRA0056 and BMI_II59/BRA0057
67900	67911	67861		GATATTGCCGTC		in-frame change in BMI_II72/BRA0070, membrane-bound lytic murein transglycosylase B. Bruce60*
73073	73120	73022		GGAGTAAGGGAGTAAGGGAGTAAGGGAGTAAGGGAGTAAGGGA<br>GTAAG		between BMI_II75/BRA0073 and BMI_II76/BRA0074. Bruce13*,Bruce14*
73208		73111	73158		TTCCCCTATTCCCCTATTCCCCTATTCCCCTATTCCCCTAT<br>TCCCCTA	
75896	75896	75845		A		between BMI_II78/BRA0076 and BMI_II79/BRA0077
84694		84644	84720		ACGGCGCGCCCGGCGCGGGCAACCGCGCCCACGCGGCAGGC<br>CCCGGCAGCCGTGCCGGCGGCGCCTGTGGAACAGCC	BMI_II85/BRA0083 is pseudogene in B. microti, hypothetical protein
86358	86359	86383		GC		BMI_II86/BRA0084 is pseudogene in B. suis, 2-dehydro-3-deoxyphosphogluconate aldolase/4-hydroxy-2-oxoglutarate aldolase
92504		92529	92529		A	BMI_II95/BRA0093-5 pseudogene in B. microti and probably also in B. suis
97039		97065	97065		A	between BMI_II99/BRA0099 and BMI_II100/BRA0100
116968		116995	116995		A	BMI_II117/BRA0117, pseudogene in B. microti and B. suis, HlyD secretion family protein
118148	118148	118174		T		between BMI_II118/BRA0118 and BMI_II119/BRA0119
121135		121162	121176		ATCGTCGCGGTTGAG	BMI_II122/BRA00122 is pseudogene in B. microti, flagellar motor switch protein FliG
127138	127230	127178		TTTCCGATACTATTCGCGGAACGCTGGACGGCCACATTGTGCT<br>CGACCGCGCCATTGCCGCGCAGGGGCGCTTTCCGGCGGTGGAT<br>ATTCCGG		in-frame change in BMI_II129/BRA0129, fliI flagellum specific ATP synthase
133387	133387	133334		T		between BMI_II137/BRA0137 and BMI_II138/BRA0139. A small hypothetical gene is annotated here in B. suis 1330.
141627		141575	141575		G	BMI_II147/BRA0149 is pseudogene in B. suis, 3-hydroxyisobutyrate dehydrogenase family protein
141709	141709	141656		A		
156213	156213	156159		G		BMI_II170/BRA0173, quite variable outer membrane autotransporter. Bruce15*
156271	156272	156215		GT		
157377	158447	157319		AACGGCGGCACTTTGCAGGTGACGGGGACGGGTTGGACGTCAA<br>CGAGTCGGGCGGTGAGCCTGCAGGCTGGCGGCGGCACTTTCGA<br>CATTGAGGATGCGGCGAATAATTTTGCGGTCACGCAAGGTGTG<br>ACCGGCGCTGGCGGTCTGATGAAGAGCGGCACAGGCACGGTTA<br>CGCTTTCTGGTGCCAACAGCTATACTGGTGGCACGAAGGTGTC<br>TGCAGGCACGCTTGTGGTGGCGAACGACAACACCGGTGGCGGC<br>ACGACGACGGTTGATGCGGGGGCAGGACTTCAGATTGGCACTG<br>GCGGGACGAGCGGCAGCCTTGCGGGCGATATCGCCAACAATGG<br>TGCGCTTGTCGTAAACCGCTCCGACGCACTTAATCTCGCCGGC<br>GCGATCTCCGGCACGGGCAGTCTGACGAAGAACGGTGCTGGCA<br>CGCTGACGCTTTCTGGTGCCAACAGCTATACGGGCGCAACGAC<br>GGTGTCTGCGGGCACGCTGACGCTAACGGGTGACAATACCGGT<br>GGCGGCACGACGACGGTTGATGTGGGAGCCTTGCTTCAGATTG<br>GCACTGGCGGGACGAGCGGCAGCCTTGCAGGCGATATCGCCAA<br>TGATGGTACGCTTGTCGTTGACCGCTCCGACGCGATCGGGCTT<br>AGCGGAGCGATCTCCGGCGCGGGCGGCCTGACGAAGAACGGTG<br>CTGGCACGCTGACGCTTTCTGGTGCCAACAGCTATACGGGCGC<br>AACGACGGTGTCTGCGGGCATACTGACGTTGACGGGTGACAAT<br>ACCGGTGGCGGCACGACGACGGTTGATGCGAGGGCAGAACTTC<br>ATATCGGTACTGGTGGGGCGAGCGGCAGCCTTGCAGGCAATAT<br>CGTCAATGATGGTGCGCTTGTCGTTGACCGTAGCGGTGTGATC<br>GGGCTTAGCGGTGTGATCTCCGGCACGGGCGGTCTGATGAAGA<br>GCGGCACAGGCACGGTTACGCTTTCTGGTGTCAACACCTATAC<br>GGGTGGCACGACGTTGACGGCGGGCACGCTTGCTGCTGCTTCC<br>GATAATAATCTGGGTGGTGCTTCGGGCGGTCTGACGTTT		
160764	160764	159635		C		between BMI_II170/BRA0173 and BMI_II171/BRA00174
162162		161034	161034		T	between BMI_II172/BRA0175 and BMI_II173/BRA00176
168819		167692	167692		T	between BMI_II179/BRA0182 and BMI_II180/BRA0183
179531	179531	178403		A		between BMI_II189/BRA0192 and BMI_II190/BRA0193
193934		192807	192808		TA	BMI_II204 (peptidase M16 domain protein),BMI_II205 (zinc protease), corresponding to pseudogene BRA0208 in B. suis
194893	194924	193766		TGCGGGCGATCTCGATTCCGATGCCTTTCAGG		
195960	195960	194801		A		
214786	214833	213626		AAATCGAGAAAATAGGCCTCGCGCGCCAATGTCCGCTCATCGC<br>GGGCG		BMI_II222/BRA0225 is pseudogene in B. suis
214952	215008	213743		GGAAATCAGCCGAATTAAGCTTCACGGCAACGCAAAAGCCCGG<br>CGAAACCGCCGCGC		
218867	218875	217601		CTCGGAAAG		BMI_II226/BRA0229 is pseudogene in B. suis, two component response regulator
233249	233250	231974		GA		BMI_II243/BRA0249 has a few amino-acid differences as a result of those 2 nearby indels, nitric-oxide reductase, large subunit
233261		231986	231987		TC	
243750	243750	242475		G		between BMI_II254/BRA0260 and BMI_II255/BRA0261
249095	249100	247819		CTTGGC		in-frame change in BMI_II260/BRA0266, galactoside transport ATB-binding protein
250632	250632	249350		G		between BMI_II261/BRA0267 and BMI_II262/BRA0268
254793	254793	253510		T		BMI_II266/BRA0272 is pseudogene in B. suis, ABC transporter related protein
256799	256799	255515		C		small difference in small hypothetical protein BMI_II268/ BRA0273
309794		308511	308521		CAAGCGGTGGA	BMI_II323/BRA0328 is pseudogene in B. microti, spermidine/putrescine ABC transporter, permease protein
321000		319728	319729		TT	BMI_II335/BRA0339 is pseudogene in B. suis, amino-acid antiporter, probably working together with previous gene BMI_II334/BRA0338, also a pseudogene in B. suis
336537		335267	335267		G	between BMI_II348/BRA0353 and BMI_II349/BRA0354
347951	348014	346680		TGAAACCGTCTATTAGGAACCGTTTCGCCGTCTATTAGGAACT<br>GGTATCCGTCTATTAGGAACC		between BMI_II359/BRA0364 and BMI_II360/BRA0365
362793		361460	361463		CCCC	between BMI_II376/BRA0379 and BMI_II377/BRA0380
376577		375248	375255		ATGGGGAT	BMI_II391/BRA0394 is pseudogene in B. suis, ortholog is intact in B. microti, branched-chain amino acid ABC transporter, permease/ATP-binding
382258	382260	380935		CGG		in-frame change in BMI_II396/BRA0399, aldehyde dehydrogenase family protein
388161		386837	386838		TA	BMI_II400/BRA0403 is pseudogene in B. microti, oxidoreductase, Gfo/Idh/MocA family
398376	398376	397052		T		between BMI_II409/BRA0412 and BMI_II410/BRA0413
415806		414483	414488		AGAAGG	small difference at the beginning of gene BMI_II424/ BRA0427, glycosyl transferase, group 2 family protein
449459		448142	448183		TGCAGCGGGCTGATCTGATAAGCGCGACTGGGAGGAGGAGT<br>G	just before gene BMI_II458/BRA0461, hypothetical protein
452041	452053	450764		AAGCAACGGCTGG		BMI_II461/BRA0464 is pseudogene in B. suis, daunorubicin resistance ATP-binding protein
457397		456109	456109		A	BMI_II467/BRA0470 is pseudogene in B. suis, transcriptional regulator, MarR family
463387	463387	462098		A		BMI_II474/BRA0477 is longer in B. microti, hypothetical protein
463453	463488	462163		AAAGGCGGAGATGAAGAAATTAGAGAAGGCGCACAA		
464394	464471	463068		ATGTTTGTAGCGGAAGGGCAGAGGCGGCGTCTTGTTTTCAACC<br>CGGCTGCGGATCACCTTTGCGACATAAGCGCCCTG		in-frame change in BMI_II476/BRA0479, NADH dehydrogenase
520592		519190	519190		C	between BMI_II531/BRA0537 and BMI_II532/BRA0538
531256	531296	529853		GGGAGGGAGCTCATACCCGCTGGATGAAAACATCCAGCGGG		between BMI_II542/BRA0548 and BMI_II543/BRA0549

537481		536039	536048		GGAATCGACT	BMI_II549/BRA0566, difference in IS66 mobile element, orf3
543488		542056	542056		T	between BMI_II557/BRA0563 and BMI_II558/BRA0564
557770		556339	556339		G	between BMI_II570/BRA0576 and BMI_II571/BRA0577
607521		606090	606090		T	between BMI_II615/BRA0618 and BMI_II616/BRA0619
607453	607453	606021		T		
616443	616443	615011		T		between BMI_II629/BRA00633 and BMI_II630/BRA00634
626790	626790	625357		T		BMI_II640/BRA0643 is pseudogene in B. microti, 3-oxoadipate enol-lactone hydrolase
627864		626432	626432		C	BMI_II642/BRA0645 is pseudogene in B. microti, protocatechuate 3,4-dioxygenase, beta subunit
630851		629420	629420		A	BMI_II645/BRA0648 is pseudogene in B. microti, amino acid ABC transporter, periplasmic amino-acid binding protein
642442	642442	641010		C		BMI_II656/BRA0659 is pseudogene in B. microti, transporter, TrkA family
643647		642216	642725		GCGGTCGCGGCAACCGCTTTCAGCATCATGCCGGTGGCTGTCGCCTTTTTCGCAGCCGCAGTCGCCATGCTGGTTTTCAAGGTGATCCCGCTCAATGAGGTCTATGACGAGATCGACGGCCCGATTCTGGTCATGCTGGCCGCGCTCATTCCCGTTTCGGATGCGCTGAGAACCACAGGCGGCACGGAGATTATCGCGCAAGGTCTCGCCTCGCTCGGGCAGCAATTGCCGCCCGCAGGCGCGCTGGCGCTGATATTGGTTGCGGCCATGATGGTGACGCCATTTCCCAACAATGCCGCGACCGTGCTGGTTATGGCCCCCATCGCCACCAGTTTTGCGGCCGGGCTCGGCTTCAGGCCGGAAGCCTTTCTCATGGCCGTCGCCATCGGGGCGGGCTGCGATTTTCTCACCCCCATCGGCCATCAATGCAACACGCTGGTCATGGGGCCGGGTGGCTACAAATTCAGCGATTATCCCCGGCTTGGCCTGCCGCTTTCCATCATCATCGCC	
646403		645482	645498		AAAGCGCAAAGATCACA	BMI_II659/BRA0663 is pseudogene in B. suis, hypothetical protein. Bruce66*
649127		648223	648224		GG	BMI_II664/BRA0667 is pseudogene in B. suis, aminotransferase, class IV
652636		651734	651734		A	between BMI_II668/BRA0671 and BMI_II669/BRA0673. A gene (BRA0672) is annotated on the other strand in B. suis
662491	662505	661588		CGAGGTATGCCGCAA		in frame change in BMI_II676/BRA0681, N-methylhydantoinase (ATP-hydrolyzing) / 5-oxoprolinase
669484		668568	668568		T	BMI_II683/BRA0688 is pseudogene in B. microti, hypothetical protein
670163	670163	669246		G		BMI_II684/BRA0690 is pseudogene in B. suis, maltose/ maltodextrin import ATP-binding protein
676034	676034	675116		A		BMI_II690/BRA0696 is pseudogene in B. suis, formate dehydrogenase accessory protein
681096		680179	680180		GC	BMI_II694/BRA0701 is pseudogene in B. microti, iron compound ABC transporter, ATP-binding protein, putative
684087	684087	683170		T		between BMI_II697/BRA0703 and BMI_II698/BRA0704
689236	689266	688318		AATATCGATTTTAACGATTAAAGCGATTATT		between BMI_II702/BRA0708 and BMI_II703/BRA0709. Bruce67*
691530	691541	690581		CATAGGCCGCCG		in-frame change in BMI_II705/BRA0711, myo-inositol 2-dehydrogenase
693912		692953	692995		GAAGCCGCCGTTTTGAAGGCGCTGCGCCTTGCGCGCGAAAACG	BMI_II707/BRA0713 is pseudogene in B. microti and B. suis, myo-inositol catabolism IolC protein
694766	694766	693848		C		
698234		697317	697317		A	BMI_II710/BRA0716 is pseudogene in B. suis, myo-inositol catabolism IolB domain protein
709421	709444	708503		GCTATGATGACCGCCGTCGACGCC		between BMI_II716/BRA0723 and BMI_II718/BRA0725
715053		714113	714121		TCCCCCCCC	between BMI_II721/BRA0728 and BMI_II722/BRA0729
736532		735601	735603		CCC	in frame change in BMI_II747/BRA0754, iron compound ABC transporter, ATP-binding protein
750671	750734	749741		GGGAGTAAGGGAGTAAGGGAGTAAGGGAGTAAGGGAGTAAGGGAGTAAGGGAGTAAGGGAGTAA		between BMI_II761/BRA0768 and BMI_II762/BRA0769. Bruce16*
762279		761287	761287		A	BMI_II773/BRA0780 is pseudogene in B. suis, phosphogluconate dehydratase
775779	775779	774786		A		between BMI_II786/BRA0792 and BMI_II787/BRA0793
781138	781146	780144		AATCAATAA		in-frame change in BMI_II790/BRA0796, hypothetical protein
782329	782329	781326		T		BMI_II792/BRA0798 is pseudogene in B. suis, transcriptional regulator, LysR family
793204	793204	792200		A		BMI_II802/BRA0808 is pseudogene in B. suis, ABC transporter, permease protein
796885	796885	795880		G		BMI_II805/BRA0811is pseudogene in B. suis, mannitol dehydrogenase
802627	802627	801621		C		BMI_II809/BRA0815 is pseudogene in B. suis, putative multidrug efflux protein
809995	810100	808988		GGACATACGCGCGCTTTTTGAAATCGGCAATCGCTATATGGAAGGGCGCGGTGTTGCCGAAAACGTGAAAGAAGCGGCGAAATGGTATCAGCTTGCCGCCGATCAG		BMI_II815/BRA0822 has some differences, putative peptidoglycan-binding protein
810111	810112	808998		CT		
833080		831967	831967		C	BMI_II839/BRA0845 is pseudogene in B. suis, hypothetical protein
840939		839827	839828		CA	BMI_II848/BRA0854 is pseudogene in B. suis, oxidoreductase, FAD-binding
867085	867085	865973		G		BMI_II875/BRA0881 is pseudogene in B. suis, putative citrate lyase, beta subunit
874655		873544	873544		G	BMI_II883/BRA0889 is pseudogene in B. microti, ada regulatory protein, putative
880347	880361	879235		GACTCCATCGACAGG		in frame change in BMI_II891/BRA0896, hypothetical protein
914103		912978	912978		T	between BMI_II920/BRA0926 and BMI_II921/BRA0927
933802	933859	932676		TCGAGGCGGGGTTCGACGAGCCTTTCGTCGATCATCTTGCCGAAATGCGCATGGCGCT		BMI_II938/BRA0944 is pseudogene in B. suis, transcriptional regulator, GntR family
933879	933880	932694		GC		
960317	960340	959130		GAGTAAGGGAGTAAGGGAGTAAGG		between BMI_II964/BRA0970 and BMI_II965/BRA0971. Bruce18*, Bruce70*
960391	960405	959179		CTGCCCTACTGCCTT		
963641		962416	962417		AT	between BMI_II967/BRA0973 and BMI_II968/BRA0974
965722	965722	964497		A		BMI_II971/BRA0978 is pseudogene in B. suis, beta-lactamase
971081	971120	969855		AGGGCAGTAGGGCAGTAGGGCAGTAGGGCAGTAGGGCAGT		between BMI_II975/BRA0982 and BMI_II976/BRA0983. Bruce20*
975827		974563	974655		CGACCACGATCATCATCACCATGGGCATGACCATCACCATCATGATCATCACGATCACGACCATGTTTGCGGGCCGGACTGCGACCACGATCA	in-frame change in BMI_II980/BRA0987, cobalamin synthesis protein P47K. Bruce19*
977267		976096	976103		AGGGCAGT	between BMI_II981/BRA0988 and BMI_II982/BRA0989. Bruce22*, Bruce71* and Bruce72*
977316	977331	976151		CTCCCTTATTCCCCTA		
977359	977381	976177		TACTGCCTTACTGCCCTACTGCC		
997813		996610	996611		CC	BMI_II1000/BRA1007 is pseudogene in B. suis and B. microti
1025355		1024154	1024160		TCCCCCC	between BMI_II1032/BRA1038 and BMI_II1033/BRA1039
1038732		1037538	1037538		A	between BMI_II1046/BRA1052 and tRNA

1038883	1050624	1037688		AAATTCCGTTTTTCTACGTATCTACCAATACGTAGAGGTGTTT<br>AAACCCCCGGTTCTCCGGGGGTTTTTCATTTCTAGGCGTGTTT<br>CACTGTGCGTGAGCGTCCACCGGCAGCCAAGAAATTTTGTGGT<br>AAATGGCGTGGTAAAATGTGAAATTACCACGCACGCCCTGAAA<br>CCGTTACCACGTTTTGAAGCCATGCCACTCACCGACACCGCAA<br>TTAAAGCCCTGAAACCAAAGGACAAAGCATACAAAGCTACCGA<br>CGGTGGCAGGCTGTATTTGCTCGTACGTCCGAACGGCTCGAAG<br>CTGTGGCAATGGGGCTATACGTTCTGTGGTAAAGCAAAGGTGC<br>TGTCATTTGGTGCCTACCCTGCAATTAGCCTGTCCGACGCCCG<br>AAAAAAGCGAGACGCCGCGAAGAAACTTCTATCGGATGGCACA<br>GACCCCGGCTTGCAGGCGAAGCTCGATAAGATTGCGACAAAAG<br>CAAGCACGGATGATTCGTTCAATGCTATCGCAGATGAATTCAT<br>TGCTAAGGCAAGGCGCGAAGGACTGGCAGCAGTTACCCTGAAA<br>AAAAGGAATGGCTGATAGGACTTGCAAGGCCAGATATAGGCAA<br>CCGGCCAATCGCTGACATTTCCGCCGCCGAGATTTTAATTCCA<br>TTACGGAAGGTCGAAGATCAGGGAAACTATGAAACGGCTCGAC<br>GGCTACGCTCCACCATCGGCCAGGTTTTCAGG<br>..<br>TGTCCTTGTTGTCGGCCTGCTTCTGGAGGGATTCAAGTTCGGC<br>ACGCGCCGATGCAAGTTCTTCCGCGGCCTTGGCCTCGGTCTTG<br>AGACGCTTGACGTCGGCAACCGTCACCTTCTGGCCGTCTACGA<br>TAAGCTCTTCGACTTGTTCGCGGACCGATTGCGGAGTGGACGG<br>GGCGGCGAGTTCGTAAAGGGCAGTCGCGTTCAAATCCGAAATC<br>GTTTCGGATTTGCCGTTGTAGTGCTCCGCAACGCGAATGAAGT<br>TCAATGCCGTGCGGTCGGTCATACGAAATTCAGCTTCGATCCA<br>CGGAAGGAACATACCGTGCGGAAGGCGTTCTTTCTGGCGCTTG<br>AGCGCCAAGCCTACGGCGATGATGCTTTCGGCGGCGGATTTCA<br>TATGGGCCTTGATGATCGCCGCATCTTCCTTGGCGGAAACTTC<br>ATCATCACGGCTCAAGGCCGCGTATGAAAAATGGACGTCGACC<br>GGATTTCCGGTAAACTCTTCAACGTCTGTTTGTTGGTTCATGG<br>GCACCACGTTCATGGGTGTGCTCCGGTTCAGGTGTTAGAATTT<br>TCAGGTTTGGTCGATGGGAAAATGTGCGGCGGATAAAGAACAG<br>GATCAATTTCGTATTGCAGCAACTATCACTGGACGCCTCGCCC<br>TTGAGCCTATAGAGCCAATTTTGCGAGAGACTTTTAGATTTTG<br>TGGTAAAAAACGTGGTAAAATAGCAATAAGCCAATAATATTTC<br>ACATATTTTACAAATAGATAGCGCGCATTTTGGAAGCCTCTTC<br>TGGCACCA		large 12kb insert region. Sequence have been truncated for legibility of the table.
1052790		1039855	1039855		T	between BMI_II1054/BRA1053 and BMI_II1055/BRA1054
1061628	1061646	1048692		GTTTAACCAAGAAGGCTGG		between BMI_II1063/BRA1063 and BMI_II1064/ BRA1064. Bruce75*
1079329		1066376	1066376		A	BMI_II1083/BRA1083 is pseudogene in B. microti and B. suis
1082153	1082157	1069199		TACGC		BMI_II1086/BRA1085 is pseudogene in B. suis, 2-oxoisovalerate dehydrogenase alpha and beta subunit
1095073	1095073	1082114		T		BMI_II1096/BRA1095 is pseudogene in B. suis, dipeptide transport ATP-binding protein dppF
1099903	1099903	1086943		A		BMI_II1101/BRA1100 is pseudogene in B. suis, oligopeptide ABC transporter ATP-binding protein
1104820	1104820	1091859		C		pseudogene BRA1104 ortholog BMI_II1105 is intact in B. microti, ABC transporter substrate-binding protein
1120053		1107093	1107106		GTGCTGTTCTCGCT	23S ribosomal rna, difference in IVS region
1120063		1107117	1107124		ACGCATTC	
1120090	1120103	1107150		CAATAAGGCAATAT		
1120116	1120117	1107162		CT		
1120137		1107183	1107183		G	
1120150		1107197	1107213		CCATCCAGCGTTGCTCC	
1123355	1123356	1110417		TA		BMI_II1124/BRA1118 is pseudogene in B. microti, hypothetical protein
1129995		1117057	1117058		GC	BMI_II1132/BRA1126 is pseudogene in B. microti, hypothetical protein
1149013	1149014	1136075		TC		between BMI_II1152/BRA1146 and BMI_II1153/BRA1147
1207416		1194478	1194478		T	between BMI_II1197/BRA1191 and BMI_II1198/BRA1192
