# Supplementary material for: Brucella microti: the genome sequence of an emerging pathogen
Source: BMC Genomics. 2009 Aug 4;10:352. doi: 10.1186/1471-2164-10-352 (PMC2743711; doi:10.1186/1471-2164-10-352)
Supplement: Additional file 4 — Supplementary Table 2: Distinctive VNTR typing of B. microti as confirmed by the genome sequence. For each tandem repeat we listed the primer pair, the theoretical length of the amplimer in B. suis 1330, B. melitensis 16M, B. abortus 9–941 and B. microti CCM 4915, and the location of the theoretical PCR product in the genome of B. microti. [file 1471-2164-10-352-S4.pdf]

VNTR name	alias name	upper primer	lower primer	size amplimer in *B. suis* 1330	size amplimer in *B. melitensis* 16M	size amplimer in *B. abortus* 9-941	size amplimer in *B. microti*	pos. min	pos. max	Chr.
BRU1543_8bp_152bp_2u	Bruce04 or TR6*	CTGACGAAGGGAAGGCAATAAG	CGATCTGGAGATTATCGGGAAG	184	152	160	**200**	440805	441005	bmi1
BRU1322_134bp_408bp_3u	Bruce06*	ATGGGATGTGGTAGGGTAATCG	GCGTGACAATCGACTTTTTGTC	274	408	542	**542**	665713	666255	bmi1
BRU1250_8bp_158bp_5u	Bruce07**	GCTGACGGGGAAGAACATCTAT	ACCCTTTTTCAGTCAAGGCAAA	166	158	150	**158** a)	739501	739658	bmi1
BRU1134_18bp_348bp_4u	Bruce08*	ATTATTCGCAGGCTCGTGATTC	ACAGAAGGTTTTCCAGCTCGTC	330	348	366	**366**	854581	854947	bmi1
BRU588_8bp_156bp_7u	Bruce09 or TR8*	GCGGATTCGTTCTTCAGTTATC	GGGAGTATGTTTTGGTTGTACATAG	140	156	124	**156**	1406877	1407033	bmi1
BRU211_63bp_257bp_2u	Bruce11*	CTGTTGATCTGACCTTGCAACC	CCAGACAACAACCTACGTCCTG	509	257	383	**887**	1785595	1786482	bmi1
BRU73_15bp_392bp_13u	Bruce12*	CGGTAAATCAATTGTCCCATGA	GCCCAAGTTCAACAGGAGTTTC	345	392	375	**390**	18101	18491	bmi2
BRU548_8bp_152bp_3u	Bruce16**	ACGGGAGTTTTTGTTGCTCAAT	GGCCATGTTTCCGTTGATTTAT	168	152	176	**232**	750610	750842	bmi2
BRU339_8bp_146bp_5u	Bruce18**	TATGTTAGGGCAATAGGGCAGT	GATGGTTGAGAGCATTGTGAAG	138	146	154	**178**	960267	960445	bmi2
BRU329_8bp_148bp_6u	Bruce21**	CTCATGCGCAACCAAAACA	GATCTCGTGGTCGATAATCTCATT	175	148	164	175	971139	971314	bmi2
BRU1505_8bp_151bp_6u	Bruce30 or TR2*	TGACCGCAAAACCATATCCTTC	TATGTGCAGAGCTTCATGTTCG	127	151	151	**143**	479749	479892	bmi1
BRU424_125bp_539bp_4u	Bruce42*	CATCGCCTCAACTATACCGTCA	ACCGCAAAATTTACGCATCG	538	539	289	**663**	1571524	1572187	bmi1
BRU379_12bp_182bp_2u	Bruce43*	TCTCAAGCCCGATATGGAGAAT	TATTTTCCGCCTGCCCATAAAC	170	182	182	**182**	1617168	1617350	bmi1
BRU233_18bp_151bp_3u	Bruce45*	ATCCTTGCCTCTCCCTACCAG	CGGGTAAATATCAATGGCTTGG	187	151	151	187	1763535	1763722	bmi1
BRU2066_40bp_273bp_3u	Bruce55*	TCAGGCTGTTTCGTCATGTCTT	AATCTGGCGTTCGAGTTGTTCT	234	273	273	**394**	2053514	2053908	bmi1
BRU324_6bp_163bp_18u	Bruce19	GACGACCCGGACCATGTCT	ACTTCACCGTAACGTCGTGGAT	169	163	184	**76**	975795	975871	bmi2
BRU1938_8bp_371bp_9u	Bruce01 or TR7	GGTCTGGGAAAACATGAAAAGC	AGCCGATCCTGCAAAACATAAT	395	371	419	**363**	65469	65832	bmi1
BRU1923_339bp_787bp_3u	Bruce02	AACGCAGCATCACCAATGT	CCCAGATGTTCGGCTATAGTATG	448	787	2143	**1126**	80507	81633	bmi1
BRU1627_9bp_199bp_3u	Bruce03	GGCTATTATTTCACCGGCAAGA	TCTTGATTTCCTTGCGATACCA	208	199	217	208	356122	356330	bmi1
BRU1365_8bp_185bp_3u	Bruce05	AAGTATCAGGAAGGGCAGGTTC	GGGAGTAGGGGAATAAGGGAAT	193	185	201	**201**	623505	623706	bmi1
BRU221_19bp_127bp_2u	Bruce10	ATCAATTCGCGGATATTTCACT	AGTGCGTTTCATATGTTTGCTG	146	127	165	146	1775861	1776007	bmi1
BRU19_8bp_196bp_2u	Bruce13	CGAACGATAGACCAGAACATGC	TTGAAAGAATCAGATAAGATAAAG	204	196	220	**252**	72915	73167	bmi2
BRU18_8bp_102bp_7u	Bruce14	TTGCTTTATCTTATCTGATTCTTTC	GGTGTCGTTGGAGATAGAGGTC	142	102	94	**94**	73140	73234	bmi2
BRU1112_264bp_346bp_1u	Bruce15	GCGGTGTTGTGTCTGTGGATA	GCCGTCAGTATCCACGTCATAG	875	346	4112	**1946**	157320	159266	bmi2
BRU344_5bp_110bp_3u	Bruce17	TTTTCACAGGGCATGTTCTCAG	CGCGTTTCGATTGTGGAAAATA	125	110	115	125	955496	955621	bmi2
BRU329_8bp_148bp_7u	Bruce20 or TR4	AATACTGGGTCCAGTCCGATG	AGCGCAGCGACCATATTCT	100	148	124	**140**	971001	971141	bmi2
BRU322_8bp_158bp_6u	Bruce22 or TR1	GATGAAGACGGCTATCGACTG	TAGGGGAGTATGTTTTGGTTGC	150	158	134	**142**	977173	977315	bmi2
BRU1990_9bp_152bp_1u	Bruce23	ATCAGCGAGTCGAAGGTCAGTT	TTCGACTATGCCAATCCAGATG	161	152	161	161	12340	12501	bmi1
BRU1940_8bp_146bp_8u	Bruce24 or TR5	AGGGGAGTATGTTTTTGGTTGC	GCTACAAGATCGAAGTGCTCCA	146	146	106	**218** a)	63607	63824	bmi1
BRU1915_8bp_215bp_2u	Bruce25 or TR3	GGGAGTATGTTTTGGTTGCACA	CTATTTCGTCCTGCCATTCGAC	239	215	239	**255**	88563	88818	bmi1
BRU1704_12bp_189bp_5u	Bruce26	TCTTCATCCTGCGAGATCATGT	ATTCGTGATCGGGGTGATGAT	162	189	189	**189**	279495	279684	bmi1
BRU1609_8bp_170bp_6u	Bruce27	TCGACGTCGTCTGACATTTTCT	GGGAGTAAGGCAGTAGGGGAAT	170	170	162	170	374054	374224	bmi1
BRU1599_11bp_147bp_4u	Bruce28	TATCTTCCACGGCCATGAATC	GGCAGGATCGGCGTATAGATAA	136	147	136	136	384149	384285	bmi1
BRU1528_15bp_132bp_3u	Bruce29	TTGCGTTATTGATTGTCAGCAC	GCTGTGGCTCGTCTATGTGG	132	132	117	132	455961	456093	bmi1
BRU1475_18bp_120bp_1u	Bruce31	GCTGAATCTTTTCCGCATCCT	GGAATCTCTGCACTGACAAAGC	138	120	120	138	509503	509641	bmi1
BRU1424_8bp_106bp_5u	Bruce32	AGGTTTCCGGCGATAATGG	TCGGGATGCGCTCTAGAATATC	106	106	98	106	561050	561156	bmi1
BRU1413_15bp_158bp_4u	Bruce33	GATGGAGCTTGGTTCCTGCTT	GCATGATCCGTTTTCTTCTCAA	143	158	143	**158**	571721	571879	bmi1
BRU1409_18bp_83bp_2u	Bruce34	GCGATCGAAGGAAATATCGAG	CGCTGCCGGGATGTGAAC	101	83	101	101	575638	575739	bmi1
BRU1282_10bp_136bp_4u	Bruce35	TGCGATAACAGGTGTACCCAAG	GACGGCAGCCATGCTGAT	116	136	116	115	706736	706851	bmi1
BRU1234_15bp_157bp_4u	Bruce36	TAAGGCTCTTGCGTTTGTATCG	TGCGTATCTTCAGACTGGCAAT	142	157	142	**157**	755417	755574	bmi1
BRU1176_21bp_124bp_2u	Bruce37	CCAAGCGTATCATCGATCTGTC	TCGGACGCAGATTGTTTCTATC	103	124	123	103	813023	813126	bmi1
BRU1116_18bp_108bp_2u	Bruce38	CTGAATTGGGAGGAGGAACCAG	AGCTGAACGACCTTGGCATCT	126	108	108	126	873172	873298	bmi1
BRU1112_15bp_164bp_7u	Bruce39	GAAGGTCTCGAAGGAAGAGCTG	CCATCCATATTGATCGTCAGGA	164	164	146	164	877054	877218	bmi1
BRU1048_15bp_94bp_2u	Bruce40	AAAAGAAGGGTTTCCCCATACC	GGAAAGGACAGCTTCGAGTACC	109	94	109	109	943919	944028	bmi1
BRU1030_13bp_94bp_1u	Bruce41	TTATGTCACCGCTGACGAATTT	CTCATTATGGACCCGGTCTTTC	107	94	107	107	962447	962554	bmi1
BRU256_12bp_110bp_3u	Bruce44	GGCGCAAGATCGGAATGC	AGGCAGGTGCTGATTCTCCT	110	110	122	110	1740715	1740825	bmi1
BRU217_15bp_256bp_4u	Bruce46	AAAAGCTTCCGAACCAAGTGTC	GGAGCTGGTTGAGCGTTATTTC	256	256	241	256	1780084	1780340	bmi1
BRU149_15bp_116bp_2u	Bruce47	CTGCCAAGGGCGAGATAAAC	CATCGTTCTGATCTTCGTGACC	131	116	131	131	1853528	1853659	bmi1
BRU131_29bp_131bp_2u	Bruce48	TATAAGTCCAGCCCATGACAGG	GCGGAATATCTGGATGGGATAC	131	131	160	**160**	1870748	1870908	bmi1
BRU112_13bp_266bp_2u	Bruce49	AACCTCGGTCTATGATGCAACC	ACGCAGGGTTAGGTTTCTCAAA	279	266	265	279	1889939	1890218	bmi1
BRU80_12bp_162bp_3u	Bruce50	GCAGAACCTGATGAACAACCTG	ATTTTCTGGTCGAGATCGAAGG	174	162	174	174	1921793	1921967	bmi1
BRU80_15bp_74bp_2u	Bruce51	TGACATGATGCAGAAAATGCAG	GTCCCTTGCCGCCTTTCAT	74	74	89	**89**	1922312	1922401	bmi1
BRU50_15bp_185bp_3u	Bruce52	CAATGAACCAGATCAGCTTTCG	CGCCATGGTTTCAATATCACC	170	185	170	170	1952021	1952191	bmi1
BRU50_30bp_110bp_1u	Bruce53	CGGTTATGGTGTGGAGCAACT	CTTCCAGCGGGCTTTCAG	140	110	110	140	1952208	1952348	bmi1
BRU28_13bp_150bp_1u	Bruce54	CCGATCACAGACACAACAACTTC	GCGAAAAGGGAGCAGACATTAT	163	150	163	163	1974332	1974495	bmi1
BRU2028_12bp_135bp_4u	Bruce56	TTGGTCGTTAGAACAAGAGTGG	CTGAACCTGTTCCGTCAAATCA	135	135	123	135	2091851	2091986	bmi1
BRU69_9bp_301bp_4u	Bruce57	ATGGGAGCCTATTTCGCTTACA	GGCGGTAGAATGGATAGCTCAC	292	301	292	292	22339	22631	bmi2
BRU33_24bp_98bp_1u	Bruce58	CATCCTGCTTGGTGTTCTTTTG	GATGGTCGTCACCAAGTCCAG	122	98	122	122	58549	58671	bmi2
BRU33_9bp_256bp_7u	Bruce59	CGTATCATCCGGCAATGGT	CTTTCTCTTTGTCGTGGGCTTC	265	256	256	265	58712	58977	bmi2
BRU24_12bp_279bp_3u	Bruce60	AGCAAATGAATATGTCGCGTTG	TTCACCCCGATATCGATGAAT	267	279	279	**279**	67698	67977	bmi2
BRU22_12bp_162bp_5u	Bruce61	CCTAATTTCGCCATTCGGTAAC	TTGCGGATTTTCCGAATAGAAC	162	162	150	162	69296	69458	bmi2
BRU979_18bp_140bp_3u	Bruce62	CTGACGCAGGGAAGCTTTGT	GAAAGAATGGTGAGCAGCAAGA	155	140	155	155	292440	292595	bmi2
BRU833_15bp_145bp_3u	Bruce63	AGGGTGACATTTGTTGGAGTCA	GTGGACAGACCCATGGTAAACG	160	145	145	160	457056	457216	bmi2
BRU832_14bp_104bp_1u	Bruce64	GAGACGACGCTTGAGGTTTTTC	CTTCCGGCGCTTCTTTCTTAT	118	104	118	118	457550	457668	bmi2
BRU824_41bp_182bp_2u	Bruce65	CGCTCTAGGCATAGCTGTTGTG	GTCCAGCCGATAACCTTGCTAT	143	182	143	143	466205	466348	bmi2
BRU652_17bp_91bp_1u	Bruce66	ATATCCATGCGGGAAGGAAGAT	TATGCAGTGGGCATTCTCTACG	108	91	91	**91**	646335	646426	bmi2
BRU609_31bp_155bp_2u	Bruce67	GCACCAGTGCGAGAAAATAGTG	AATTTTCCTTCGCGACTCCTTT	124	155	155	**155**	689168	689323	bmi2
BRU564_18bp_111bp_3u	Bruce68	GACAACGATCCGCAGAAAGG	GTCAATGCCCTGATCGGTATC	111	111	93	111	735102	735213	bmi2
BRU488_57bp_181bp_1u	Bruce69	CGATGACAGAGCAAGACCGTTA	TTGACCGATAATTCTGCAATGG	181	181	238	181	810919	811100	bmi2
BRU339_21bp_146bp_2u	Bruce70	GAGTAAGGCGAATAGGGGGAAC	AACTCTTCTTCCGCGACAACAC	146	146	162	**162**	960332	960494	bmi2
BRU337_12bp_394bp_3u	Bruce71	GAAGACGGCTATCGACTGGTCT	ACAAGCTCTATTCGCCTTACGC	370	394	370	**402**	977176	977578	bmi2
BRU322_8bp_230bp_8u	Bruce72	GAAGACGGCTATCGACTGGTCT	GTTTCAATGAAGGCGAGGTGAG	206	230	206	**238**	977176	977414	bmi2
BRU285_28bp_178bp_3u	Bruce73	GTGGAAGGCGTTGTCATTCTG	ATCGGTCATGGTCTATCCTTCC	178	178	150	178	1015022	1015200	bmi2
BRU275_8bp_147bp_6u	Bruce74	GGATGAGGATTGAGGGCTTTT	ACGCAGACGATTTAACAAAGCA	139	147	139	139	1024393	1024532	bmi2
BRU250_19bp_82bp_2u	Bruce75	AGGACTATCAGGTGCGTGACAA	AAGGAAGACGTCGCTGAAAGAC	82	82	101	**101**	1061585	1061686	bmi2
BRU181_14bp_122bp_2u	Bruce78	CTAACAAATGACGGCAGAGTGC	TTGAACGCAAGCTTATCCAAAA	136	122	136	136	1131177	1131313	bmi2
BRU163_12bp_141bp_4u	Bruce79	TCCTGTTGAACGCAAGCTAATG	ATACTTCAGGCGGGGAGGAC	153	141	153	153	1148830	1148983	bmi2
BRU542_12bp_178bp_4u	Bruce80	CGAGGAATGTCAGGAAGATCAC	ACACAGACGCCAAAAGACAAA	166	178	166	166	756795	756961	bmi2

a) Primer pair have one mismatch in B. microti genome.

Supplementary Table2
