# Supplementary figures and images for: Brucella microti: the genome sequence of an emerging pathogen
Source: BMC Genomics. 2009 Aug 4;10:352. doi: 10.1186/1471-2164-10-352 (PMC2743711; doi:10.1186/1471-2164-10-352)

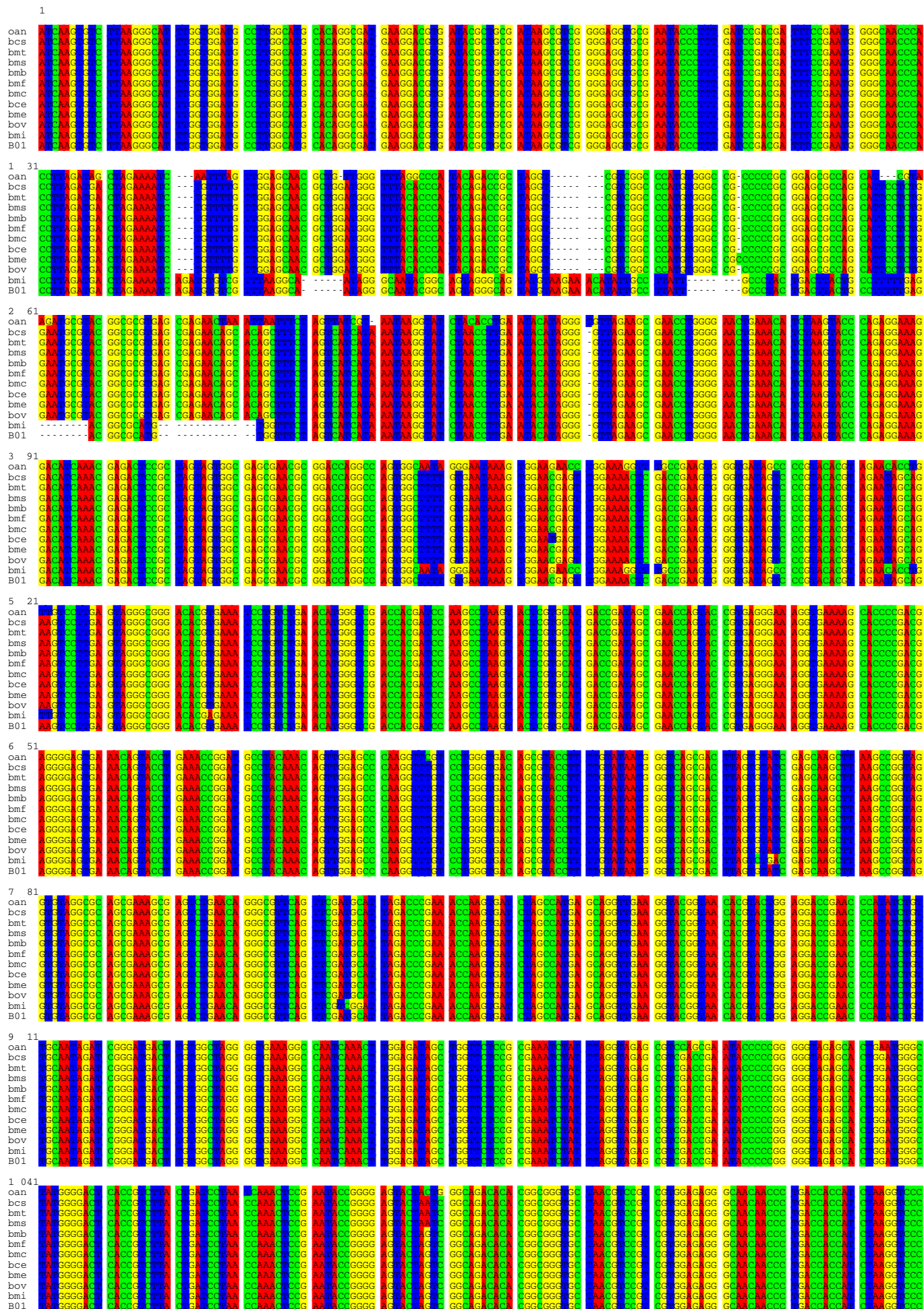

Supplementary Figure 2 (1/3)

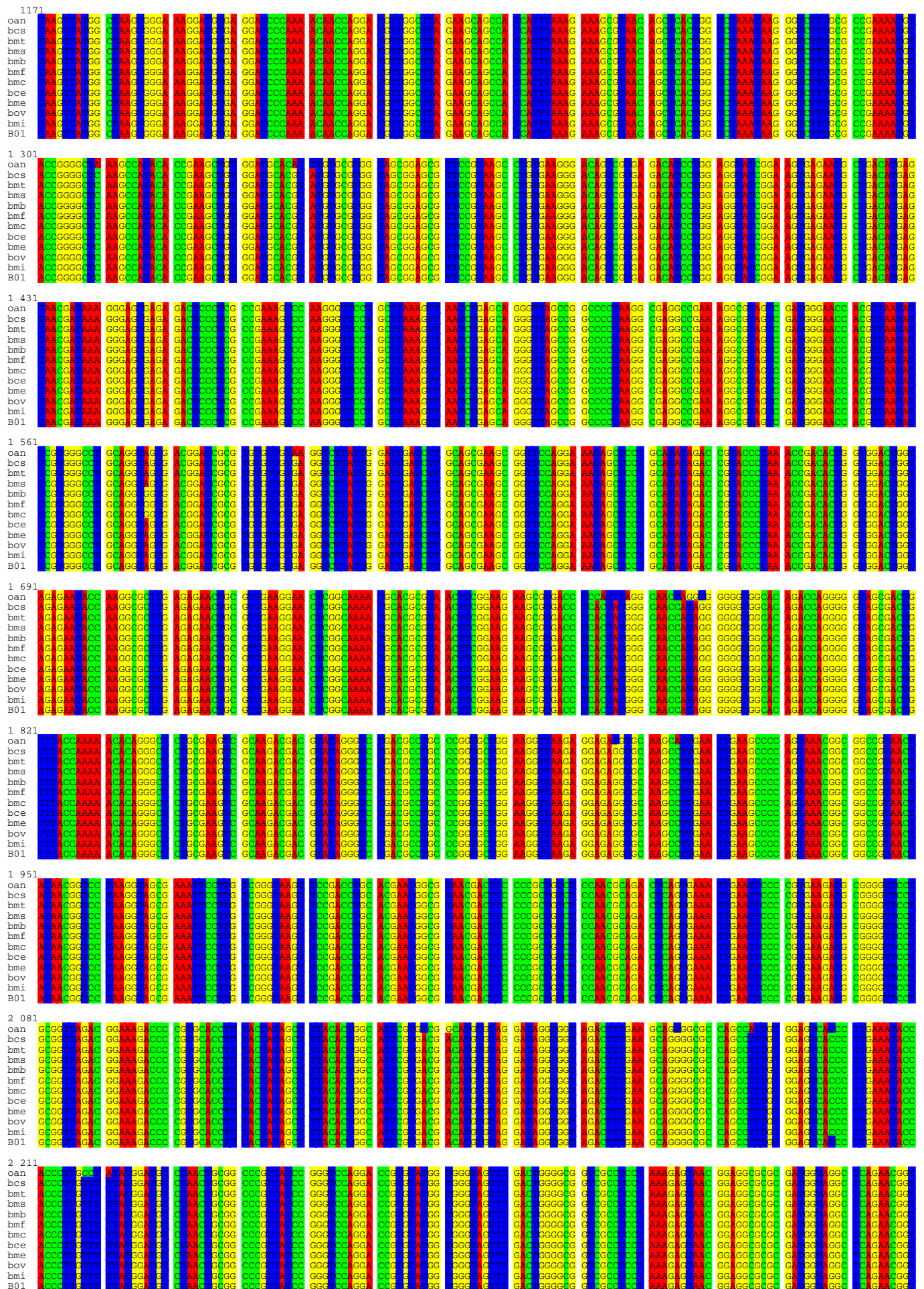

Supplementary Figure 2 (2/3)

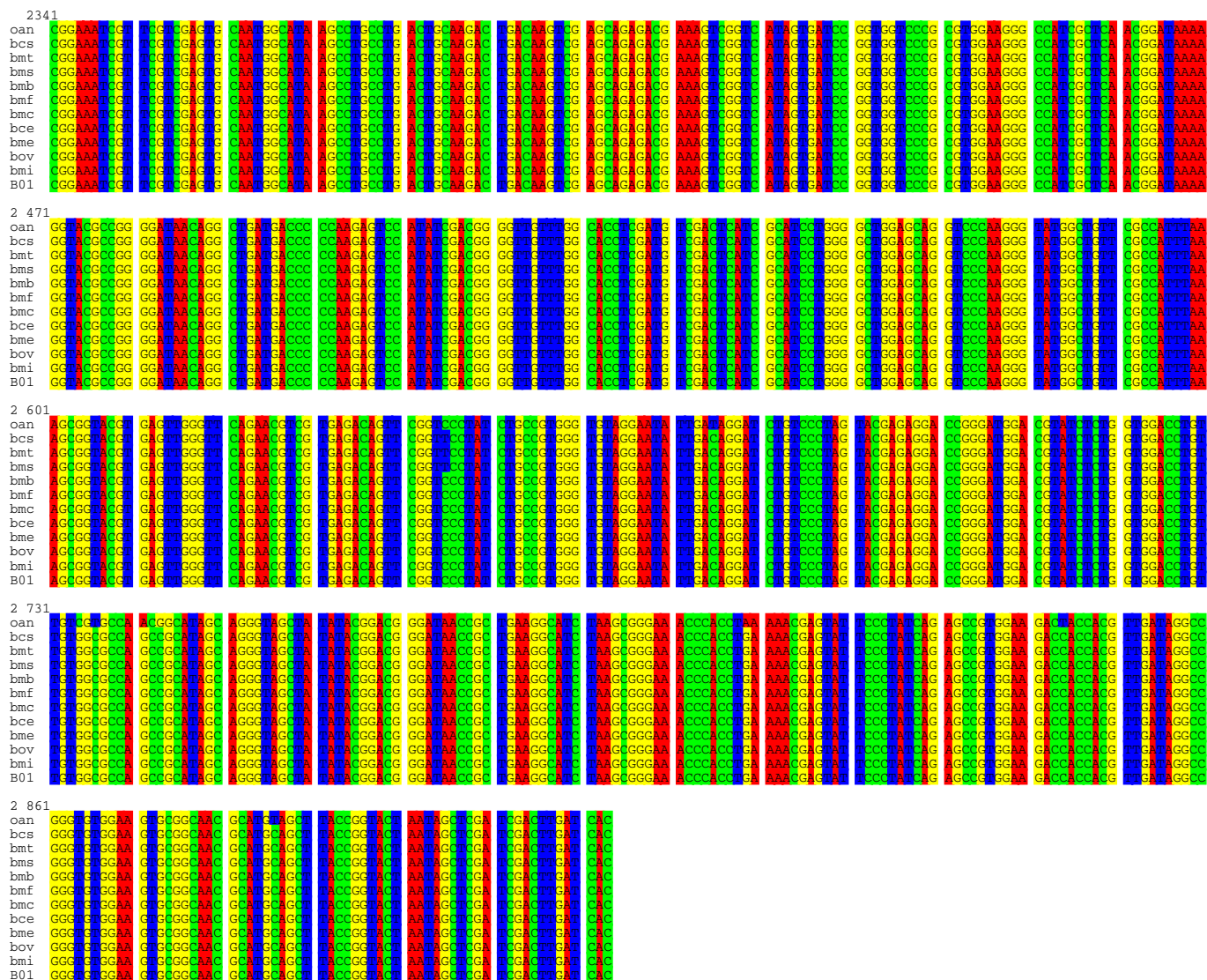

Supplement: Additional file 5 — Supplementary Figure 2: Alignment of the 23S ribosomal RNA gene sequences in O. anthropi and other Brucella studied in this work. In addition, we included the sequences of B. ceti and Brucella inopinata B01. Abbreviations: oan, O. anthropi; bcs, B. canis; bmt, B. suis ATCC 23445; bms, B. suis 1330; bmb, B. abortus 9–941; bmf, B. melitensis biovar abortus 2308; bmc, B. abortus S19; bce, B. ceti; bme, B. melitensis 16M; bov, B. ovis; bmi, B. microti and B01, Brucella inopinata B01. [file 1471-2164-10-352-S5.pdf]

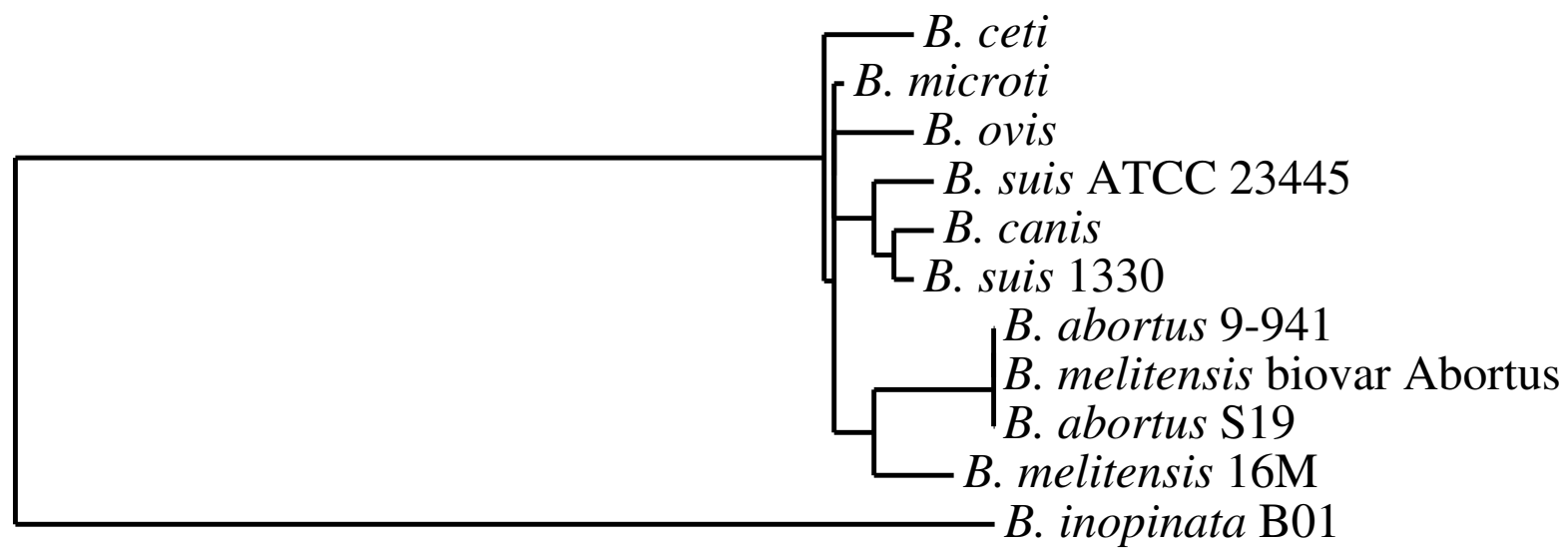

0.005 substitutions per site

Supplementary Figure 1

Supplement: Additional file 6 — Supplementary Figure 1: Phylogenetic representation of the alignment of the regions corresponding to the first 10,000 nucleotides of B. microti genome sequence, showing that Brucella inopinata sp. nov. strain B01 diverged earlier than the other Brucella studied in this work. The sequence of B. ceti is also included. B. ceti and Brucella inopinata B01 sequences were obtained from the PATRIC web site [18]. [file 1471-2164-10-352-S6.pdf]

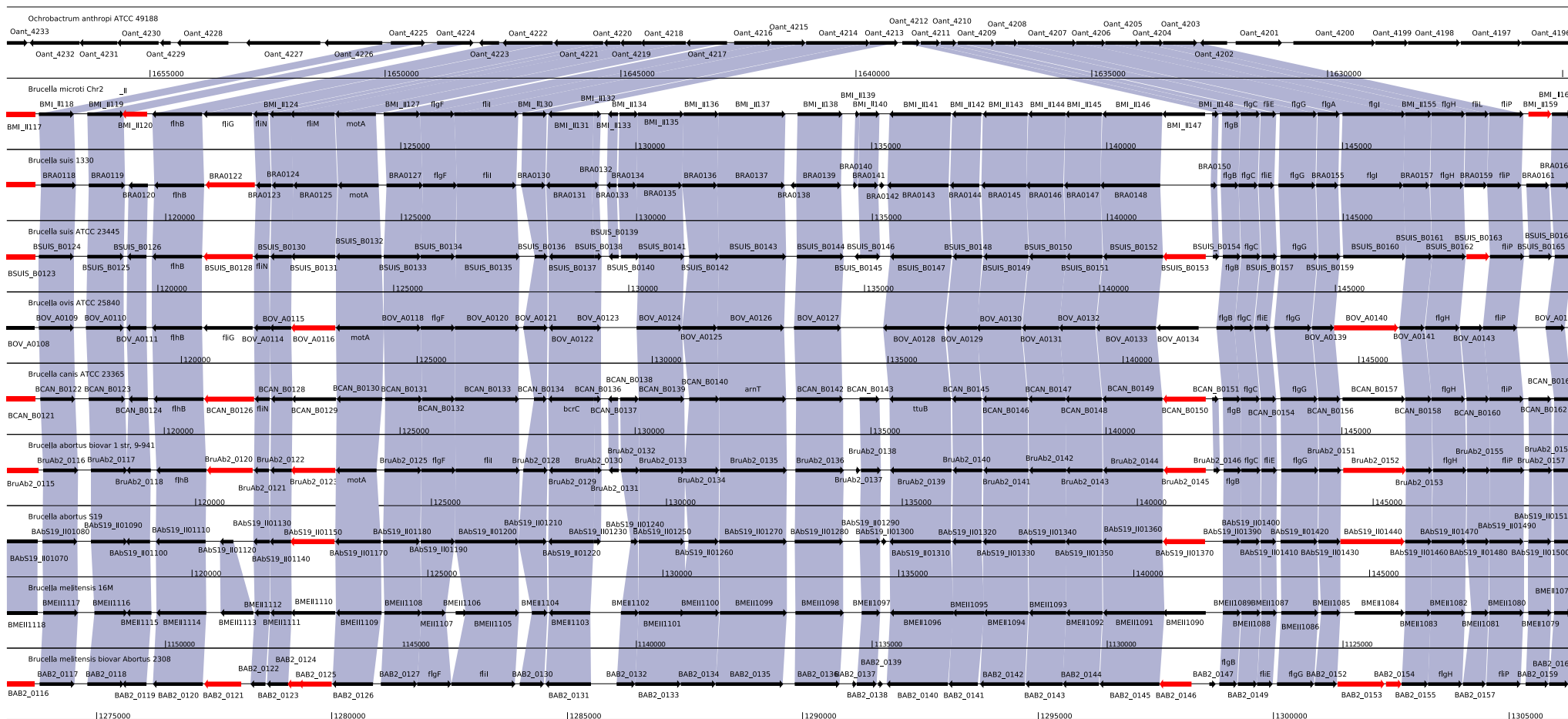

Supplementary Figure 3

Supplement: Additional file 9 — Supplementary Figure 3: Genomic representation of the region around the cluster of flagella assembly genes that is contiguous in O. anthropi and interrupted in Brucella. Intact genes are represented as black arrows, pseudogenes as red arrows. [file 1471-2164-10-352-S9.pdf]
