# Supplementary material for: Brucella microti: the genome sequence of an emerging pathogen
Source: BMC Genomics. 2009 Aug 4;10:352. doi: 10.1186/1471-2164-10-352 (PMC2743711; doi:10.1186/1471-2164-10-352)
Supplement: Additional file 7 — Supplementary Table 3: Extended list of orthologous genes exhibiting annotation differences between B. microti and B. suis 1330 (one, or both being annotated as pseudogene, or presenting notable difference). For each gene, we give its identification in B. microti and B. suis 1330 as well as its status in other Brucella. Abbreviations include: * for internal stop, a number indicates multiple stops, fs for frameshift, + for an intact sequence, Mult. Diffs for multiple difference, Mult. Fs for multiple frameshifts, NF for not found. [file 1471-2164-10-352-S7.pdf]

B. microti ID	status in *B. microti*	*B. suis* 1330 ID	status in *B. suis* 1330	status in *B. ovis* ATCC 25840	status in *B. suis* atcc 23445	status in *B. abortus* S19	status in *B. melitensis* biovar Abortus 2308	status in *B. abortus* biovar 1 str. 9-941	status in *B. melitensis* 16M	status in *B. canis* ATCC 23365	status in *O. anthropi* ATCC 49188	Annotation/comment
BMI_I17	+	BR0017	fs	+	+	+	+	+	+	fs	+	BMI_I17 ortholog is pseudogene BR0017, 3-hydroxy-3-methylglutarate-CoA lyase
BMI_I36	fs	BR0033	+	+	+	+	+	+	+	+	+	pseudogene BMI_I36 ortholog is gene BR0033, hypothetical protein
BMI_I75	+	BR0072	Mult. diffs	Mult. diffs	Mult. diffs	Mult. diffs	Mult. diffs	Mult. diffs	Mult. diffs	Mult. diffs	NF	B. microti gene is larger, outer membrane protein, quite variable among *brucella*
BMI_I78	+	BR0075	STOP	+	STOP	+	+	+	+	STOP	+	BMI_I78 ortholog is pseudogene BR0075, putative amino acid efflux protein, LysE family
BMI_I104	STOP	BR0101	+	STOP,fs	+	+	+	+	+	+	fs,STOP	pseudogene BMI_I104 ortholog is BR0101, hypothetical protein
BMI_I116	STOP	BR0113	+	fs	+	+	+	+	+	+	+	pseudogene BMI_I116 ortholog is gene BR0113, phenazine biosynthesis protein, PhzF family.
BMI_I133	+	BR0130	STOP	STOP	NF	+	+	+	+	+	+	BMI_I133 ortholog is pseudogene BR0130, alcohol dehydrogenase class III
BMI_I135	+	BR0132	fs	+	fs	fs	fs	fs	fs	fs	+	BMI_I135 ortholog is pseudogene BR0132, ATP-dependent helicase HrpB
BMI_I149	+	BR0146	fs	fs	fs	fs	fs	fs	fs	fs	+	BMI_I149 ortholog is pseudogene BR0146, malate dehydrogenase (oxaloacetate-decarboxylating) (NADP(+)), phosphate acetyltransferase
BMI_I195	fs	BR0192	+	fs	STOP	fs	fs	fs	fs	fs	NF	pseudogene BMI_I195 ortholog is BR0192, hypothetical protein
BMI_I201	STOP	BR0197	fs	fs	+	+	+	+	+	fs	+	pseudogene BMI_201 ortholog is pseudogene BR0197, transcriptional regulator, LuxR family. Intact gene is present in B. suis ATCC 23445, BSUIS_A0198
BMI_I205	+	BR0201	diff	diff	+	+	+	+	+	diff	diff	BMI_I205 ortholog is pseudogene BR0201, transcriptional regulator, Fis family
BMI_I209	+	BR0205	fs	+	+	+	+	+	+	fs	+	BMI_I209 ortholog is pseudogene BR0205, transcriptionnal regulator, lacI family
BMI_I210	+	BR0206	fs	deletion	deletion	diff	diff	diff	fs	fs	+	BMI_I210 ortholog is pseudogene BR0206, ABC transporter, HlyB/MsbA family
BMI_I316	fs	BR0310	+	+	+	+	+	+	+	+	+	pseudogene BMI_I316 ortholog is BR0310, DNA-damage-inducible protein F, putative
BMI_I322	STOP	BR0316	+	STOP	+	+	+	+	fs	+	+	pseudogene BMI_I322 ortholog is BR0316, sensor histidine kinase/response regulator

Supplementary Table 3 (1/11)

BMI_I345	+	BR0340	fs	+	+	+	+	+	+	+	+(2 copies)	BMI_I345 ortholog is pseudogene BR0340, invasion protein B
BMI_I346	fs	BR0341	fs	+	+	+	+	+	+	fs	+	pseudogene BMI_I346 ortholog is pseudogene BR0341, sensor histidine kinase, BSUIS_A0372 is intact
BMI_I376	+	BR0371	deletion +STOP	deletion	deletion	+	+	+	fs	deletion +STOP	+	BMI_I376 ortholog is pseudogene BR0371, transporter MFS superfamily
BMI_I417	+	BR0413	+	fs	+	diff. at the end	diff. at the end	diff. at the end	+	+	diff	BMI_I417 ortholog is pseudogene BR0413, GGDEF domain protein. It is not clear why this protein is annotated as a pseudogene in B. suis
BMI_I425	+	BR0421	fs	+	fs	+	+	+	+	fs	+	BMI_I425 ortholog is pseudogene BR0421, phosphoglycerate mutase family protein
BMI_I426	+	BR0423	fs	+	+	+	+	+	+	fs	+	BMI_I426 ortholog is pseudogene BR0423, cold shock protein CspA
BMI_I455	fs	BR0453	+	+	+	small deletion	small deletion	small deletion	+	+	+	pseudogene BMI_I455 ortholog is gene BR0453, hypothetical protein
BMI_I503	fs near the end	BR0501	STOP+fs near the end	diff at the end	STOP+fs near the end	bme	bme	bme	bme	NF	diff. at the end	BMI_I503 pseudogene corresponds to B. suis BR0501 pseudogene, putative polysaccharide deacetylase
BMI_I504	+	BR0502 and BR0503	fs	fs	fs	fs	fs	fs	+	fs	+	BR0502 and BR0503 match both BMI_I504 and are probably both pseudogenes, hypothetical protein
BMI_I506	fs	BR0505	+	+	+	+	+	+	+	+	+	pseudogene BMI_I506 ortholog is gene BR0505, metallo-beta-lactamase family protein
BMI_I554	fs	BR0555	+	+	+	+	+	+	+	+	NF	pseudogene BMI_I554 is gene BR0555, hypothetical protein
BMI_I559	bmi	BR0560	+	bmi	bmi	bmi	bmi	bmi	bmi	bmi	bmi	BMI_I559 ortholog is BR0560 in B. suis which is longer. It is a transcriptional regulator, GntR family. The difference is specific to B. suis 1330.
BMI_I595	+	BR0596	fs	fs	fs	fs	fs	fs	fs	fs	diff	BMI_I595 ortholog is pseudogene BR0596, hypothetical protein
BMI_I600	+	BR0601	fs	+	fs	STOP	STOP	STOP	fs	fs	NF	BMI_I600 ortholog is pseudogene BR0601, Phage Host Specificity Protein
BMI_I656	bmi	BR0657	deletion	bmi	deletion	bmi	bmi	bmi	bmi	deletion	bmi	BMI_I656 is longer than its ortholog BR0657, transcriptional regulator, AraC family
BMI_I731	fs	BR0733	+	+	+	+	+	+	+	+	NF	pseudogene BMI_I731 ortholog is gene BR0733, hypothetical protein
BMI_I794	fs	BR0794	+	fs	fs	fs	fs	diff	fs	fs	fs	pseudogene BMI_I794 ortholog is BR0794, hypothetical protein
BMI_I859	+	BR0862	fs	+	+	+	+	+	+	fs	diff	BMI_I859 ortholog is pseudogene BR0862, Bacterial extracellular solute-binding protein, family 3

BMI_I876	fs	BR0879	+	+	+	+	+	+	+	+	+	pseudogene BMI_I876 ortholog is BR0879, glycosyl hydrolase, family 3
BMI_I916	+	BR0917	annotated as pseudog.	+	+	+	+	+	+	+	+	BMI_I897 ortholog BR0917 is annotated as pseudogene, probably because programmes ribosomal frameshift was not recognized.
BMI_I947	+	BR0949	STOP	STOP	STOP	+	STOP	STOP	STOP	STOP	+ (diff)	BMI_I947 ortholog is pseudogene BR0949, membrane protein involved in aromatic hydrocarbon degradation
BMI_I977	truncated	BR0976	truncated	truncated	truncated	truncated	truncated	truncated	truncated	truncated	NF	pseudogene BMI_I977 is ortholog to pseudogene BR0976, both are duplicated and broken queuine tRNA-ribosyltransferase, with have an intact paralog elsewhere in the genome, probably resulting from the nearby insertion sequence
BMI_I980	fs	BR0979	+	NF	+	STOP	STOP	STOP	+	+	diff	pseudogene BMI_I980 ortholog is gene BR0979, hypothetical protein
BMI_I1019	+	Not annotated	+	+	+	+	+	+	fs	+	diff	BMI_I1019 ortholog is not annotated in B. suis, prolin-rich extensin
BMI_I1030	+	BR1027	STOP	+	STOP	fs	fs	fs	fs	STOP	+ (diff)	BMI_I1030 ortholog is pseudogene BR1027, putative thioredoxin reductase
BMI_I1041	+	BR1038	diff	bmi	bmi	bmi	bmi	bmi	bmi	bsu	diff	BMI_I1041 is longuer than BR1038, basic proline-rich protein precursor
BMI_I1045	+	BR1042	fs	fs	fs	+	+	+	+	fs	+	BMI_I1045 ortholog is pseudogene BR1042, mechanosensitive ion channel family protein
BMI_I1094	+	BR1083	truncated	diff	+	+	+	+	+	+	NF	BMI_I1094 ortholog BR1083 is shorter, BRO family, N-terminal domain protein
BMI_I1102	fs	BR1090	+	+	+	+	+	+	+	+	+	pseudogene BMI_I1102 ortholog is BR1090, transcriptional regulator, TetR family
BMI_I1113	+	BR1101	stop+fs	fs	stop+fs	+	+	+	fs	stop+fs	NF	BMI_I1113 ortholog is much shorter in B. suis (BR1101). Hypothetical protein
BMI_I1139	fs	BR1127	+	+	+	+	+	+	+	+	+	pseudogene BMI_I1139 ortholog is BR1127, pyruvate dehydrogenase complex, E2 component, dihydrolipoamide acetyltransferase
BMI_I1199	STOP	BR1188	diff	diff	+	+	+	+	+	diff	+	pseudogene BMI_I1199 ortholog is pseudogene BR1188, propionyl-CoA carboxylase, beta subunit, putative, Oant_2003 is intact
BMI_I1267	+	BR1255	fs	+	+	+	+	+	+	fs	+	BMI_I1267 ortholog is pseudogene BR1255, CBS domain containing protein
BMI_I1326	+	BR1313	fs	+	STOP	+	+	+	+	STOP	+	BMI_I1326 ortholog is pseudogene BR1313, acyl-CoA dehydrogenase

BMI_I1329	+	BR1316/ BR1317	likely differenc e at the start codon	+	+	+	+	+	+	likely difference at the start codon	diff	BMI_I1312 correspond to gene BR1316/BR1317. Entericidin EcnAB
BMI_I1330	+	BR1318	STOP	+	+	+	+	+	+	STOP	+	BMI_I1330 ortholog is pseudogene BR1318, transcriptional regulator, LysR family
BMI_I1332	+	BR1320	STOP	fs	+	STOP	STOP	STOP	STOP	STOP	+	BMI_I1332 ortholog is pseudogene BR1320, sarcosine dehydrogenase
BMI_I1334	+	BR1322	fs	fs	+	+	+	+	+	fs	diff	BMI_I1334 ortholog is pseudogene BR1322, MscS mechanosensitive ion channel
BMI_I1379	fs	BR1369	+	+	+	+	+	+	+	+	NF	pseudogene BMI_I1379 ortholog is gene BR1369, crcB family protein
BMI_I1386	+	BR1376	STOP,fs	fs	STOP	+	+	+	+	STOP,fs	+	BMI_I1386 ortholog is pseudogene BR1376, Endonuclease/exonuclease/phosphatase family protein
BMI_I1460	+	BR1448	fs	+, with small deletion	fs	+	+	+	fs	fs	+	BMI_I1460 ortholog is pseudogene BR1448, lytic murein transglycosylase
BMI_I1461	+	BR1449	fs	+	+	+	+	+	+	+	+	BMI_I1461 ortholog is pseudogene BR1449, Auxin Efflux Carrier
BMI_I1483	+	BR1471	fs	fs	fs	fs	fs	fs	fs	fs	NF	BMI_I1483 ortholog is gene BR1471 which is much smaller. This gene is also , also broken in other Brucella, but it is not clear if this is a real gene.
BMI_I1527	+	BR1513	+	+	+	+	+	+	+	+	diff	BMI_I1527 ortholog is pseudogene BR1513, transglycosylase. Not clear why it is annotated as pseudogene in B. suis
BMI_I1558	+	BR1544	fs	+	+	+	+	+	+	+	+	BMI_I1558 ortholog is pseudogene BR1544, ABC transporter, permease/ATP-binding protein
BMI_I1559	+	BR1545	STOP	+	+	+	+	+	+	+	+	BMI_I1559 ortholog is pseudogene BR1545, ABC transporter, permease/ATP-binding protein
BMI_I1561	+	BR1547	STOP	+	fs	+	+	+	+	+	+	BMI_I1561 ortholog is pseudogene BR1547, putative purine nucleoside permease
BMI_I1566	+	BR1552	fs	fs	fs	fs	fs	fs	fs	fs	+	BMI_I1566 ortholog is pseudogene BR1552, aspartyl/asparaginyl beta-hydroxylase
BMI_I1597	fs, (small insert)	BR1584	+	+ (small insert)	+ (small insert)	+ (small insert)	+ (small insert)	+ (small insert)	+ (small insert)	+	+ (small insert)	pseudogene BMI_I1597 ortholog is gene BR1584, dipeptide ABC transporter, permease protein
BMI_I1599	+	BR1586	STOPs (2)	STOP, fs	STOP (1)	STOPs (2)	STOPs (2)	STOPs (2)	STOP (1)	STOPs (2)	+	BMI_I1599 ortholog is pseudogene BR1586, extracellular solute-binding protein family 5, dppA
BMI_I1601	fs	BR1588	+	fs	+	+	+	+	+	+	NF	pseudogene BMI_I1601 ortholog is gene BR1588, hypothetical protein

BMI_I1643	+	BR1627	+	+	+	+	+	+	+	+	+ (diff at the beg)	BMI_I1643 and BMI_I1644 correspond to pseudogene BR1627 in B. suis, It is not clear why it is annotated as a pseudogene in B. suis.
BMI_I1644	+		+	+	+	fs	fs	fs	+	+	+	
BMI_I1645	+	BR1628	+	+	+	+	+	+	+	+	+	BMI_I1645 and BMI_I1646 correspond to pseudogene BR1628 in B. suis, It is not clear why it is annotated as a pseudogene in B. suis.
BMI_I1646	+		+	+	+	+	+	+	+	+	+	
BMI_I1654	+	BR1636	fs	+	+	+	+	+	fs	fs	diff	BMI_I1654 ortholog is pseudogene BR1636, choline dehydrogenase
BMI_I1693	+	BR1670	STOP	+	+	STOP, fs	STOP, fs	STOP,fs	STOP, fs	+	+	BMI_I1693 ortholog is pseudogene BR1670, Macrolide export ATP-binding/permease protein macB
BMI_I1694	fs	BR1671	+	+	+	+	+	+	+	+	+	pseudogene BMI_I1694 ortholog is gene BR1671, HlyD family secretion protein
BMI_I1726	fs	BR1706	+	+	+	+	+	+	+	+	+	pseudogene BMI_I1726 ortholog is gene BR1706, opgC protein, putative
BMI_I1737	+	BR1718	fs	+	fs	+	+	+	+	fs	+ (small deletion)	BMI_I1737 ortholog is pseudogene BR1718, transcriptional regulator, GntR family
BMI_I1788	+	BR1770	fs	+ (small deletion)	+	+	+	+	+	+	+	BMI_I1788 ortholog is pseudogene BR1770, ATP-dependent helicase
BMI_I1798	+	BR1780	fs	+	+	fs	fs	fs	+	+	+	BMI_I1798 ortholog is pseudogene BR1780, membrane protein MosC
BMI_I1805	+	BR1788	fs	+	fs	+	+	+	+	fs	+	BMI_I1805 ortholog is pseudogene BR1788, putative ATP-binding component of ABC transporter
BMI_I1810	+	BR1793	fs	+	+	+	+	+	+	+ (small deletion)	+	BMI_I1810 ortholog is pseudogene BR1793, D-amino acid dehydrogenase small subunit
BMI_I1862	+	BR1846	fs	diff	fs	fs	fs	fs	fs	different in the middle	NF	B. microti gene BMI_I1862 is longer than BR1846, hypothetical protein
BMI_I1873	fs	BR1856	+	+	+	+	+	+	+	+	+	pseudogene BMI_I1873 ortholog is BR1856, hypothetical protein
BMI_I1903	+	BR1881	fs	+	fs	+	+	+	+	+	+	BMI_I1903 ortholog is pseudogene BR1881, major facilitator superfamily transporter
BMI_I1915	+	BR1894	fs	+	+	+	+	+	+	+	+	BMI_I1915 ortholog is pseudogene BR1894, transcriptional regulator, GntR family
BMI_I1931	+	BR1909	diff	+	+	+	+	+	+ (small deletion)	+	+ (small deletion)	BMI_I1931 ortholog is pseudogene BR1909, hypothetical protein
BMI_I1976	+	BR1954	fs	+	+	fs	fs	+	+	+	NF	BMI_I1976 ortholog is pseudogene BR1954, amino acid ABC transporter, permease protein

BMI_I1989	+	BR1967	STOP	+	+	fs	+	fs	+	+	+ (slight difference at the beginning)	BMI_I1989 ortholog is pseudogene BR1967, cysteine synthase A
BMI_I2058	fs	BR2037	+	+	fs	+	+	+	STOP	+	+ (diff at the end)	pseudogene BMI_I2058 ortholog is gene BR2037, Peptidase, M20/M25/M40 family
BMI_I2154	fs	BR2132	+	+	+	fs	fs	fs	STOP	+	+	pseudogene BMI_I2154 ortholog is gene BR2132, Mg chelatase-related protein
BMI_I2174	STOP	BR2153	+	+	+	+	+	+	+	+	+ (small diff at the beg.)	pseudogene BMI_I2174 ortholog is pseudogene BR2153, hypothetical protein
BMI_I2199	+	BR2178	smaller	smaller	smaller	smaller	smaller	smaller	smaller	smaller	+	BMI_I2199 orthologs in other brucella are smaller, hydrolase

BMI_II85	fs	BRA0083	+	+	+	+	+	+	+	+	+	pseudogene BMI_II85 ortholog is BRA0083, hypothetical protein
BMI_II86	+	BRA0084	fs	+	+	+	+	+	+	+	+ (slight difference at the beginning)	BMI_II86 ortholog is BRA0084, 2-dehydro-3-deoxyphosphogluconate aldolase/4-hydroxy-2-oxoglutarate aldolase
BMI_II87	+	BRA0085	STOPs (2)	+	STOP	+	+	+	+	STOPs(2)	+	BMI_II87 ortholog is pseudogene BRA0085, hypothetical protein
BMI_II95	impaired	BRA0093/ BRA0094/ BRA0095	impaired	impaired	impaired	impaired	impaired	impaired	impaired	impaired	impaired	pseudogene BMI_II95 is annotated as genes BRA0093-94-95 in B. suis, pseudogene corresponding to mes:Meso_1225, hypothetical protein
BMI_II117	fs	BRA0117	diff	fs	fs	+	+	+	fs	STOP+fs	NF	pseudogene BMI_II117 ortholog is pseudogene BRA0117, HlyD family secretion protein pseudogene, BruAb2_0094
BMI_II120	fs	BRA0120	fs	fs	fs	+	+	+	+	fs	diff	pseudogene BMI_II120 ortholog is gene BRA0120, hypothetical protein, BMEII1115 seems to be the intact gene
BMI_II122	+	BRA0122	STOP (small insert)	+ (small insert)	STOP (small insert)	multiple fs (small insert)	multiple fs (small insert)	fs (small insert)	fs (small insert)	STOP (small insert)	+ (small insert)	BMI_II122 ortholog is pseudogene BRA0122, flagellar motor switch protein FliG
BMI_II147	+	BRA0149	difference in the middle of the gene	+	fs	fs	fs	fs	+	fs	NF	BMI_II147 ortholog is pseudogene BRA0149, 3-hydroxyisobutyrate dehydrogenase family protein

BMI_II159	STOP at the beg. of gene	BRA0161	+	+	+	+	+	+	+	+	NF	pseudogene BMI_II159 ortholog is gene BRA0161, hypothetical protein
BMI_II170	+	BRA0172/ BRA0173	mult. diff.	mult. diff.	mult. diff.	mult. diff.	mult. diff.	mult. diff.	mult. diff.	mult. diff.	mult. diff.	BMI_II170 outer membrane autotransporter, similar to mes:Meso_3532
BMI_II187	+	BRA0190	STOP	fs	STOP	+	+	+	+	STOP	NF	BMI_II187 ortholog is pseudogene BRA0190, glucose/galactose transporter
BMI_II202	+	BRA0206	+	+	+	+	+	+	+	+	fs	BMI_II202 ortholog is pseudogene BRA0206, RNA pseudouridylate synthase family protein, it is not clear why it is a pseudogene in B. suis 1330
BMI_II204	+	BRA0208	fs	+	+	+ (small diff at the beginning)	+ (small diff at the beginning)	+ (small diff at the beginning)	+ (small diff at the beginning)	+	+	BMI_II204 ortholog is pseudogene BRA0208, peptidase M16 domain protein
BMI_II205	+	BRA0208	fs	+	fs	STOP,fs	STOP,fs	STOP,fs	+	fs	+	BMI_II205 ortholog is pseudogene BRA0208, peptidase M16 domain protein
BMI_II222	+	BRA0225	fs , small deletion	+	fs , small deletion	+	+	+	+	+ small deletion	NF	BMI_II222 ortholog is pseudogene BRA0225, FMN-binding oxidoreductase
BMI_II226	+	BRA0229	STOP	+	STOP	+	+	+	+	STOP	+	BMI_II226 ortholog is pseudogene BRA0229, two component response regulator
BMI_II266	+	BRA0272	fs	+	+	+	+	+	+	+	+	BMI_II266 ortholog is pseudogene BRA0272, ABC transporter related protein
BMI_II277	+	BRA0282	+	+	+	+	+	+	+	+	+	BMI_II277 ortholog is pseudogene BRA0282, pseudoazurin (Cupredoxin) (Blue copper protein), not clear why it is a pseudogene in B. suis
BMI_II323	fs	BRA0328	+	fs	+	+	+	+	+ (diff at the end)	+	STOP at the beg	pseudogene BMI_II323 ortholog is gene BRA0328, spermidine/putrescine ABC transporter, permease protein, putative
BMI_II334	+	BRA0338	STOP	+ (diff at the beg)	+	STOPs (2)	STOPs(2)	STOPs(2)	STOP	STOP	NF	BMI_II334 ortholog is pseudogene BRA0338, glutamate decarboxylase beta
BMI_II335	+	BRA0339	fs	fs	fs	+	+	+	+	fs	NF	BMI_II335 ortholog is pseudogene BRA0339, glutamate/gamma-aminobutyrate antiporter
BMI_II391	+	BRA0394	fs	+	+	+	+	+	+	fs	diff	BMI_II391 ortholog is pseudogene BRA0394, branched-chain amino acid ABC transporter, permease/ATP-binding
BMI_II400	fs	BRA0403	+	fs	fs	fs	fs	fs	fs	fs	NF	pseudogene BMI_II400 ortholog is gene BRA0403, oxidoreductase, Gfo/Idh/MocA family

BMI_II409	+	BRA0412	STOP	+	+	+	+	+	+	+	NF	BMI_II409 ortholog is pseudogene BRA0412, hypothetical protein
BMI_II417	+	BRA0420	STOP	+	+ slightly shorter	NF	NF	NF	+	+	+	BMI_II417 ortholog is pseudogene BRA0420, glycosil transferase
BMI_II422	+	BRA0425	STOP	+	STOP	NF	NF	NF	+	STOP	+	BMI_II422 ortholog is pseudogene BRA0425, hypothetical protein
BMI_II461	+	BRA0464	fs	+	fs	fs	fs	fs	+	fs	+	BMI_II461 ortholog is pseudogene BR0464, daunorubicin resistance ATP-binding protein
BMI_II462	STOP	BRA0465	+	+	+	+	+	+	+	+	+	pseudogene BMI_II462 ortholog is gene BR0465, daunorubicin resistance transmembrane protein pseudogene
BMI_II467	+	BRA0470	fs	+ small deletion	fs, small deletion	+ small deletion	+ small deletion	+ small deletion	+ small deletion	fs	+, diff at the beginning	BMI_II467 ortholog is pseudogene BRA0470, transcriptional regulator, MarR family
BMI_II547	+	BRA0553	prematur e stop	fs	+	NF	NF	NF	+	premature stop	NF	BMI_II547 hemagglutinin ortholog is BRA0553, which is shorter than its B. microti counterpart.
BMI_II640	fs	BRA0643	+	fs		+	+	+	+	+	+ (STOP at the end)	pseudogene BMI_II640 ortholog is gene BRA0643, 3-oxoadipate enol-lactone hydrolase
BMI_II642	fs	BRA0645	+	STOP	NF	+	+	+	STOP	diff	+	pseudogene BMI_II642 ortholog is gene BRA0645,protocatechuate 3,4-dioxygenase, beta subunit
BMI_II645	fs	BRA0648	+	+	NF	+	+	+	+	+	+	pseudogene BMI_II645 ortholog is gene BRA0648, amino acid ABC transporter, periplasmic amino-acid binding protein
BMI_II656	fs + diff (end)	BRA0659	+	diff (beg)	diff (beg)	diff(beg)	diff (beg)	diff (beg)	diff (beg)	+	diff (beg)	pseudogene BMI_II656 (large deletion) ortholog is gene BRA0659, transporter, TrkA family
BMI_II660	+	BRA0663	fs	fs	fs	+	+	+	+	+	diff at the beg.	BMI_II660 ortholog is pseudogene BRA0663, hypothetical protein
BMI_II664	+	BRA0667	fs	+	+	+	+	fs	fs	+	+ diff at the beg.	BMI_II664 ortholog is pseudogene BRA0667, aminotransferase, class IV
BMI_II683	STOP,f s	BRA0688	+	+	+	+	+	+	+	+	NF	pseudogene BMI_II683 ortholog is gene BRA0688, hypothetical protein
BMI_II684	+	BRA0690	diff	+	+ (small deletion)	+	+	+	+	+	+	BMI_II684 ortholog is pseudogene BRA0690, Maltose/maltodextrin import ATP-binding protein
BMI_II690	+	BRA0696	fs	+	+	+	+	+	+	+	+	BMI_II690 ortholog is pseudogene BRA0696, formate dehydrogenase accessory protein
BMI_II695	fs	BRA0701	+	+	+	+	+	+	+	+	+	pseudogene BMI_II695 ortholog is gene BRA0701, iron compound ABC transporter, ATP-binding protein, putative

BMI_II707	fs,STOP	BRA0713	fs,STOP	fs,STOP	fs,STOP	+	+	+	STOP(near the end)	fs,STOP	STOP (near the end)	pseudogene BMI_II707 ortholog is pseudogene BRA0713, myo-inositol catabolism IolC protein pseudogene, intact BruAb2_0517
BMI_II710	+	BRA0716	diff at the beginning	+	+	+	+	+	+	+	+	BMI_II710 ortholog is pseudogene BRA0716, Myo-inositol catabolism IolB domain protein
BMI_II711	STOP	BRA0717	+	+	+	+	+	+	+	+	+	pseudogene BMI_II711 ortholog is gene BRA0717, inositol monophosphatase family protein
BMI_II715	+	BRA0722	+	fs	+	+	+	+	+	+	+	BMI_II715 ortholog is pseudogene BRA0722, proline dehydrogenase/delta-1-pyrroline-5-carboxylate dehydrogenase, not clear why it is annotated as pseudogene in B. suis
BMI_II769	+	BRA0776	STOP	+	STOP	+	+	+	+, 2 small deletions	STOP	different at the beginning	BMI_II769 ortholog is pseudogene BRA0776, multi-drug resistance efflux protein
BMI_II773	+	BRA0780	fs	diff at the beginning	fs	+	+	+	+	+	+	BMI_II773 ortholog is pseudogene BRA0780, phosphogluconate dehydratase
BMI_II776	+	BRA0783	+	fs	+	+	+	+	+	+	+, beginning is different	BMI_II776 ortholog is pseudogene BRA0783, oligopeptide transport ATP-binding protein OppF
BMI_II777	+	NA	+	+	+	+	+	+	+	+	+	BMI_II777 ortholog is not annotated in B. suis, oligopeptide transport ATB-binding protein
BMI_II792	+	BRA0798	fs	+	+	fs	fs	fs	fs	+	NF	BMI_II792 ortholog is pseudogene BRA0798, transcriptional regulator, LysR family
BMI_II802	+	BRA0808	fs	+, with deletion	STOP	+	+	+	fs	+	+	BMI_II802 ortholog is pseudogene BRA0808, ABC transporter, permease protein
BMI_II805	+	BRA0811	fs	+	fs	+	+	+	+	fs	diff	BMI_II805 ortholog is pseudogene BRA0811, mannitol dehydrogenase
BMI_II809	+	BRA0815	fs	+	fs	+	STOP	STOP	+	fs	+	BMI_II809 ortholog is pseudogene BR0815, putative multidrug efflux protein
BMI_II815	+	BRA0822	small deletion	+	small deletion	small deletion	small deletion	small deletion	+	small deletion	diff	BMI_II815 longer than BRA0822, small deletion in B. suis, hypothetical protein
BMI_II825	+	not annotated	+	+	+	+	+	+	+	+	fs	BMI_II825 ortholog is not annotated; putative phage-associated protein.
BMI_II831	+	not annotated	+	+	+	+	+	+	+	+	NF	BMI_II831 ortholog is not annotated in B. suis, hypothetical protein

BMI_II838	STOP	BRA0844	+	+	+	+	+	+	+	+	-	pseudogene BMI_II838 ortholog is gene BR0844, hypothetical protein
BMI_II839	+	BRA0845	fs	fs	+	+	+	+	+	+	-	BMI_II839 ortholog is pseudogene BR0845, hypothetical protein
BMI_II848	+	BRA0854	fs	+	+	+	+	+	+	fs	+	BMI_II848 ortholog is pseudogene BRA0854, oxidoreductase, FAD-binding
BMI_II875	+	BRA0881	fs	+	fs	+	+	+	+	fs	41 premier aa diff	BMI_II875 ortholog is pseudogene BRA0881, putative citrate lyase, beta subunit
BMI_II883	fs	BRA0889	+	+	+	+	+	+	+	+	+	pseudogene BMI_II883 ortholog is gene BRA0889, ada regulatory protein, putative
BMI_II938	+	BRA0944	fs	+	fs	+	+	+	+	fs	+	BMI_II938 ortholog is pseudogene BRA0944, transcriptional regulator, GntR family
BMI_II971	+	BRA0978	fs	+	+	+	+	+	+	+	+	BMI_II971 ortholog is pseudogene BRA0978, beta-lactamase
BMI_II978	+	BRA0985	STOP	STOP	STOP	STOP	STOP	STOP	STOP	STOP	+	BMI_II978 ortholog is pseudogene BRA0985, transcriptional regulator, MarR family
BMI_II1000	fs	BRA1007	fs	fs	fs	fs	fs	fs	+	+	+	pseudogene BMI_II1000 ortholog is pseudogene BRA1007, glutamyl-tRNA(gln) amidotransferase subunit A pseudogene, intact in B. canis BCAN_B1027
BMI_II1027	+	BRA1033	+	+	+	+	+	+	+	+	+	BMI_II1027 ortholog is pseudogene BRA1033, tRNA pseudouridine synthase A, not clear why it is annotated as pseudogene in B. suis.
Genes BMI_II1049 to BMI_II1053 have no counterpart in *B. suis* 1330 genome. This is the region of the 12kb insertion.												
BMI_II1084	fs	BRA1083	fs	-	fs	+	+	+	+	+	diff	pseudogene BMI_II1084 ortholog is pseudogene BRA1083, alpha/beta hydrolase fold:biotin/lipoyl attachment:esterase/lipase/thioesterase, active site, 2-oxo acid dehydrogenase pseudogene
BMI_II1086	+	BRA1085	fs	diff	+	fs	fs	fs	+	+	fs+diff	BMI_II1086 ortholog is pseudogene BRA1085, 2-oxoisovalerate dehydrogenase alpha and beta subunit
BMI_II1096	+	BRA1095	fs	diff	fs	+	+	+	+	+	diff	BMI_II1096 ortholog is pseudogene BRA1095, dipeptide transport ATP-binding protein dppF
BMI_II1101	+	BRA1100	fs	diff	+	+	+	+	+	+	diff	BMI_II1101 ortholog is pseudogene BRA1100, oligopeptide ABC transporter ATP-binding protein
BMI_II1105	+	BRA1104	fs	diff	+	+	+	+	+	+	diff	BMI_II1105 ortholog is pseudogene BRA1104, ABC transporter substrate-binding protein

BMI_II1108	+	BRA1107	STOP	truncated	stop+fs	truncated	truncated	truncated	truncated	truncated	-	BMI_II1108 ortholog is pseudogene BRA1107, spermidine/putrescine transport ATP-binding protein PotA
BMI_II1115	+	BRA1114	+	truncated	+	+	+	+	+	+	-	BMI_II1115 ortholog is pseudogene BRA1114, D-lactate dehydrogenase, why is it a pseudogene in B. suis?
BMI_II1124	fs	BRA1118	+	+	+	+	+	+	+	+	+	pseudogene BMI_II1124 ortholog is BRA1118, N-acetylglucosamine kinase
BMI_II1132	fs	BRA1126	+	+	+	+	+	+	fs	fs	+	pseudogene BMI_II1132 ortholog is BRA1126, hypothetical protein
BMI_II1149	+	BRA1143	STOP	+	+	+	+	+	+	+	+	BMI_II1149 ortholog is BRA1143, chemotaxis protein MotC
BMI_II1166	+	BRA1160	STOP	fs	+	+	+	+	+	STOP	-	BMI_II1166 ortholog is pseudogene BRA1160, homoprotocatechuate 2,3-dioxygenase
BMI_II1201	+	BRA1195	STOP	+	+	+	+	+	+	STOP	NF but paralogs	BMI_II1201 ortholog is pseudogene BRA1196, high-affinity branched-chain amino acid transport system permease protein

Supplementary Table 3: List of genes being impaired either in *B. microti* or *B. suis* with status of the reading frame in other *Brucella* and *Ochrobactrum anthropi*.
