# Supplementary material for: Brucella microti: the genome sequence of an emerging pathogen
Source: BMC Genomics. 2009 Aug 4;10:352. doi: 10.1186/1471-2164-10-352 (PMC2743711; doi:10.1186/1471-2164-10-352)
Supplement: Additional file 8 — Supplementary Figure 4: Phylogenetic tree for the 4 genes conserved in B. microti and O. anthropi and impaired in the other Brucella. The trees were built using the Phylogeny.fr Web Server [65] using defaults settings. A) BMI_I149, malate dehydrogenase (oxaloacetate-decarboxylating) (NADP+) and its paralog BMI_I1020 intact in other Brucella; B) BMI_I1566, aspartyl/asparaginyl beta-hydroxylase; C) BMI_I1599, extracellular solute-binding protein belonging to an ABC-type transport system involved probably in dipeptide transport and D) BMI_II978, MarR family transcriptional regulator. [file 1471-2164-10-352-S8.pdf]

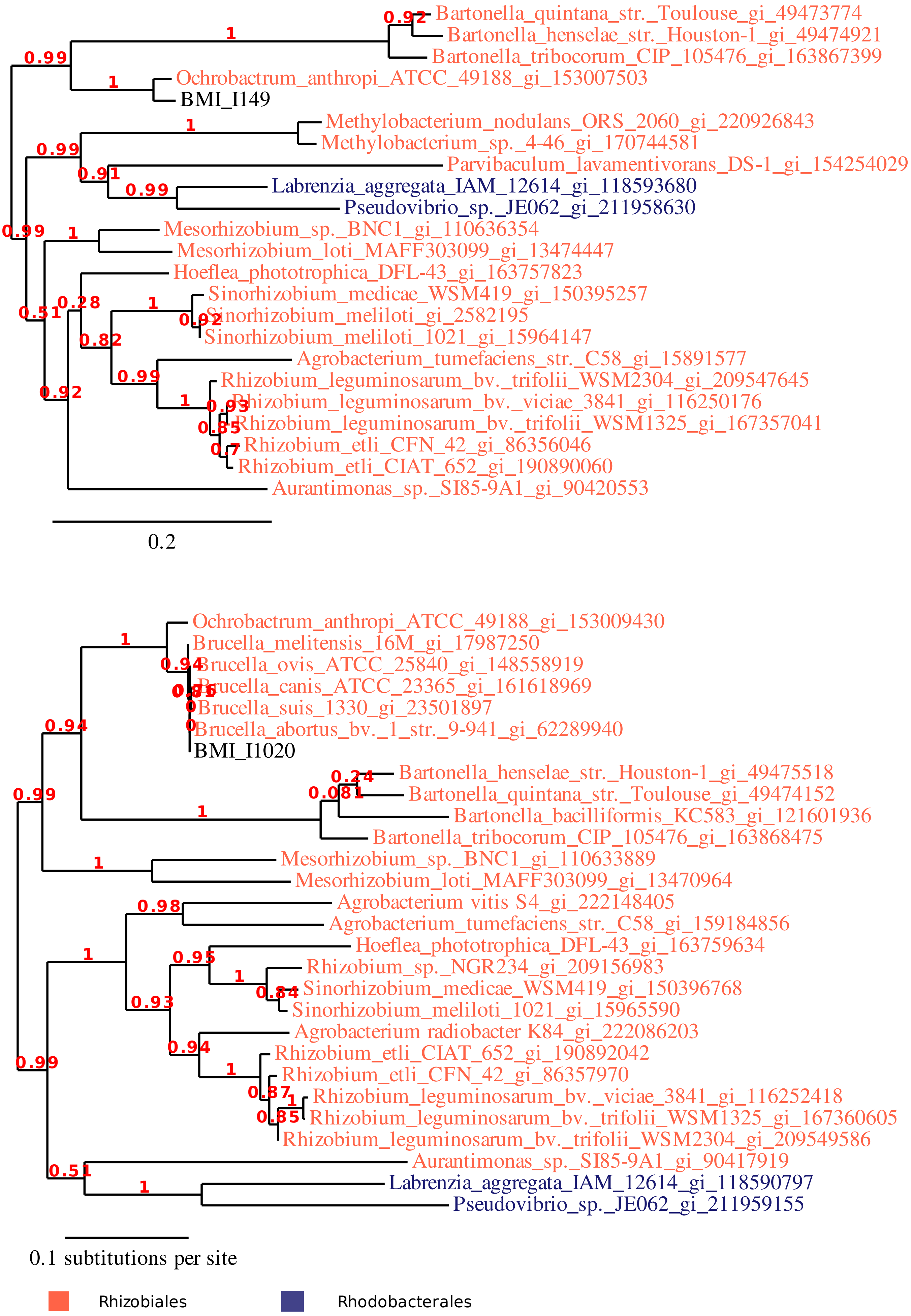

Supplementary Figure 4A

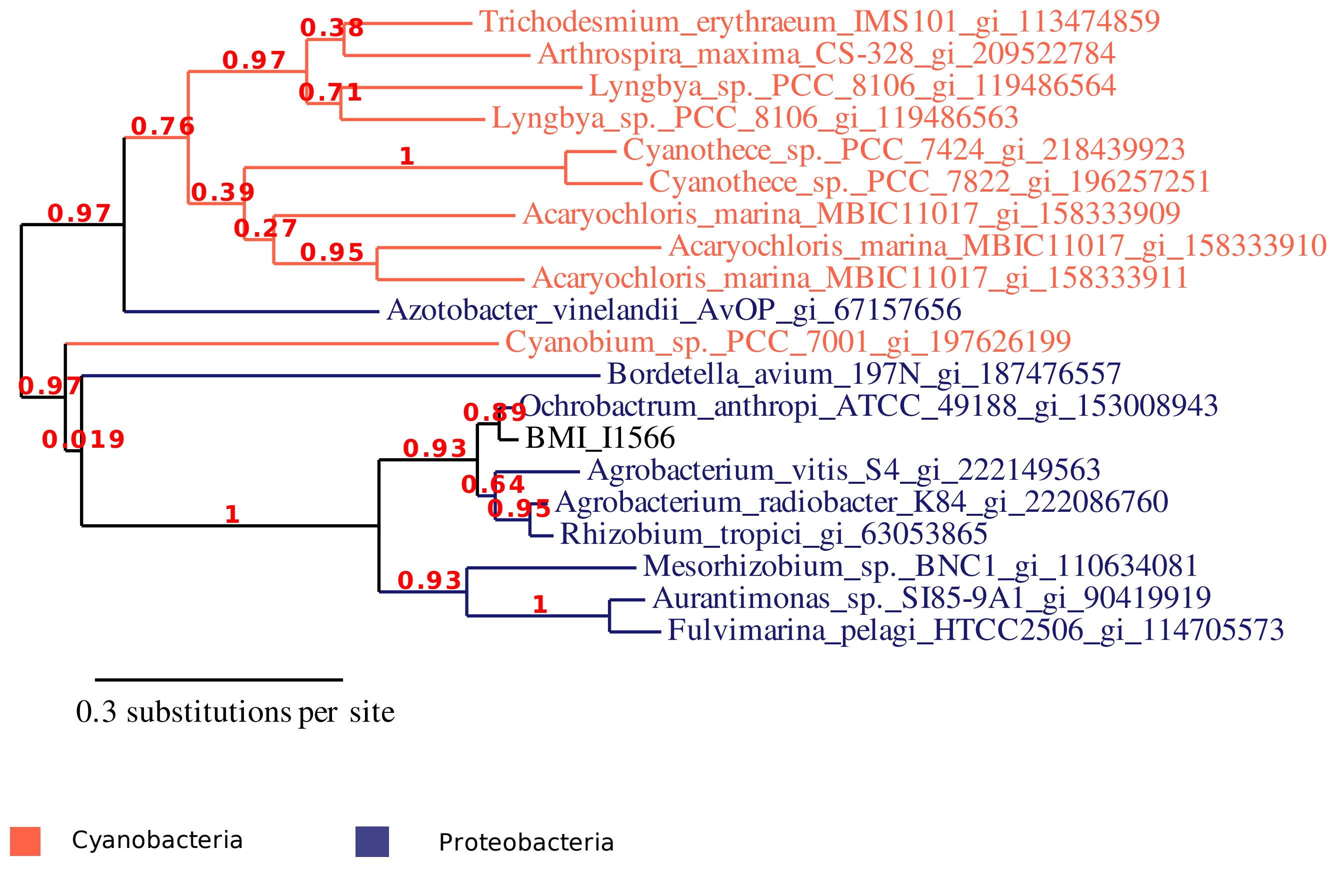

Supplementary Figure 4B

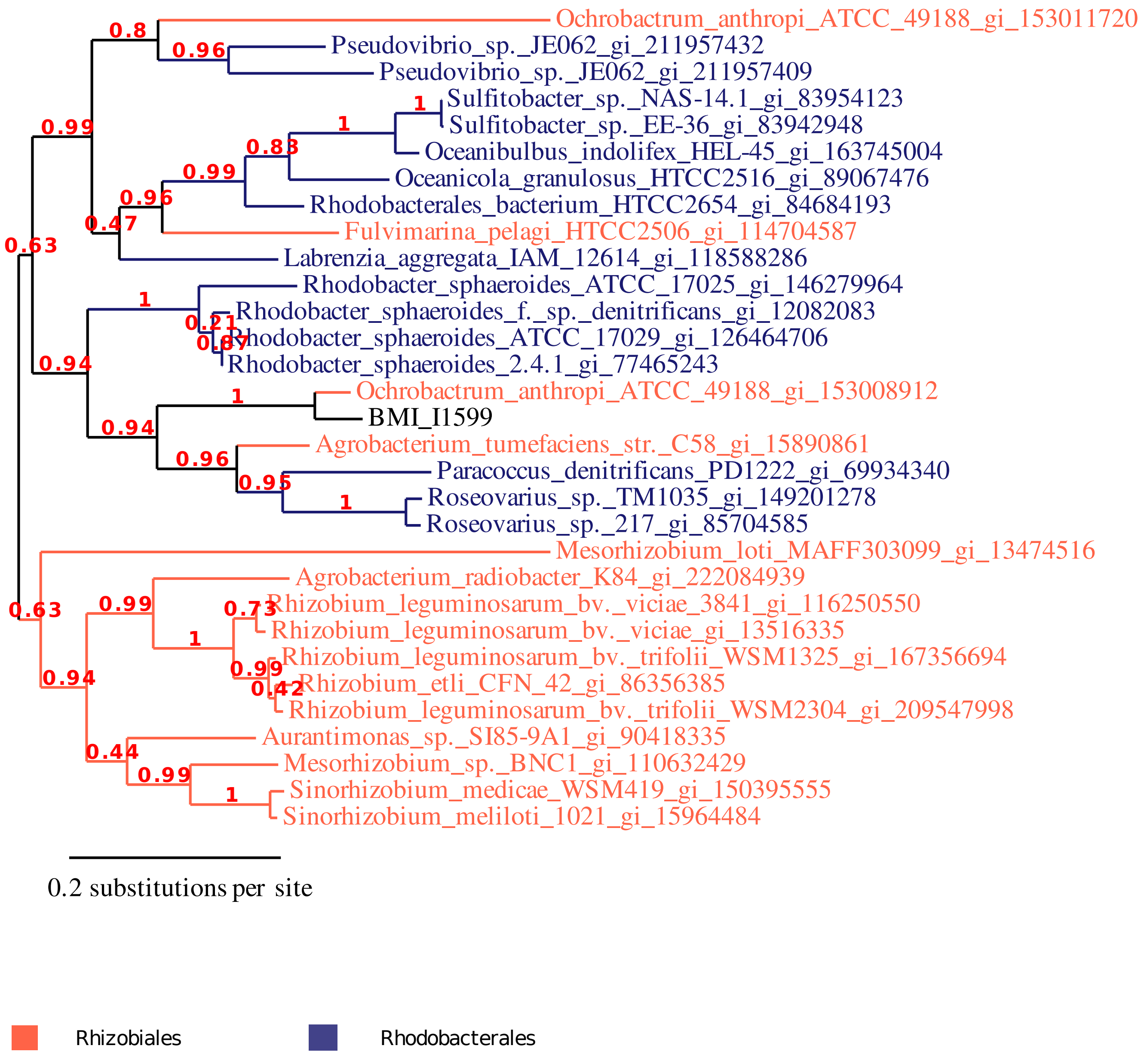

Supplementary Figure 4C

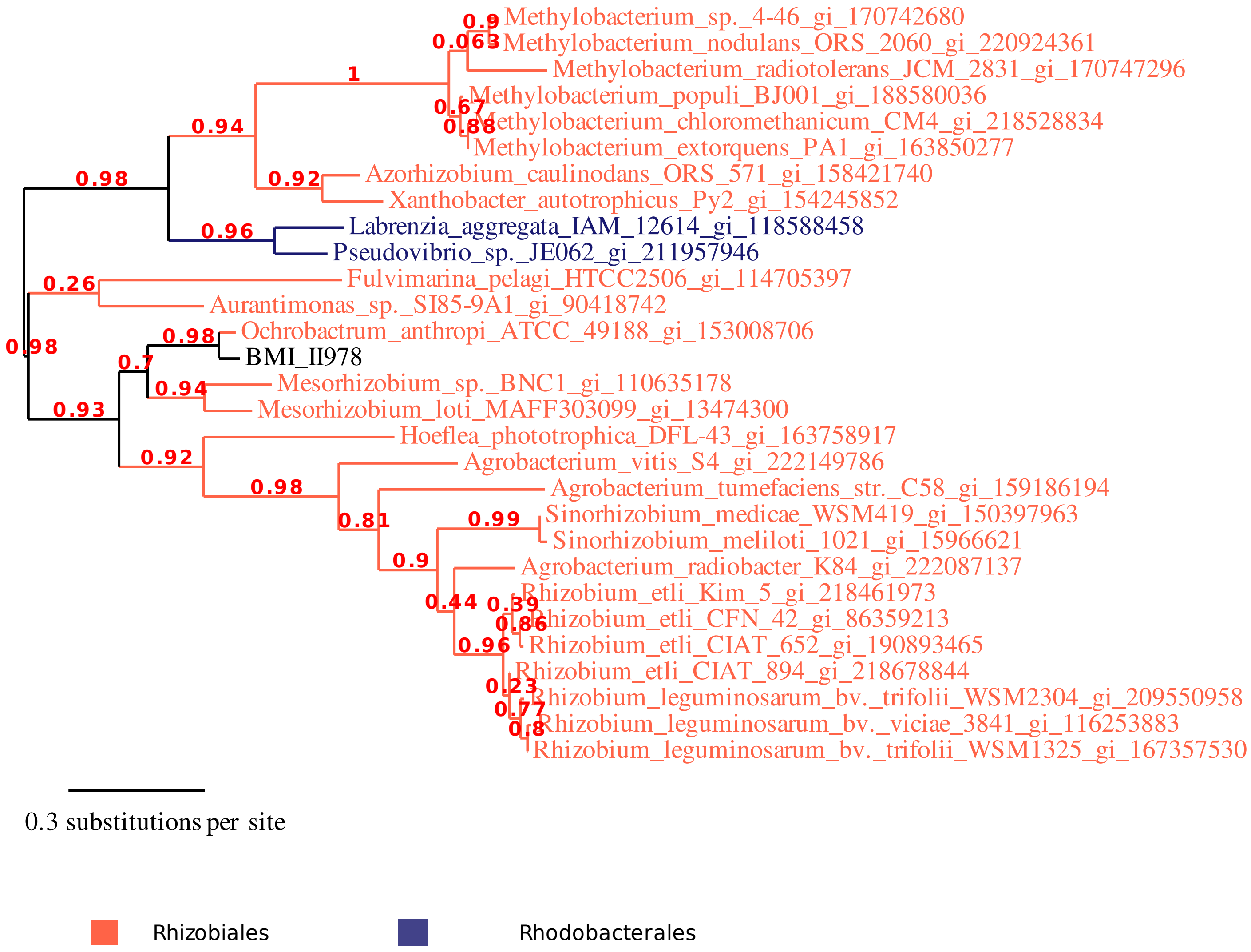

Supplementary Figure 4D
